# Hierarchical feature binding in a spiking neural network model of the primate ventral visual pathway

**DOI:** 10.1371/journal.pcbi.1014752

**Published:** 2026-09-16

**Authors:** Brian Gardner, Patrick T. McCarthy, Joseph Chrol-Cannon, Dan F. M. Goodman, Simon R. Schultz, Giovanni Lo Iacono, Simon M. Stringer

**Affiliations:** 1 Department of Comparative Biomedical Sciences, School of Veterinary Medicine, University of Surrey, Guildford, United Kingdom; 2 Centre for Neural Circuits and Behaviour, University of Oxford, Oxford, United Kingdom; 3 Independent Researcher, Guildford, United Kingdom; 4 Department of Electrical and Electronic Engineering, Imperial College London, South Kensington, London, United Kingdom; 5 Department of Bioengineering, Imperial College London, South Kensington, London, United Kingdom; 6 The Surrey Institute for People-Centred AI, University of Surrey, Guildford, United Kingdom; 7 Centre for Theoretical Neuroscience and Artificial Intelligence, Department of Experimental Psychology, University of Oxford, Oxford, United Kingdom; Research Center Jülich, GERMANY

## Abstract

Feature binding - how the brain encodes which features are part of other features to form representations of the coherent objects we perceive - remains an unsolved problem in neuroscience. Despite progress towards a solution, major theories either lack detailed explanations at the neuronal level or rely on biologically unrealistic simplifications, and none adequately account for the representation of hierarchical information, which is crucial to our perception of the world. To address this, a solution termed *binding by polychrony* has been proposed to explain how hierarchical feature relationships may be encoded at the neuronal level in a biologically realistic system. This theory relies on a phenomenon known as polychronization, where groups of neurons fire in precisely coordinated, time-locked sequences, leading to the emergence of regularly repeating spatiotemporal patterns that might encode these relationships. In this study, we explore binding by polychrony through simulations of a spiking neural network that closely aligns with the structural organisation of the primate ventral visual pathway, incorporating bottom-up, top-down, and lateral synaptic connections. By exposing the network to collections of related 2D object shapes from ecologically realistic datasets and applying spike-timing-dependent plasticity, the network self-organises such that individual neurons respond selectively to specific shape features. Furthermore, the network exhibits polychronization, giving rise to repeating spatiotemporal patterns, some of which form circuits that encode hierarchical feature relationships. Notably, these circuits are robust, even with the randomised, Poisson-distributed spike timings that represent the visual stimuli in the input layer. These results provide evidence for binding by polychrony as a feasible solution to the feature binding problem, and characterise the mechanism by which it may function. This mechanism can guide experimentalists in identifying such circuits *in vivo*, and could also be utilised in computer vision systems to capture more information and improve robustness to adversarial inputs.

## Author summary

How the brain pieces together visual details into coherent objects, known as the feature binding problem, remains an unresolved conceptual challenge in neuroscience. Existing theories either lack biological realism or do not adequately capture the hierarchical relationships among visual features. To address this shortcoming, we conducted detailed simulations with biologically realistic neuron models organised into networks reflecting the structure and connectivity of the primate ventral visual pathway. When exposed to ecologically realistic datasets of 2D object shapes and trained through unsupervised competitive learning, the networks self-organised such that neurons became selectively responsive to specific shape features. Crucially, we observed that groups of neurons developed precise, regularly repeating spatiotemporal firing patterns, a phenomenon known as polychronization. These patterns support a novel and biologically plausible mechanism through which neurons encode hierarchical relationships among visual features represented across different spatial scales within the network. This hierarchical feature binding mechanism advances our understanding of how complex visual information may be assembled in the brain. These insights could also inform the development of AI vision systems that interpret natural scenes more robustly and efficiently, similar to biological visual systems.

## Introduction

### Overview and aims

Eguchi, Isbister, and colleagues [1,2] have advanced a theory of *hierarchical* feature binding. These authors described how the feature *binding problem*, in the context of vision, concerns how the visual system represents a hierarchy of features (from edges to objects) at different spatial scales, and how it encodes the binding relationships between them (e.g., “this edge is part of that object”). Put another way, feature binding refers to how the visual system encodes which features are part of other features, dynamically linking them to form coherent object representations. Such hierarchical feature binding is thought to support the semantic interpretation of visual scenes, and may contribute to robust object recognition. Analogous hierarchical binding mechanisms are also likely to operate in other sensory domains such as audition. For example, in the human auditory system, phonemes may be bound to the words they form, which may in turn be bound to the identity of a particular speaker.

Various theoretical frameworks address mechanisms of feature binding in the brain [3,4]. A remaining challenge is to specify how binding can be implemented in hierarchical neural circuits in a way that scales across spatial levels and remains compatible with realistic axonal delays and spiking dynamics. Although binding may also be supported within single neurons via nonlinear dendritic integration [5,6], we consider the complementary question of how “part-of” relations can be encoded at the level of population activity and between-layer interactions.

The aim of this modelling study is to synthesise core aspects of prior related work. That is, we model the emergence of visual neurons representing a hierarchy of boundary contour elements of 2D shapes as previously performed by [7], but within more biologically realistic spiking neural network (SNN) models [1,2]. This enables us to investigate the simultaneous emergence of a hierarchy of boundary contour features, polychronization across network layers, and the formation of hierarchical feature binding (HFB) circuits containing binding neurons that relate contour features across increasing spatial scales. The use of a structured boundary-shape ensemble, which we describe as an ecologically realistic dataset in the sense of its feature statistics, provides a systematic test case for these mechanisms, and enables closer comparison with neuronal response properties reported in experimental studies [8–10].

Our primary advance beyond [1,2] is to move from a proof-of-principle demonstration of hierarchical binding by polychronization to a systematic test of the mechanism under a larger and more structured object ensemble. Specifically, we train and analyse the same type of hierarchical SNNs on diverse boundary-shape datasets, and we quantify how feature selectivity and polychronization co-develop across layers. We also focus explicitly on three-neuron HFB circuits, using their circuit-level timing constraints to identify binding relationships and to characterise where and how such motifs emerge throughout the hierarchy.

We next provide biological motivation for hierarchical feature representations and feature binding in the primate ventral visual system, before outlining the modelling context and the specific binding-by-polychrony mechanism examined here.

### Hierarchy and feature binding in biological vision

Human visual experience is hierarchical in nature [11]. That is, we perceive a scene as composed of a hierarchy of discrete but related visual features and objects at different spatial scales. For example, when we look at a face, we can identify constituent features such as the eyes and mouth as discrete features which are separate but related by the fact that they are part of the face, which is itself a larger scale discrete object. This cognitive process of binding smaller scale visual features to the larger scale visual features of which they are a part operates at every spatial scale across the visual field. Such semantically rich hierarchical feature binding underpins our ability to interpret and make sense of complex natural scenes.

The term “hierarchy” is used in several distinct ways in systems neuroscience, and this can invite overinterpretation if left implicit [12]. In this paper, we use hierarchy primarily in a *functional* sense: representations become progressively more abstract across successive processing stages, with features at smaller spatial scales providing constituent parts for features at larger scales. In the taxonomy of [12], this aligns most closely with a *progression of scales* that describes nested feature representations. As such, our model targets how multi-scale feature representations can be learned and related by binding operations across stages, rather than making claims about any single anatomical definition of hierarchy.

The need for explicit binding mechanisms is highlighted by classic theoretical arguments. When multiple objects are present, distributed feature representations can become ambiguous, entailing the “superposition catastrophe” in which the system cannot determine which features belong together [13,14]. Early accounts such as feature integration theory proposed that attention binds features at a single spatial locus [15], while binding-by-synchrony instead posits that neurons representing the same object fire in a temporally coordinated manner [16,17]. However, synchrony-based binding is challenged by the fact that biologically realistic heterogeneity in axonal delays tends to degrade precise synchrony, motivating alternative timing-based schemes.

At the same time, neurophysiology provides direct support for relational and temporal codes. Border-ownership cells in the early visual cortex (e.g., area V2) respond to an oriented edge while also signalling which side belongs to a figure, effectively encoding a binding relation between a local contour and an object-level assignment [18]. More generally, stimulus-specific information is often present in reliable spatiotemporal firing patterns, suggesting that precise spike timing can serve as a substrate for binding beyond rate codes [19,20]. These observations motivate mechanistic accounts in which binding is expressed through reproducible spike-time relationships, such as polychronous patterns that can operate with heterogeneous conduction delays [21].

### Ventral pathway feature hierarchy and boundary representations

Here we consider the primate visual cortex, a series of interconnected cortical areas organised hierarchically to process visual information at increasing levels of abstraction.

Within the primate ventral visual pathway, “hierarchy” is often described in terms of a systematic change in receptive-field scale and in the complexity of represented visual structure as signals progress from early to later areas. In early cortex (e.g., V1), neurons respond selectively to local oriented edges, while intermediate areas such as V4 contain neurons tuned to boundary curvature in an object-centred frame, and still later regions in inferotemporal cortex can integrate information over larger extents to support selectivity for more complex shape structure [8,10,11].

**Fig 1:**
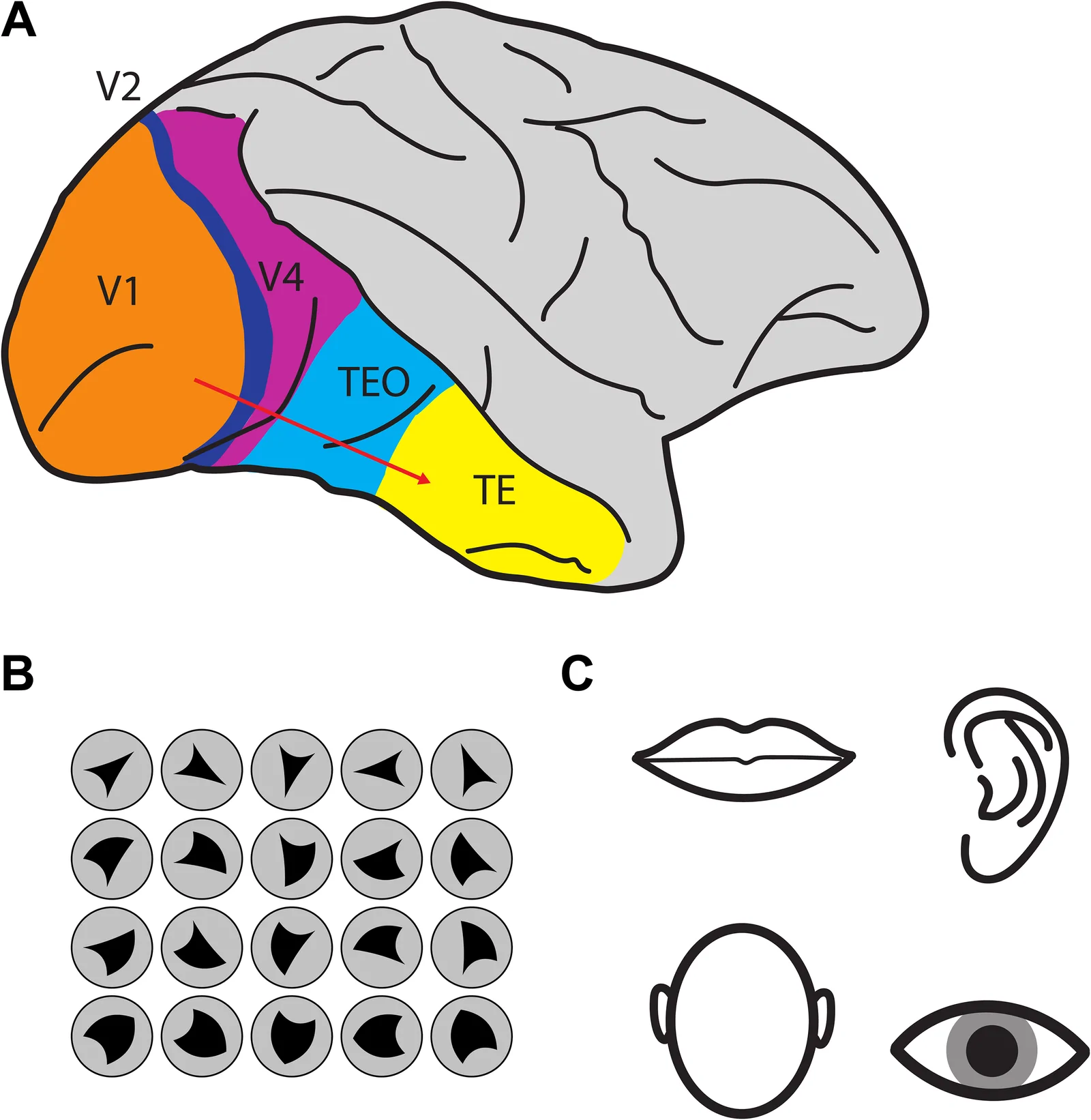
Cortical areas and boundary contour feature representations in the primate ventral visual pathway. **(A)** Cortical areas in the primate ventral visual pathway. As the pathway is ascended, neurons are selective for increasingly complex and larger scale shape features. **(B)** V4 neurons are selective to localised boundary contour elements in the frame of reference of an object. **(C)** Neurons in TEO and posterior TE encode larger scale combinations of localised boundary contour elements. Fig 1A adapted from a figure originally published in [23] under CC BY 4.0.

Visual signals in the primate cortex are often described as being processed through two distinct pathways: the dorsal stream, which rapidly processes motion and low spatial frequency signals, and the ventral stream, which processes colour and high spatial frequency signals at a slower speed. The latter is responsible for object recognition and representation of form [22] and is therefore the system of interest in this present study. The ventral visual pathway is depicted in Fig 1A with key cortical areas labelled.

In the initial stage of cortical processing within area V1, *simple cells* are selective for oriented bars and edges, complemented by *complex cells* that exhibit similar selectivity but additionally with a degree of translation invariance [24]. Following area V1, the visual signals proceed through areas V2, V4, TEO, and TE [25]. Ascending this pathway, the size of neuronal receptive fields increases, and neurons are selective for increasingly complex spatial features. In particular, lower cortical areas V1, V2, and V4 appear to process smaller scale visual features such as edges, contours, and colour, while the higher cortical areas TEO and TE are implicated in the processing of object shapes and faces. For example, in humans, the ventral temporal cortex includes the fusiform face area, which responds selectively to faces [26].

In particular relevance to this study are neurons found in area V4 that represent the boundary shapes of 2D objects, which encode the curvature of local boundary contour elements in an object-centred frame of reference [8,9]. Fig 1B illustrates the kind of localised boundary contour features that such a V4 neuron might respond to. Moreover, a population of such neurons can provide a distributed representation of the complete boundary shape of any 2D object. Ascending to the next areas of this visual pathway, namely TEO and posterior TE, neurons appear to integrate information from multiple V4 neurons in order to represent combinations of local boundary contour elements [10]. Fig 1C illustrates a combination of localised boundary contour features that a neuron in TEO or posterior TE might respond to. Finally, neurons at the end of the ventral visual pathway that encode whole objects may learn to respond to particular distributed representations of object boundary shape in earlier areas [27].

### Related modelling work

In a previously published modelling study [7], it was demonstrated how a rate-coded multi-layer neural network model of the ventral visual pathway could self-organise and develop neurons that encode the boundary contour elements of shapes in a manner similar to that observed in the visual brain [8–10]. That is, the neural network model developed a hierarchical representation of boundary contour elements at increasing spatial scales through successive layers of the network.

To achieve this, the authors trained their model on a wide collection of distinct 2D object shapes. For most of these tasks, the network was trained on a set of 2D shapes formed from unique combinations of boundary contour elements: each shape consisted of *n* sides, and each side assumed one of *p* possible conformations. For example, a set of shapes described by *n* = 4 and *p* = 2 consisted of four-sided shapes, where each side would be either concave or convex. The image sets devised in [7] included all possible shape configurations, giving a total of $p^{n}$ images and p×n distinct boundary contour elements. Trained on such images, the synaptic weights within the network self-organised through a process of unsupervised competitive learning.

Eguchi et al. [7] hypothesised that if the network model was trained on such a large number of different shapes, any particular boundary contour element, such as a right-side convexity, would only be seen with another particular boundary contour element, such as a bottom-side concavity, across a relatively small proportion of the full set of training images. This effectively creates a *statistical decoupling* between any two such boundary elements, which makes it less likely that a neuron in the network will learn to represent the combination of the two boundary contour elements. Moreover, as the total number of object shapes on which the network is trained increases, the total number of object shapes, $p^{n}$, grows much larger than the total number of boundary contour elements, p×n, used to construct the objects. In this case, the capacity limit of a competitive neural network forces individual neurons to switch from representing whole object shapes to instead representing the boundary elements. In this situation, it was postulated that the unsupervised competitive learning mechanism operating within the network during training would force some neurons in the higher layers of the network to learn to represent particular boundary contour elements rather than the whole object shapes, which was indeed observed from the simulations. Moreover, the network model developed a hierarchy of neurons encoding boundary contour elements at increasing spatial scales through successive neuronal layers, as observed in the brain.

Despite this reproduction of key aspects of ventral-stream shape processing, these simulations were carried out within a rate-coded neural network rather than an SNN. As such, the study did not incorporate temporally precise spiking dynamics, and it could not directly examine spike-timing-based binding mechanisms or the emergence of binding-neuron motifs that might encode hierarchical binding relationships between boundary contour elements across spatial scales.

### Hierarchical binding by polychronization

Polychronization is a phenomenon observed in SNNs, characterised by groups of neurons firing in time-locked but asynchronous spatiotemporal patterns which recur [21]. These neuron assemblies, known as polychronous neuronal groups (PNGs), exhibit precise timing relationships despite the lack of simultaneous activation. This capability suggests a mechanism for encoding information, and offers a potential substrate for hierarchical feature binding relationships via coordinated activity in multi-layer SNNs.

In addressing the hierarchical feature binding problem, the authors of [1,2] implemented a biologically realistic SNN model of the primate ventral visual pathway, characterised by several key properties:

- Their model explicitly simulated the precise timings of individual spikes fired by biologically-realistic neurons.
- The synaptic weights between pairs of excitatory neurons were adapted during visually-guided learning in an unsupervised manner, dependent on the relative timing differences between emitted pre- and postsynaptic spikes: a phenomenon known as spike timing dependent plasticity (STDP). Long-term potentiation (LTP) of a synapse occurred in response to pre- before postsynaptic firing, and conversely long-term depression (LTD) for post- before presynaptic firing [28,29].
- The network architecture was hierarchical, consisting of multiple, spatially-organised layers of neurons that included bottom-up, top-down and lateral synaptic connections. This kind of architecture is consistent with what is observed in the primate visual cortex.
- The network model incorporated axonal conduction delays in the synaptic connections between neurons, which governed the amount of time taken for a spike to propagate from its presynaptic origin to its postsynaptic target. The conduction delays between connected excitatory neurons were randomised at initialisation to values on the order of milliseconds to tens of milliseconds, consistent with measurements of biological networks [21].

Driven by STDP, and presented with Poisson-encoded images, the SNN self-organised to produce higher-layer PNGs. These emergent patterns were interpreted as representing hierarchical binding relationships, linking visual features across multiple spatial scales.

These prior studies were intentionally positioned as a proof-of-principle. Their stimulus sets consisted of three shapes, and the analysis emphasised demonstrating that polychronous structure and binding-neuron motifs can emerge in a biologically grounded hierarchical SNN [1,2]. In addition, much of the quantitative representational analysis in [1] focused on spike-pair PNGs. As noted in [2], apparent spike-pair structure can in some cases be difficult to disambiguate from elevated firing rates, motivating circuit-level criteria for identifying binding relationships.

The authors of [1,2] hypothesised that training a spiking neural network with the above properties on a simple composite shape (e.g., the letter ‘T’) would lead to the formation of a PNG in the upper layers that responds selectively to that shape. Within such a PNG, they proposed that one or more *binding neurons* would emerge whose spiking indicates whether specific lower-level feature neurons contribute to activating higher-level feature neurons. Concretely, a binding neuron would fire as part of the PNG if and only if activity representing a constituent element (e.g., a vertical bar) is participating in driving activity representing the composite feature (e.g., the ‘T’), thereby signalling that this lower-level feature is part of the higher-level one.

**Fig 2:**
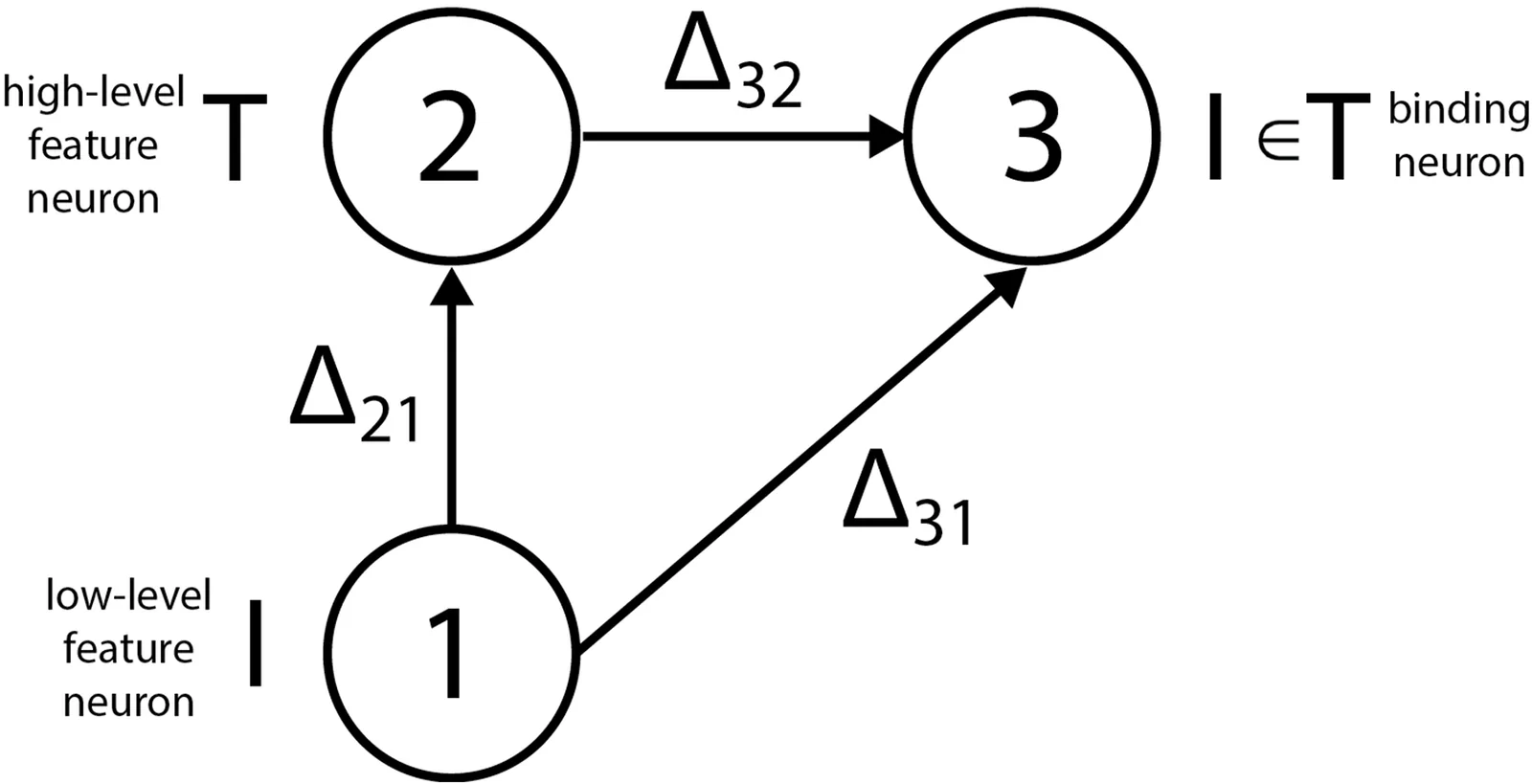
Simple theoretical binding circuit. This three-neuron hierarchical feature binding (HFB) circuit is the simplest kind of circuit posited to exist in the theory of hierarchical feature binding by polychronization [1,2]. Neuron 1 represents a low-level shape feature such as a vertical bar, neuron 2 represents a higher-level shape feature such as the letter ‘T’, and neuron 3 is a ‘binding neuron’ that represents whether the vertical bar is part of the ‘T’. Binding neuron 3 thus encodes the hierarchical binding relationship between the two features represented by neurons 1 and 2. The binding neuron 3 derives its firing characteristics from the pattern of axonal conduction delays between the three neurons. If $\Delta_{31}\approx \Delta_{21}+\Delta_{32}$, then the spikes from neurons 1 and 2 will arrive at neuron 3 simultaneously and cause it to fire if and only if neuron 1 is participating in driving neuron 2. In this case, the activity of binding neuron 3 will encode that the lower-level feature represented by neuron 1 is part of the higher-level feature represented by neuron 2.

The simplest example of how such binding neurons might operate is shown in Fig 2. The hierarchical feature binding (HFB) circuit is comprised of three neurons: neuron 1 is located in a lower layer and represents a low-level feature (e.g., a vertical bar), neuron 2 is located in a higher layer and represents a high-level feature (e.g., the letter ‘T’), and neuron 3 is a binding neuron (which could be located in either of the two layers) that encodes the hierarchical binding relationship between these features. The connections between the three neurons have axonal conduction delays $\Delta_{ij}$, where *j* indexes the presynaptic neuron and *i* the postsynaptic neuron. Let us assume that the three axonal delays shown in Fig 2 satisfy


$$\begin{array}{c} \Delta_{31}\approx \Delta_{21}+\Delta_{32}\;, \end{array}$$ (1)

where $\Delta_{ij}$ is the time taken for a spike fired by neuron *j* to reach neuron *i*. In this case, the spikes fired by neurons 1 and 2 will arrive at the binding neuron 3 simultaneously if and only if neuron 1 (representing the low-level feature) is participating in driving neuron 2 (representing the high-level feature). If the neurons have fast synaptic time constants, then binding neuron 3 will fire if and only if the incoming spikes from neurons 1 and 2 arrive simultaneously. The result of all this is that the binding neuron 3 will fire if and only if the low-level feature encoded by neuron 1 is actually part of the high-level feature or object encoded by neuron 2. In this way, the binding neuron 3 comes to encode information about the HFB relationship between the lower-level and higher-level visual features.

It was found that three-neuron binding PNGs of the general form shown in Fig 2 developed naturally in the higher layers of their SNN model when the network was trained on images of objects using STDP to adapt the strengths of synaptic connections between excitatory neurons [1,2].

The present study addresses the main gaps highlighted by [1,2] without expanding the stimulus scope to translation invariance or multi-object scenes. We perform a systematic quantitative analysis of three-neuron HFB circuits as the unit of binding, and examine their emergence across all network layers. Crucially, we test these mechanisms under a larger and more structured boundary-shape ensemble, enabling us to measure how HFB-structured PNGs relate to the hierarchy of learned feature selectivity and to quantify their prevalence and layer-wise organisation.

### Study questions

Specific questions we investigate through our simulations include the following:

1. Does the network model develop a hierarchy of feature neurons, through unsupervised competitive learning, which respond selectively to boundary contour elements of increasing spatial scale as seen in neurophysiology studies?
2. Do groups of neurons emerge in the higher network layers that exhibit the property of polychronization - i.e., emit their spikes in regularly repeating spatiotemporal patterns?
3. Do these neurons form HFB circuits, of the form depicted in Fig 2, which encode the hierarchical binding relationships between boundary contour elements at different spatial scales across the network hierarchy?

## Materials and methods

### Hierarchical network architecture

**Fig 3:**
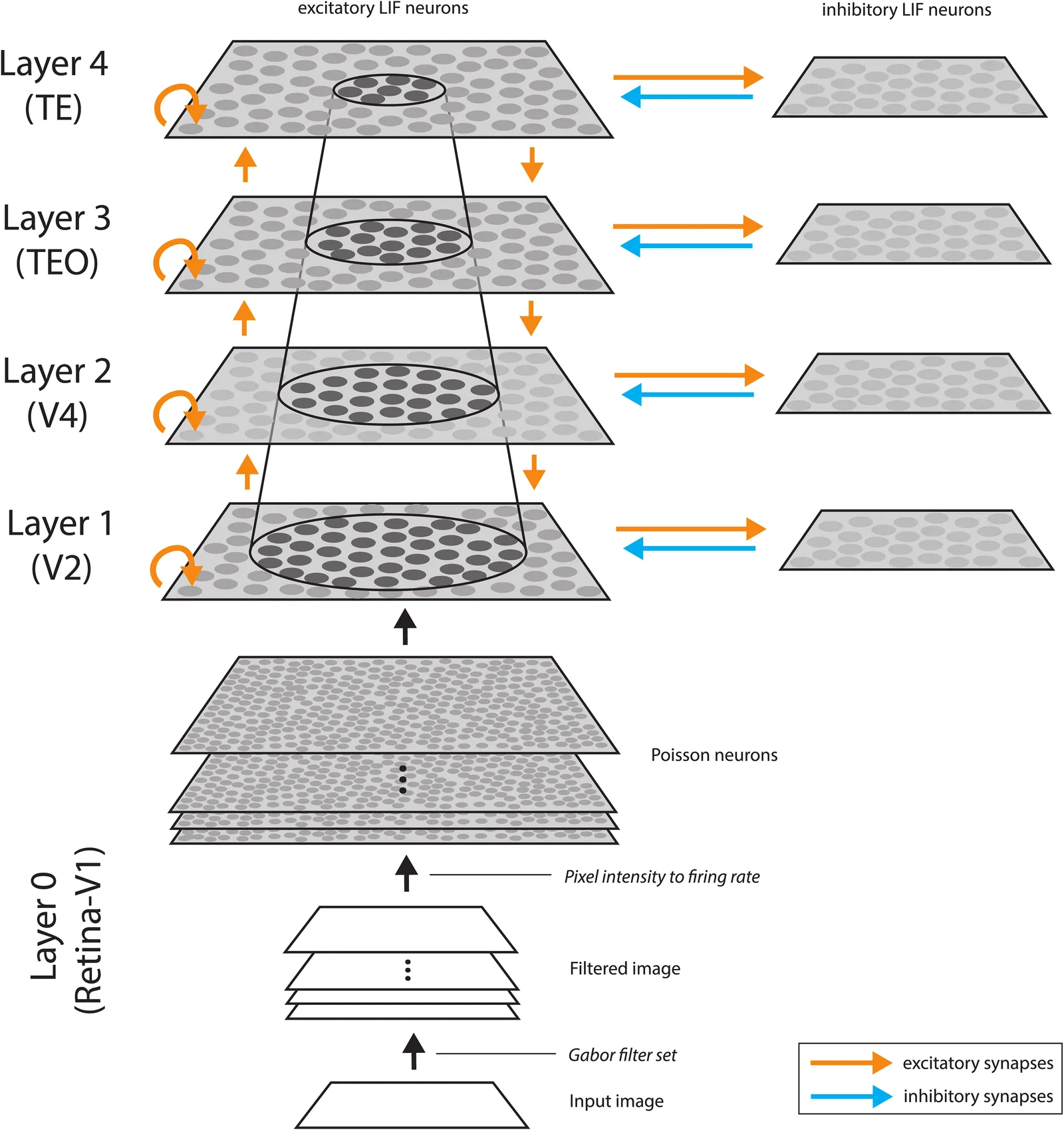
Architecture of the SNN model of the primate ventral visual pathway. Processing begins with input images being convolved with a set of Gabor filters that mimic the responses of bar and edge detecting simple cells in cortical area V1. The outputs of these filters are then used to determine the firing rates of retinal Poisson neurons in layer L0, where the individual spike timings are randomly generated according to a Poisson distribution. This input layer feeds into a hierarchical structure comprising four layers of spiking neurons (layers L1 to L4), each containing distinct populations of excitatory and inhibitory neurons. There are bottom-up (feedforward) and top-down (feedback) excitatory synaptic connections between adjacent layers, lateral (recurrent) connections within layers among excitatory neurons, and bidirectional connections between excitatory and inhibitory neurons within the same layer. The feedforward and feedback connections between excitatory neurons in adjacent layers are topologically organised. That is, excitatory neurons receive connections only from topologically corresponding local regions of the adjacent layers. Lateral synaptic connections between excitatory neurons and between excitatory and inhibitory neurons within the same layer are also formed in localised regions. This topological organisation facilitates a scalable receptive field across the hierarchy of layers, with neurons in higher layers integrating inputs over increasingly larger spatial extents, depicted as neurons shaded in dark grey.

The hierarchical SNN model explored in this study is depicted in Fig 3, and is designed to simulate the successive stages of visual information processing in the primate ventral visual pathway. This architecture derives from previous SNN modelling studies of this pathway [1,2], including notable studies of VisNet [7,30]. Specifically, it models four ascending cortical layers: V2, V4, TEO and TE, each critical to the visual processing pathway.

Each of these layers consists of subpopulations of excitatory and inhibitory interneurons, interconnected through certain synaptic projections. In more detail, connections between excitatory neurons are modifiable, and between adjacent layers there exist bottom-up (feedforward) and top-down (feedback) connections. Within the same layer, lateral connections exist between excitatory neurons. These connections are topologically organised, such that excitatory neurons only receive connections from neurons in a locally corresponding region of the same or an adjacent layer, which is determined according to the extent of their receptive fields. In this context, the receptive field of a particular neuron is a localised circular region of the visual space that affects its firing. This circular region is defined by a fan-in radius applied for each layer between the neuron and the visual space, along with an associated connection probability.

This model implements lateral competition among excitatory neurons, which is mediated by the subpopulation of inhibitory interneurons. This competition encourages feature selectivity and information sparsification, mimicking the competitive dynamics observed in the primate cortex, with a biologically accurate ratio of excitatory to inhibitory neurons close to 4:1. This lateral competition is supported via bi-directional synaptic connections between excitatory and inhibitory neurons, which are non-modifiable and formed between neurons in close proximity with each other.

Taken together, these topological connections are designed to promote increasingly complex and abstract representations of visual stimuli as information ascends through the visual pathway: the fan-in radius of connections from the retina expands in successive layers, allowing for a broader integration of visual features at higher levels of processing.

With respect to all network layers the feedforward, feedback and lateral connections between excitatory neurons incorporate axonal conduction delays with values on the time-scale of milliseconds, within biologically realistic constraints. The conduction delay of a synaptic connection refers to the time taken for an emitted spike to propagate from a presynaptic neuron and reach its postsynaptic target. Excitatory-excitatory connections are assigned conduction delays with random values selected from a uniform distribution, ranging from 1–10 ms, whereas connections associated with inhibitory neurons are fast and assigned a conduction delay of 0.1 ms. Introducing randomised conduction delays between excitatory neurons is essential to support the emergence of polychronization, and by extension feature binding relationships in the network. This is because these randomised delays provide a sufficient variety of initial neuronal structures for presented visual stimuli to activate and subsequently self-organise as some circuits are reinforced through STDP.

For detailed numerical parameters, including the numbers of excitatory and inhibitory neurons, the specific fan-in radii, associated connection statistics with respect to each layer, axonal conduction delays, and other topological characteristics of the synaptic connections, refer to Table 1.

**Table 1:** Model parameter choices. Terms are abbreviated as follows: FF = feedforward, FB = feedback, LAT = lateral, STDP = spike-timing-dependent plasticity. Multiple parameter choices inside curly braces are selected according to the run experiment, and specified in the results.

Parameters	Symbol	Layer			
		1	2	3	4
*(a) Network parameters*					
Number of excit. neurons	–	64×64	64×64	64×64	64×64
Number of inhib. neurons	–	32×32	32×32	32×32	32×32
Average number of FF afferent excit. connections per excit. neuron	–	50	100	100	100
Fan-in radius for FF afferent excit. connections to each excit. neuron	–	2	8	12	16
Average number of FB afferent excit. connections per excit. neuron	–	{0, 10}	{0, 10}	{0, 10}	–
Fan-in radius for FB afferent excit. connections to each excit. neuron	–	8	8	8	–
Average number of LAT afferent excit. connections per excit. neuron	–	{0, 10}	{0, 10}	{0, 10}	{0, 10}
Fan-in radius for LAT afferent excit. connections to each excit. neuron	–	2	2	2	2
Average number of LAT afferent excit. connections per inhib. neuron	–	10	10	10	10
Fan-in radius for LAT afferent excit. connections to each inhib. neuron	–	2	2	2	2
Average number of LAT afferent inhib. connections per excit. neuron	–	30	30	30	30
Fan-in radius for LAT afferent inhib. connections to each excit. neuron	–	4	4	4	4
*(b) Gabor filter parameters*					
Phase shift	ϕ	−π/2,π/2			
Wavelength	λ	0.8			
Orientation	θ	0,π/4,π/2,3π/4			
Spatial bandwidth	*b*	1			
Aspect ratio	γ	0.5			
Kernel size (pixels)	*k*	6			
*(c) Cellular parameters (excit. and inhib., respectively)*					
Membrane capacitance	$C_{m}$	500 pF, 214 pF			
Leakage conductance	$g_{L}$	25 nS, 18 nS			
Membrane time constant	$\tau_{m}$	20 ms, 12 ms			
Resting potential	*V*_0_	-74 mV, -82 mV			
Reversal potential	$\hat{V}$	0 mV, -70 mV			
Adaptation reversal potential for excit. cells	${\hat{V}}_{a}$	-90 mV			
Firing threshold potential	Θ	-53 mV, -53 mV			
After-spike hyperpolarization potential	$V_{h}$	-57 mV, -58 mV			
Absolute refractory period	$\tau_{R}$	2 ms			
Poisson neuron firing rate scaling factor	β	6 ms^–1^			
*(d) Synaptic parameters*					
Pre- and postsynaptic activity trace scaling factors	$\alpha_{C},\alpha_{D}$	0.5			
Pre- and postsynaptic STDP time constants	$\tau_{C},\tau_{D}$	5 ms			
Learning rate	ρ	{0.02, 0.04}			
Maximum excit. and inhib. axonal conduction delays	–	10 ms, 0.1 ms			
Conductance scaling factor for FF connections	$\lambda^{EfE}$	30 nS			
Conductance scaling factor for FB connections	$\lambda^{EbE}$	20 nS			
Conductance scaling factor for LAT excit. to excit. connections	$\lambda^{ElE}$	20 nS			
Conductance scaling factor for LAT excit. to inhib. connections	$\lambda^{ElI}$	30 nS			
Conductance scaling factor for LAT inhib. to excit. connections	$\lambda^{IlE}$	20 nS			
Conductance scaling factor for adaptation in exc. cells	$\lambda^{a}$	6 nS			
Synaptic time constant for all excit. to excit. connections	$\tau_{g}^{\left\{EfE,EbE,ElE\right\}}$	2 ms			
Synaptic time constant for LAT excit. to inhib. connections	$\tau_{g}^{ElI}$	2 ms			
Synaptic time constant for LAT inhib. to excit. connections	$\tau_{g}^{IlE}$	5 ms			
Synaptic time constant for adaptation in exc. cells	$\tau_{a}$	80 ms			
*(e) Simulation parameters*					
Exposure time per image (training)	–	100 ms			
Exposure time per image (inference)	–	250 ms			
Numerical time step	Δt	0.1 ms			

### Preprocessing visual stimuli by Gabor filtering

Prior to the network processing visual stimuli, images are first transformed using Gabor filters to simulate the tuning profiles of simple cells in the primary visual cortex (V1). The filter parameters are selected to align with the firing properties of V1 cells, aimed at extracting localised orientations of shape boundaries within the image space [31,32]. This approach ensures a distinct pattern of filter outputs for each of the visual objects, providing a suitable basis for generating input spikes by Poisson neurons in the network’s initial, retinal-V1 layer. Specifically, the filters are defined by the following set of equations:


$$\begin{array}{cc} g(x,y,\lambda ,\theta ,\phi ,b,\gamma ) & =\exp\left(-\frac{x^{\prime \;2}+\gamma^{2}y^{\prime \;2}}{2\sigma^{2}}\right)\cos\left(2\pi \frac{x^{\prime }}{\lambda }+\phi \right)\\ x^{\prime } & =x\cos\theta +y\sin\theta \\ y^{\prime } & =-x\sin\theta +y\cos\theta \\ \sigma & =\frac{\lambda \left(2^{b}+1\right)}{\pi \left(2^{b}-1\right)}\sqrt{\frac{\ln2}{2}}\;, \end{array}$$ (2)

where x and y specify the position of a light impulse in the visual field [33]. The other parameters include λ as the wavelength, θ the feature orientation, ϕ the phase, b the spatial bandwidth in octaves and γ the aspect ratio. The values of λ,θ,ϕ,b,γ as well as the kernel size *k* used to construct the Gabor filters are given in Table 1. The total number of Gabor filters implemented within the SNN architecture is determined by the number of distinct parameter combinations used to construct the filters. Additionally, to balance the filter outputs and to assign them equal importance, these filters have their mean set to zero and their Euclidean norm set to one.

Using these constructed Gabor filters, a grayscale image is preprocessed and converted into its filtered form as follows [34]:

1. The image is resized to a target resolution of 128×128 pixels, matched to the spatial dimensions of the network’s initial layer. The image is also standardised by dividing it by its standard deviation.
2. These Gabor filters are independently convolved with the image in two dimensions, maintaining the original image size. This convolution produces a retinotopic 2D map reflecting filter output values across the image, where the different filter types are vertically stacked as separate channels.
3. Each channel is rescaled by dividing its filter outputs with their Euclidean norm, thus ensuring a balanced input representation.

Following the above steps, these filter outputs are used to determine the firing rates of Poisson neurons constituting the network’s initial layer L0, with a direct one-to-one correspondence between each neuron and filter output. Hence, the input firing rates have a dimensionality of ${\mathbb{R}}^{W\times H\times C}$, where *W* and *H* refer to the spatial dimensions of the input space, being 128×128, and *C* the number of filter channels. Specifically, Poisson spike trains are generated with the probability of a spike event occurring over a small interval [t,t+Δt] given by:


$$\begin{array}{c} P\left\{input(x,y,f)\;spikes\;in\;\left[t,t+\Delta t\right]\right\}=\beta \cdot g(x,y,f)\cdot \Delta t\;, \end{array}$$ (3)

where *f* indexes the filter channel, β is a scaling factor which is tuned to constrain the input firing rate to less than 100 Hz, and Δt≪1 ms is a small time step. These Poisson neurons have an excitatory action, and project to the subpopulation of excitatory neurons residing in layer L1 in a topologically corresponding manner (Fig 3): each excitatory neuron in L1 receives its input from a spatially aligned, localised region of Poisson neurons, spanning all of the filter channels, with a fan-in radius and expected number of connections given by Table 1.

We hypothesise that training SNNs on randomised (Poisson) input spike timings will cause the synaptic connections in the higher layers to self-organise via STDP to produce visual representations that are highly robust with respect to image noise. The randomness introduced at the input layer will force the network to learn patterns without relying on the precise timing of individual spikes. This could lead to the emergence of synaptic connections that are more invariant to noise and perturbations.

### Differential equations

#### Leaky-integrate-and-fire model.

Following related SNN modelling studies [1,2], neurons in all layers of our network except V1 are modelled as conductance-based leaky-integrate-and-fire (LIF) neurons [35]. According to this model, the membrane potential $V_{i}$ of a neuron *i* at time *t* is updated as follows:


$$\begin{array}{c} \tau_{m}^{\gamma }\frac{dV_{i}(t)}{dt}=V_{0}^{\gamma }-V_{i}(t)+R^{\gamma }I_{i}(t)\;, \end{array}$$ (4)

where $V_{i}$ is driven by synaptic current $I_{i}$ integrated over the neuron’s afferent synapses; the membrane potential is driven upwards in response to current from excitatory synaptic conductances and downwards with respect to inhibitory synaptic conductances. The membrane properties of the neuron determine its decay back towards its resting state, described by its resting potential *V*_0_. The membrane time constant is defined by $\tau_{m}=C_{m}/g_{0}$, where $C_{m}$ and *g*_0_ refer to the membrane capacitance and leakage conductance, respectively. The neuron’s membrane resistance is the inverse of the leakage conductance, given by *R* = 1 / *g*_0_. The neuron belongs to one of two classes: excitatory or inhibitory, with class-specific parameter values indexed by the superscript γ. Concerning spike generation, the neuron fires a spike at time $t_{i}^{f}$ when its membrane potential crosses the firin*g* threshold Θ from below. Immediately after the neuron fires, its membrane potential is reset to its after-spike hyperpolarisation potential $V_{h}$ where it is held for an absolute refractory period $\tau_{R}$.

The total synaptic current entering a neuron is calculated as the sum of all afferent synaptic conductances, both excitatory and inhibitory, multiplied by the potential difference between the reversal potential specific to the synapse class, ${\hat{V}}^{\gamma }$, and the membrane potential of the neuron, $V_{i}$. The time-dependent conductance for a particular synapse is denoted by $g_{ij}$ with *j* and *i* indexing the pre- and postsynaptic neurons, respectively:


$$\begin{array}{c} I_{i}(t)=\sum_{\gamma }\sum_{j}g_{ij}(t)({\hat{V}}^{\gamma }-V_{i}(t))\;. \end{array}$$ (5)

Additionally, for an excitatory postsynaptic neuron *i*, the total synaptic current is adjusted to include an adaptation conductance, $g_{i}^{a}$, with an associated reversal potential ${\hat{V}}^{a}$ as follows:


$$\begin{array}{c} I_{i}(t)\leftarrow I_{i}(t)+g_{i}^{a}(t)({\hat{V}}^{a}-V_{i}(t))\;. \end{array}$$ (6)

This homeostatic mechanism is necessary to regulate the firing activity of excitatory neurons, preventing excessively high firing rates from emerging during network training [36].

#### Synaptic conductance equations.

The conductance of a specific synapse, $g_{ij}$, is determined by two components: a decay term $\tau_{g}$, and a change induced by a Dirac delta function when spikes arrive from a presynaptic neuron *j*. This relationship is expressed as follows:


$$\begin{array}{c} \frac{dg_{ij}(t)}{dt}=-\frac{g_{ij}(t)}{\tau_{g}}+\lambda \Delta g_{ij}(t)\sum_{l}\delta (t-\Delta t_{ij}-t_{j}^{l})\;, \end{array}$$ (7)

where the axonal conduction delay associated with a particular synapse is denoted by $\Delta t_{ij}$, and the received presynaptic spikes are indexed by *l*. The conduction delays are initia*l*ised by either selecting random, uniformly distributed values ranging between 1–10 ms for excitatory synapses, or being set to 0.1 ms for inhibitory synapses. The parameter λ is a biological scaling constant, and is used to scale the synaptic efficacy (or weight) $\Delta g_{ij}$ which lies between unity and zero. The Dirac delta function captures the instantaneous change in $g_{ij}$ at the time of presynaptic spike arrival, and is defined as follows:


$$\begin{array}{c} \delta (x)=\left\{ \begin{array}{cc} \infty & \text{if }x=0\\ 0 & \text{otherwise} \end{array}\right.\;\text{where}\;\int_{-\infty }^{\infty }\delta (x)\;dx=1\;. \end{array}$$ (8)

Similarly, the dynamics of the adaptation conductance, $g_{i}^{a}$, for an excitatory postsynaptic neuron *i* is determined as follows:


$$\begin{array}{c} \frac{dg_{i}^{a}(t)}{dt}=-\frac{g_{i}^{a}(t)}{\tau_{a}}+\lambda^{a}\sum_{k}\delta (t-t_{i}^{k})\;, \end{array}$$ (9)

where the parameter $\lambda^{a}$ determines the size of the instantaneous change in $g_{i}^{a}$ at the time of an emitted postsynaptic spike, which is indexed by *k*.

#### Synaptic learning equations.

The differential equations outlined below characterise STDP at modifiable excitatory-to-excitatory synapses, where plasticity is implemented across all bottom-up, top-down, and lateral connections among excitatory neurons spanning layers 1–4 [1,2]. These STDP equations are adapted from [37], and are described for a synaptic connection between pre- and postsynaptic neurons *j* and *i*, respectively, as follows.

The recent presynaptic activity is tracked by a variable, $C_{ij}$, and can be understood as the concentration of neurotransmitter (glutamate) released into the synaptic cleft [37]. This activity variable is constrained within the closed interval [0, 1] for $0\leq \alpha_{C}\leq 1$, and is governed by a decay term $\tau_{C}$ as follows:


$$\begin{array}{c} \frac{dC_{ij}(t)}{dt}=-\frac{C_{ij}(t)}{\tau_{C}}+\alpha_{C}(1-C_{ij}(t))\sum_{l}\delta (t-\Delta t_{ij}-t_{j}^{l})\;. \end{array}$$ (10)

This activity trace is driven up in response to the arrival of presynaptic spikes at the postsynaptic neuron, scaled by $\alpha_{C}$, where the conduction delay associated with the synapse is accounted for via $\Delta t_{ij}$; hence, $C_{ij}$ is only updated when presynaptic spikes actually arrive at the postsynaptic neuron, as opposed to when they are originally fired by the presynaptic neuron.

The postsynaptic neuron’s recent activity, tracked by the variable $D_{i}$, reflects the proportion of unblocked NMDA receptors due to recent depolarization from back-propagated action potentials [37]. The time evolution of this activity variable is controlled by a decay term $\tau_{D}$, and is modelled as follows:


$$\begin{array}{c} \frac{dD_{i}(t)}{dt}=-\frac{D_{i}(t)}{\tau_{D}}+\alpha_{D}(1-D_{i}(t))\sum_{k}\delta (t-t_{i}^{k})\;, \end{array}$$ (11)

where $D_{i}$ is driven up in response to the emission of postsynaptic spikes by an amount proportional to the parameter $\alpha_{D}$. Postsynaptic spike times are indexed by *k*.

The strength of the synaptic weight, $\Delta g_{ij}$, is modified according to captured values for $C_{ij}$ and $D_{i}$ as follows:


$$\begin{array}{cc} \tau_{\Delta_{g}}\frac{d\Delta g_{ij}(t)}{dt}=\rho [ & C_{ij}(t)\sum_{k}\delta (t-t_{i}^{k})-D_{i}(t)\sum_{l}\delta (t-\Delta t_{ij}-t_{j}^{l})]\;, \end{array}$$ (12)

where $\tau_{\Delta_{g}}$ is the time constant, and the learning rate, controlling the magnitude of a synaptic weight change, is denoted by ρ. The summation index *k* is over spikes emitted by the postsynaptic neuron *i*, and the summation index *l* is over spikes emitted by the presynaptic neuron *j*. For the rest of this paper, the learning rate is redefined as $\rho \leftarrow \rho /\tau_{\Delta_{g}}$, thus eliminating $\tau_{\Delta_{g}}$ from the above equation to more directly ref*l*ect the effective weight change arising from STDP.

The described STDP model functions in the following manner. The first term on the RHS of Eq (12) represents long-term potentiation (LTP): if the presynaptic activity variable $C_{ij}$ is elevated, indicative of recent presynaptic spikes at the postsynaptic neuron, and a postsynaptic spike occurs, synaptic potentiation is facilitated. This results in an increase in the synaptic weight. Conversely, the second term on the RHS of Eq (12) represents long-term depression (LTD): if the postsynaptic activity variable $D_{i}$ is elevated, reflecting recent postsynaptic spiking, and a presynaptic spike arrives at the postsynaptic neuron, synaptic depression occurs. This leads to a reduction in the synaptic weight. The parameter values used in this paper are specified in Table 1, and parameters with multiple choices, such as the learning rate ρ, are described in the results.

#### Numerical scheme.

The differential equations previously outlined are discretised into finite difference equations and solved using a fourth order Runge-Kutta (RK4) integration method. Simulations are conducted with a time step of Δt=0.1 ms, using the Brian2 simulator [38].

### Topological connectivity

Every neuron in the network is assigned a particular spatial location within its layer. The Poisson neurons in L0 are each assigned to a specific retinotopic location within the visual field. Each Poisson neuron then represents a particular form of local edge or bar feature at the corresponding retinotopic location within an image. Neurons in the higher layers 1–4 are also arranged in a corresponding retinotopic manner within their respective layers.

Individual excitatory neurons within layers 1–4 receive bottom-up and top-down connections from excitatory neurons within topologically corresponding localised regions of the adjacent layers as illustrated in Fig 3. Excitatory neurons in adjacent layers are connected with probability $p_{conn}$ (set per projection class to match the expected number of afferent connections in Table 1), if the condition specified by Eq (13) is met:


$$\begin{array}{c} {\left\|{\vec{x}}_{i}-{\vec{x}}_{j}\right\|}_{2}=\sqrt{{\left(x_{i}-x_{j}\right)}^{2}+{\left(y_{i}-y_{j}\right)}^{2}}<r_{\text{in}}\;, \end{array}$$ (13)

where ${\vec{x}}_{i}$ and ${\vec{x}}_{j}$ are respectively the spatial locations of the post- and presynaptic neurons, *x* is horizontal location, *y* is vertical location, and $r_{in}$ is the fan-in radius.

Implementing bottom-up connectivity with this kind of topological organisation allows the development of classical neuronal receptive fields that increase in size as the network layers are ascended. Synaptic connections within layers are formed using the same distance criterion (Eq (13)): each excitatory neuron receives lateral excitatory input from nearby excitatory neurons (E→E), and lateral inhibitory input from nearby inhibitory interneurons (I→E), while inhibitory interneurons receive local drive from nearby excitatory neurons (E→I). These lateral projections are sampled with the corresponding $p_{conn}$ within their fan-in radii, with expected connection counts per neuron specified in Table 1.

### Unsupervised competitive learning

The SNN model presented in this paper is trained using *unsupervised competitive learning*. In contrast to the kind of supervised learning that is typically used to train deep neural networks for machine vision tasks, the network is not supplied with any additional information about the presented visual stimuli, such as object shape labels, during training. In particular, we do not instruct the neurons in the final layer how they should respond to each of the shape images during training. Unsupervised competitive learning operates as follows:

- During training, each image is presented in turn to the network. During each image presentation, activity is allowed to flow through the synaptic connectivity within the network for several hundred milliseconds.
- Within each layer there are separate pools of excitatory and inhibitory neurons. There are bi-directional connections between these excitatory and inhibitory pools. The excitatory neurons excite the inhibitory neurons, while the inhibitory neurons depress the activity of the excitatory neurons.
- When a subpopulation of excitatory neurons within a layer becomes activated due to the visual input from an image presented to the network, they excite a subpopulation of inhibitory neurons in the same layer. These active inhibitory neurons then depress the activity of nearby excitatory neurons. In this way, the excitatory neurons effectively ‘compete’ with each other through lateral inhibition mediated by the pool of inhibitory neurons. The excitatory neurons that ‘win’ the competition and remain active then learn via STDP in the bottom-up connections to respond to the current active inputs from the preceding layer. This process occurs in every layer of the network, from the final layer to the input layer.
- In this way, excitatory neurons throughout the network learn to respond to specific visual features present within the images at different spatial scales. Neurons in the lower layers, which receive signals from smaller regions of retinal space and so have smaller receptive fields, will typically learn to respond to smaller, simpler visual features such as short local boundary contour elements. While neurons in higher layers, with larger receptive fields, will typically learn to respond to larger, more complex features such as longer boundary contour elements.

We demonstrate here that unsupervised competitive learning combined with STDP also leads to the emergence of ‘binding neurons’ that represent the HFB relationships between visual features at different spatial scales across successive layers. As stated, these outcomes occur without the need to specify how the neurons in the final layer should respond to each of the object shape images during training.

Unsupervised competitive learning within SNNs may produce quite different kinds of visual features than those that emerge within deep neural networks trained by supervised learning. Unsupervised competitive learning drives neurons within the network to learn to represent the *statistically significant* visual features that tend to reoccur regularly across images. We propose that such natural visual features may help to provide a basis set for the representation of objects that is more robust with respect to image noise and natural object transformations. In contrast, the features that emerge within deep neural networks may be more abstract and lead to arbitrary and unnatural decision boundaries.

### Training on visual stimuli and inference

**Fig 4:**
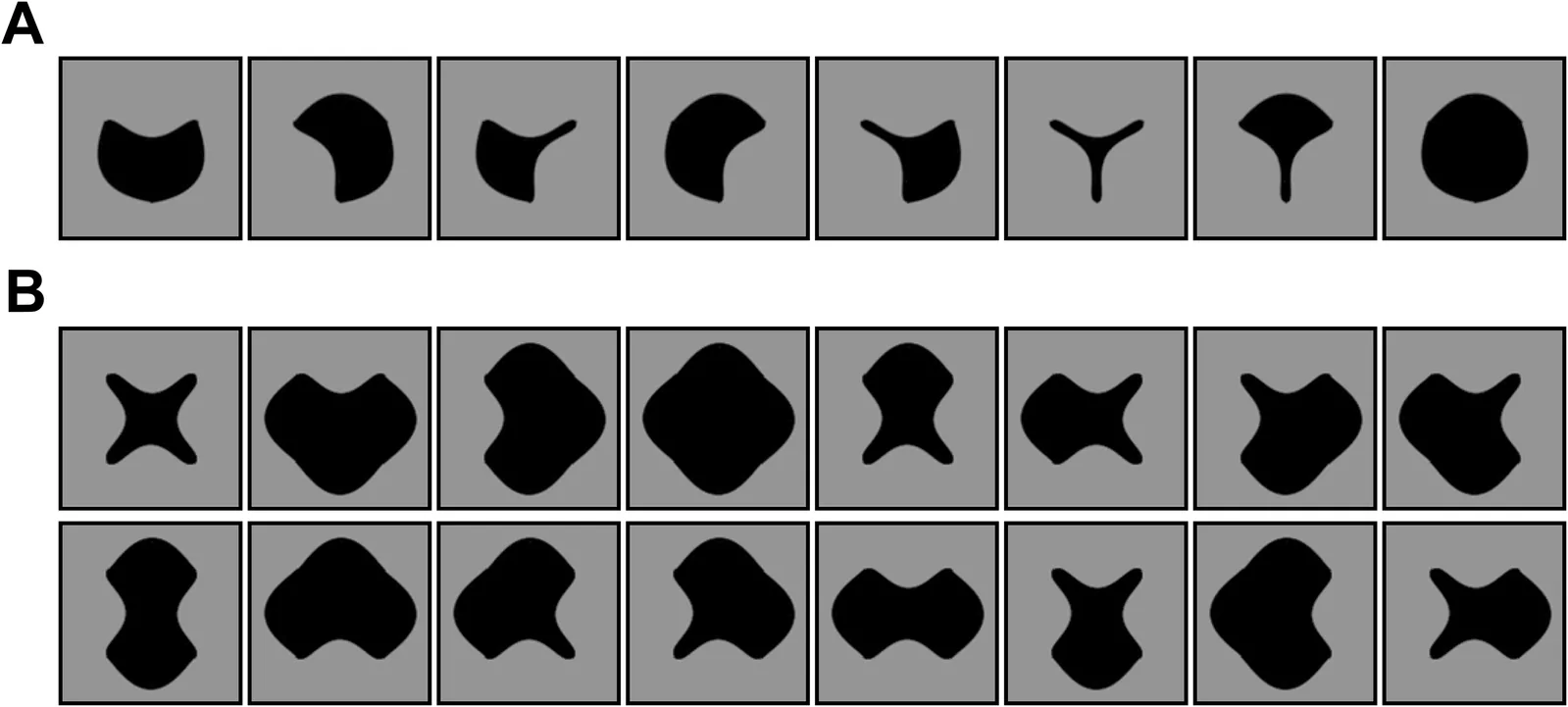
Sets of visual stimuli used for training, consisting of simple 2D object shapes formed from *n* sides and *p* conformations per side. (A) This reduced set, termed N3P2, consisted of shapes described by *n* = 3 and *p* = 2: totalling $p^{n}=8$ different shapes and n×p=6 distinct boundary contour elements. **(B)** This expanded set, N4P2, was described by *n* = 4 and *p* = 2: totalling 16 shapes and 8 boundary contour elements.

During training the network is exposed to an ecologically realistic set of similar 2D object shapes, where each shape is configured from a unique combination of boundary contour elements [7]. Specifically, a shape is described by *n* sides and each side assumes one of *p* possible conformations, giving rise to a total of $p^{n}$ different shapes and p×n unique boundary contour elements. This study only considers shapes with two conformations at each side (convex or concave), and sets of images containing either three- or four-sided shapes. Hence, the network is trained on a set of images containing either 8 or 16 shapes, referred to by the dataset names ‘N3P2’ and ‘N4P2’, respectively (Fig 4).

Because N3P2 and N4P2 enumerate all $p^{n}$ configurations with *p* = 2 (convex vs. concave per side), both datasets are exactly balanced. For each object side, convex and concave boundary elements occur equally often (50% each), and for any pair of sides all four joint configurations are equally represented. As a compact summary of feature diversity, the number of convex sides per shape follows the binomial counts: for N3P2 the counts for *k* = 0,1,2,3 convex sides are 1,3,3,1 (out of 8 shapes), and for N4P2 the counts for *k* = 0,1,2,3,4 are 1,4,6,4,1 (out of 16 shapes). These summaries clarify in what sense the datasets are “ecologically realistic” for our modelling objective: the same small set of boundary parts is reused across many distinct shapes, so each part is seen across multiple contexts. This is the condition required for the “statistical decoupling” argument, and it promotes the emergence of feature-based representations under competitive learning.

The network’s learning rate, ρ, is set to 0.04 and 0.02 when trained on N3P2 and N4P2, respectively. The set of images for a dataset are presented to the network as separate epochs, where one training epoch corresponds to the presentation of each one of these images. The duration of an image presentation is 100 ms, and the total number of training epochs is 60, which was established from preliminary simulation runs to result in a convergent network state; this is equivalent to 48 s and 96 s of total simulation time with respect to the N3P2 and N4P2 datasets, respectively.

The modifiable connections in the network which are adapted via STDP during training include feedforward (FF), feedback (FB) and excitatory-to-excitatory lateral (LAT) connections. Non-modifiable connections which remain fixed during training include the connections between excitatory and inhibitory neurons. The synaptic weights $\Delta g_{ij}$ of modifiable connections are initialised by independently selecting values from a uniform distribution in the range [0, 1], while non-modifiable connections are initialised with synaptic weight values that are all set to unity.

Concerning inference, after training STDP is disabled and the spiking activity of neurons across all layers is recorded in response to the same training images being presented to the network. Each image is presented to the network for a duration of 250 ms, and this is repeated a certain number of times to draw statistically meaningful results. Additionally, and unless specified otherwise, the first 50 ms of each recording is discarded to account for the spillover in network responses between successive image presentations, thereby allowing it time to settle into each new activity state.

### Performance measures

#### Single cell information analysis.

Information theory provides a framework to quantify the amount of information that individual neurons convey regarding specific types of visual stimuli through their firing rate responses, which can be used as a measure to assess how selectively neurons respond to their preferred stimuli. In this context, a specific type of stimulus refers to a set of object shapes sharing a common characteristic, such as a left-side convexity versus a left-side concavity. For example, all shapes with a left-side convexity can be considered as variations or “transforms” with respect to this characteristic, despite differences in the other visual features. Accordingly, the set of shapes sharing this feature represents the distinct forms, or transforms, of a specific visual stimulus, denoted as *s* [7].

To evaluate the amount of information each neuron carries about these distinct stimulus types, we compute an information-theoretic measure based on the firing rate responses recorded during network inference. From analysing the distribution of these responses with respect to each type of stimulus, the stimulus-specific information conveyed by a neuron can be calculated using the following equation [39]:


$$\begin{array}{c} ℐ(s,\vec{R})=\sum_{r\in \vec{R}}P(r|s)\log_{2}\frac{P(r|s)}{P(r)}\;, \end{array}$$ (14)

where *s* is a specific stimulus, *r* is the neuronal response to *s*, and $\vec{R}$ is the set of neuronal responses across the entire set of stimuli. Here, *P*(*r*|*s*) is estimated empirically from the distribution of firing-rate responses over repeated presentations of shapes belonging to class *s*, and *P*(*r*) is the corresponding marginal across all stimulus classes.

The maximum amount of information that a neuron can impart about *n* distinct types of stimuli is given by $\log_{2}(n)$ bits, signifying an unambiguous response profile. This study considers two categories of visual stimuli at a given object side: convex vs. concave conformations. We therefore compute stimulus-specific information separately for each side, for which the maximum is one bit. These calculations are performed for each excitatory neuron in the network’s final layer (L4) and for each object side.

To summarise selectivity in a manner that is independent of side location, we additionally define each neuron’s *preferred side* as the one yielding its maximum stimulus-specific information. When reporting convex-selective responses, this maximum value is retained only if the neuron’s mean firing rate is higher for convex than concave at its preferred side (otherwise the convex-selective information is set to zero); an analogous criterion is used when reporting concave selectivity by swapping the stimulus labels. This procedure allows us to identify and rank neurons that are most informative about the presented stimuli, either at a fixed side or using a pooled (max-over-sides) summary, depending on the analysis.

#### Traceback analysis.

Following the identification of neurons that are particularly informative and selectively responsive to distinct types of visual stimuli, a technique termed ‘traceback analysis’ is applied to locate the regions within the retinal input space (layer L0) to which these neurons are most sensitive. By examining these regions through visual inspection, the specific object shape features that are primarily responsible for eliciting the neuronal responses can be identified. This analysis is used to confirm that trained neurons become tuned to specific boundary contour elements and provides a mechanistic explanation for their observed firing rate characteristics.

The procedure for this method is as follows. We start by considering an excitatory neuron indexed by *i* that resides in the final layer *l* = *L*, where *L* > 0. Hence, the neuron’s activation is defined as


$$\begin{array}{c} a_{i}^{L}=\sigma (net_{i}^{L})\;, \end{array}$$ (15)

where σ is an activation function, and $net_{i}^{L}$ is the neuron’s weighted input. For tractability, we approximate a neuron’s activation as arising solely from feedforward excitatory connections, such that


$$\begin{array}{c} net_{i}^{L}=\sum_{j}w_{ij}^{L}a_{j}^{L-1}\;, \end{array}$$ (16)

where the summation runs over all presynaptic excitatory neurons indexed by *j* in layer L−1 that project to the postsynaptic neuron *i*, and we assume $w_{ij}^{L}:=\Delta g_{ij}^{L}$. Accordingly, using Eqs (15) and (16), the sensitivity of the postsynaptic neuron’s activation to that of a selected Poisson neuron indexed *k* in the retinal layer (*l* = 0) can be expressed as follows:


$$\begin{array}{cc} \frac{\partial a_{i}^{L}}{\partial a_{k}^{0}} & =\frac{\partial \sigma (net_{i}^{L})}{\partial a_{k}^{0}}\\ & =\sigma^{\prime }(net_{i}^{L})\sum_{j}w_{ij}^{L}\frac{\partial a_{j}^{L-1}}{\partial a_{k}^{0}}\; \end{array}$$ (17)

To compute the above expression, we apply the technique of backpropagation, defining sensitivity terms with respect to the last and preceding layers as follows:


$$\begin{array}{cc} \delta_{i}^{L} & :=\sigma^{\prime }(net_{i}^{L})\\ \delta_{j}^{L-1} & :=w_{ij}^{L}\delta_{i}^{L}\cdot \sigma^{\prime }(net_{j}^{L-1})\;, \end{array}$$ (18)

and recursively for intermediate layers, 1<l≤L−2:


$$\begin{array}{c} \delta_{j}^{l}:=[\sum_{i}w_{ij}^{l+1}\delta_{i}^{l+1}]\sigma^{\prime }(net_{j}^{l})\;. \end{array}$$ (19)

Hence, using these definitions, it can be shown that the sensitivity of the neuron’s activation to an input Poisson neuron is determined by


$$\begin{array}{c} \frac{\partial a_{i}^{L}}{\partial a_{k}^{0}}=\sum_{i^{\prime }}w_{i^{\prime }k}^{1}\delta_{i^{\prime }}^{1}\;, \end{array}$$ (20)

where the index $i^{\prime }$ runs over excitatory neurons residing in the first intermediary layer (*l* = 1) that receive feedforward connections from the presynaptic Poisson neuron indexed by *k*.

In this analysis, we evaluate a neuron’s activation as its time-averaged firing rate over the duration of a presented object shape and assume an exponential dependence of a neuron’s activation on its weighted input, such that $\sigma (x)=e^{x}$. The sensitivity of a selected neuron is computed with respect to each Poisson neuron in the input layer, and the resulting values are averaged across the filter channels at each spatial location. The normalised sensitivities can then be plotted as a spatial heatmap, providing a visual representation of the neuron’s receptive field.

#### Polychronous neuronal group detection.

To identify functionally significant neuronal activity patterns and explore their roles in hierarchical information processing, we utilise the Spike PAttern Detection and Evaluation (SPADE) method [40]. SPADE employs techniques from frequent item set mining to detect reoccurring spatiotemporal spike patterns, or PNGs, from massively parallel spike train data, and has been optimised for this purpose [41]. This method also assesses the statistical significance of detected patterns by using surrogate data generated through spike-timing dithering, thereby isolating reliably reoccurring patterns. We use the SPADE implementation available as a submodule within the Python library Elephant (ELEctroPHysiological ANalysis Toolkit) [42,43].

This study specifically examines PNGs that correspond structurally to the three-neuron HFB circuit depicted in Fig 2, in which both the high-level feature neuron and the binding neuron reside in a layer immediately following that of the low-level feature neuron. This configuration serves as a fundamental unit for integrating hierarchical feature information across spatial scales, and is selected to keep the analysis tractable and the scope of this study focused. By applying SPADE, we systematically investigate the emergence of this HFB circuit within our SNN model. The workflow for performing detection with SPADE is as follows:

1. *Data preparation:* Inference recordings of spike trains from all excitatory neurons in the network are organised into a 4D array with dimensions corresponding to image ID, image repetition number, layer ID, and neuron ID. Each element in this array contains an associated, variable-length, neuronal spike train, as recorded for a particular image presentation. To ensure computational feasibility, only the first 10 image repetitions are retained for analysis.
2. *Data concatenation:* To manage the large volume of data, the spike trains are concatenated across the image repetition and image ID dimensions. This results in a 2D array where each element, indexed by layer ID and neuron ID, contains the concatenated spike train for that neuron across all considered images and their repetitions. A 50 ms gap is inserted between concatenated spike trains from different recordings to avoid misidentification of patterns that span separate trials. This approach preserves all spike timing information, ensuring no loss of data integrity.
3. *Iterative detection:* SPADE is applied independently with respect to each excitatory neuron across layers 1–4 by selecting each neuron as a target and analysing its immediate set of connected neurons. The target neuron is evaluated for its potential to represent a high-level feature as part of a three-neuron HFB circuit that conforms structurally to the one illustrated in Fig 2. The search space is constrained to the immediate set of excitatory neurons directly connected to the target neuron via feedforward or lateral connections, each with a synaptic weight of at least 0.5. This restriction ensures that only the specified three-neuron HFB circuit is considered, excluding any other circuit configurations. Additionally, only neurons with appropriate axonal conduction delays for participating in these circuits are included in the search. This process results in a refined collection of candidate spatiotemporal spike patterns.
4. *Significance testing:* The statistical significance and stability of these candidate patterns are assessed using SPADE. During this step, surrogate datasets are generated by applying spike-time dithering with a magnitude of 5 ms (100 surrogates), resulting in a p-value spectrum for the patterns. Candidate patterns are filtered based on a significance threshold of *p* < 0.05, ensuring that only robust patterns are retained for further analysis.

On each of these iterations, the SPADE method first converts spike trains into binary arrays, using a time precision at 1 ms resolution, before searching for reoccurring spatiotemporal spike patterns with a maximum span of 20 ms between the first and last spikes. Such a pattern must occur at least three times and involve three distinct neurons to be included for further analysis.

At the post-processing stage, these pattern detections are further refined based on their alignment with the desired configuration of an HFB circuit (c.f. Fig 2), according to the following criteria:

1. The neuron that fires first must be located in the layer immediately preceding the one containing the second-firing neuron, acting as the low-level feature neuron. Additionally, the second- and third-firing neurons must reside in the same layer, respectively functioning as the high-level and feature-binding neurons.
2. There must be feedforward connections from the first neuron to both the second and third neurons, as well as a lateral connection from the second to the third neuron.
3. The recorded spike-timing differences between connected neurons are assessed relative to their associated axonal conduction delays. Specifically, each spike-timing difference must fall within the range $\left[\Delta t_{ij},\Delta t_{ij}+\delta t\right]$, where $\Delta t_{ij}$ is the axonal delay and δt=3 ms is the tolerance.

After meeting these criteria, spatiotemporal spike patterns involving the same set of neurons that fire in the same order are merged into a unique, characteristic pattern. This merging process involves calculating the weighted average of spike-timing differences for each neuron pair across all corresponding patterns, with the weights determined by the frequency of occurrence of each pattern. Averaging spike-timing differences in this way deduplicates similar patterns, yielding a representative or characteristic spatiotemporal spike pattern, referred to as a PNG. This technique streamlines further analysis and helps to compensate for inherent variability in spike timing due to network noise.

#### PNG selectivity metrics.

Following the identification of PNGs using the SPADE method, defining appropriate selectivity metrics is crucial to assess their functional role in signalling the characteristics of visual stimuli. Given the increased complexity of three-neuron PNGs, applying an information theoretic measure, as used previously in simpler spike-pair configurations [1], is inadequate. In the context of this study, such a measure might fail to capture the more intricate dynamics and multiple pairwise associations between neurons encoding an HFB circuit.

Instead, this study employs *precision*, *recall* and their *F1 score* as the primary metrics for evaluating the information carried by PNGs about specific boundary conformations, such as convex vs. concave. These metrics are computed separately for each side of the object shapes. Specifically, for each side, there are two stimulus categories: one defined as the set of object shapes with a convex boundary element at that side, and the other as the set with a concave boundary element at that side. We explore whether some PNGs have learned to respond selectively to one of these stimulus categories but not the other.

Similarly as described previously in the section *Single cell information analysis*, object shapes are thus split into these two categories for each side according to a shared spatial feature, where each category corresponds to all transforms of a specific visual stimulus. Hence, if we define the preferred visual stimulus as *s*, we consider a PNG that activates at least once in response to an object shape belonging to *s* to be a ‘true positive’. By extension, if a PNG activates in response to an object shape that doesn’t belong to *s* then this is treated as a ‘false positive’. The precision of a PNG with respect to *s* is then defined in terms of these two outcomes:


$$\begin{array}{c} Precision=\frac{TP}{TP+FP}\;, \end{array}$$ (21)

where *TP* is the number of true positives and *FP* is the number of false positives. The recall is also measured to assess the frequency with which a PNG activates in response to *s*, given by:


$$\begin{array}{c} Recall=\frac{TP}{TP+FN}\;, \end{array}$$ (22)

where *FN* is the number of ‘false negative’ outcomes due to the PNG failing to activate in response to *s*. From Eqs (21) and (22), the F1 score is calculated as follows:


$$\begin{array}{c} F1\;Score=\frac{2\times Precision\times Recall}{Precision+Recall}\;. \end{array}$$ (23)

The above metrics are used to indicate the capability of PNGs in discriminating between visual stimuli with specific spatial features, and the reliability of their responses.

## Results

### Feature selectivity through network self-organisation

This section explores the emergence of feature selectivity in a hierarchical SNN model of the primate ventral visual pathway when presented with the collections of 2D object shapes shown in Fig 4 through a process of unsupervised competitive learning. Specifically, we investigate whether neurons in the higher layers develop selectivity to localised boundary contour elements and examine how these features are hierarchically represented across different spatial scales. This section culminates in assessing the resilience of the network’s feature selectivity when subjected to varying levels of input noise.

#### Formation of neurons that respond to boundary shape features.

**Fig 5:**
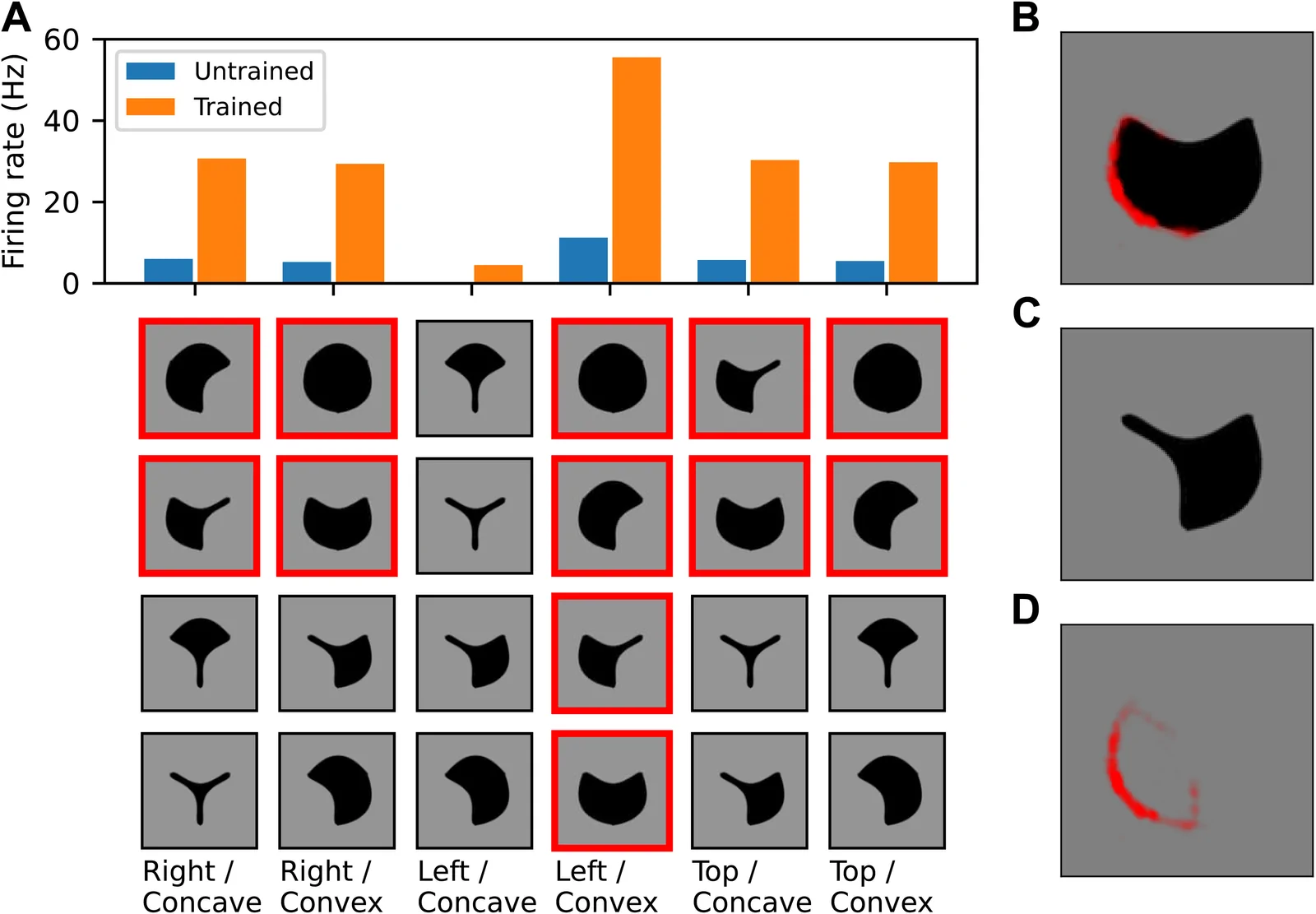
Development of boundary contour element selectivity in a final layer neuron trained on N3P2 shapes. (A) Firing rate responses, averaged across subsets of object shapes sharing a common feature, shown for the untrained network state (at its initialisation) and the trained network state (after STDP training). **(B)** Sensitivity of the neuron to input Gabor filters for a positive example (shape with a left-side convexity). **(C)** Sensitivity for a negative example. **(D)** Sensitivity averaged across all shapes. It can be seen that the neuron has learned to respond selectively to shapes with a left-side convexity.

Fig 5A depicts the average firing rate of an example neuron in the network’s final layer in response to subsets of object shapes within the N3P2 dataset (see Fig 4A), grouped according to the conformation of a boundary contour element. This neuron is shown because it exhibits strong selectivity for left-side convexity. Specifically, the neuron exhibits selective activation, demonstrating a more intense response to shapes possessing a left-side convexity compared to those with a left-side concavity. This distinct preference in the neuron’s response suggests specialised processing for this feature contour, indicating its discriminatory function.

Fig 5B–D presents a sensitivity analysis of this neuron with respect to the Gabor filters. By applying traceback analysis, which involves tracing the causal chain of feedforward connections weighted by intermediate neuronal activations and afferent synaptic strengths (see Methods section *Traceback analysis*), we identify the regions within the visual field to which the neuron is most sensitive. This analysis corroborates the firing rate observations, showing that the neuron has a marked preference for topological regions corresponding to the left-side convex boundary contour element.

In these experiments, shapes are presented at a fixed, centred retinal location during both training and inference. Consequently, selectivity to a particular object side and boundary conformation will appear retinotopically localised in the traceback maps (Fig 5B–D). The firing-rate comparisons in Fig 5A are therefore interpreted as boundary-conformation selectivity within this fixed retinal frame: for each side/conformation grouping shown, the plotted response is the neuron’s mean firing rate averaged over all shapes exhibiting that specified boundary element at that side, irrespective of the remaining sides.

**Fig 6:**
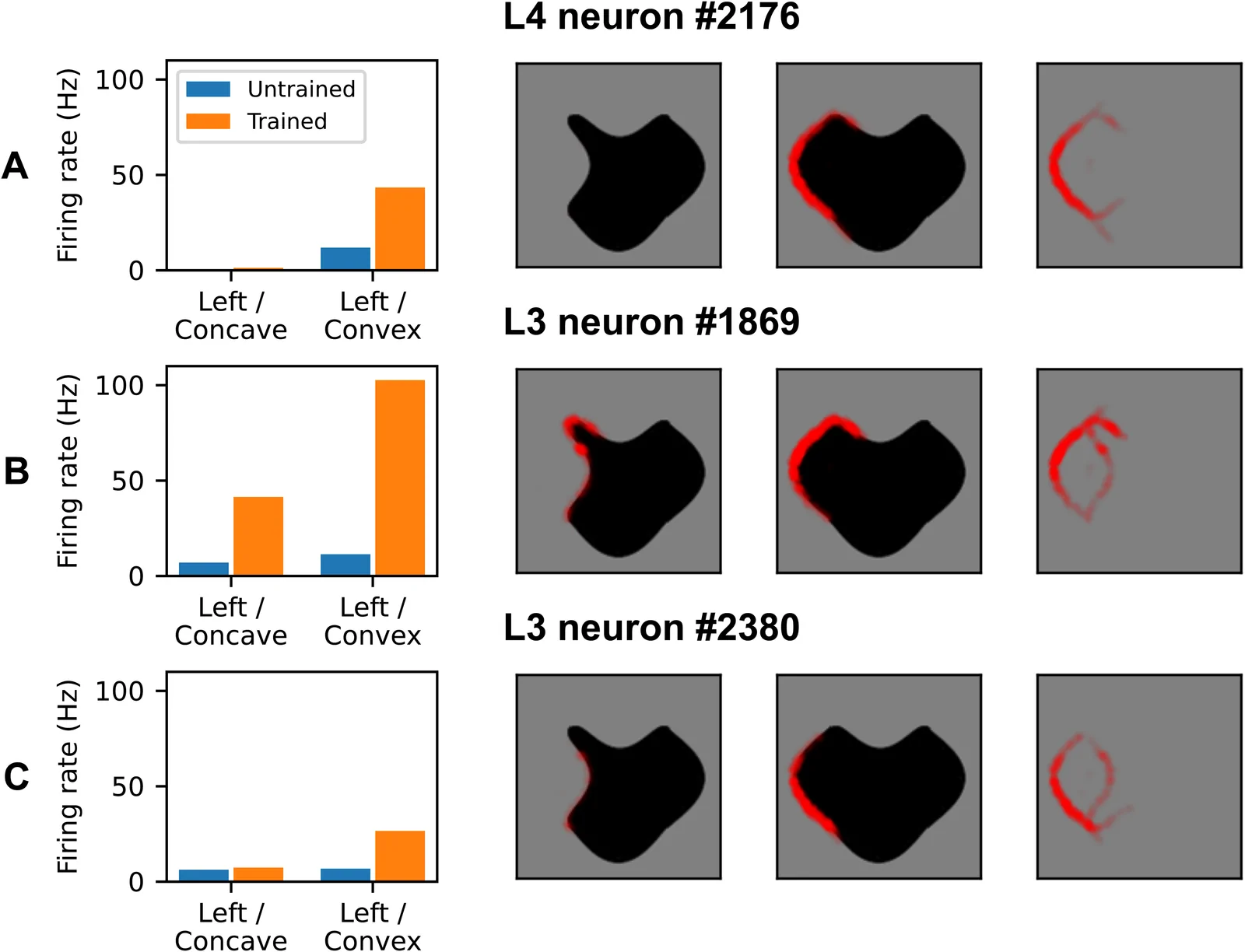
Hierarchical integration of neuronal responses for feature selective neurons in the last two layers, L3 and L4, trained on N4P2 shapes. Each row (A–C) shows the results for a different neuron. Row A shows results for a selected neuron in L4, while rows B and C show results for two selected neurons in L3. The left-most column depicts its averaged firing rate responses for shapes with / without a left-side convexity. The following two columns show its sensitivity to input Gabor filters when presented with example shapes. The last column shows its sensitivity averaged across all presented shapes. **(A)** The final layer neuron has afferent synapses from the other two neurons, rows **(B–C)**, which reside in the preceding layer. After training, it can be seen the L4 neuron appears to integrate/combine the boundary contour features represented by the two L3 neurons.

Fig 6 examines the hierarchical relationship between neurons in the third and fourth network layers in response to the more complex four-sided shapes from the N4P2 dataset (see Fig 4B). The left-most column of panels demonstrates the activity of neurons within layer L3 and their integration by a connected neuron in layer L4. The firing rate profiles across the N4P2 object subsets reveal that L3 neurons exhibit a marginal preference for left-side convexity. Correspondingly, the L4 neuron combines these responses, resulting in a more discriminatory activation profile with increased selectivity for this left-side convexity. These observations support the notion of hierarchical processing of spatial features in the network, indicating that the L4 neuron integrates the lower-level spatial features communicated by L3 neurons into a robust representation of the shape feature.

Also shown in Fig 6 is a traceback analysis for these neurons, effectively providing a visual representation of the neurons’ receptive fields with respect to the input layer. For the L3 neurons, their tracebacks reveal focused regions of the input space that elicit maximal responses, highlighting the narrower scope of their receptive fields. By contrast, the L4 neuron’s traceback presents a broad activation that encompasses the entire contour element that it represents, indicating its role as an integrator of the lower-level spatial features.

**Fig 7:**
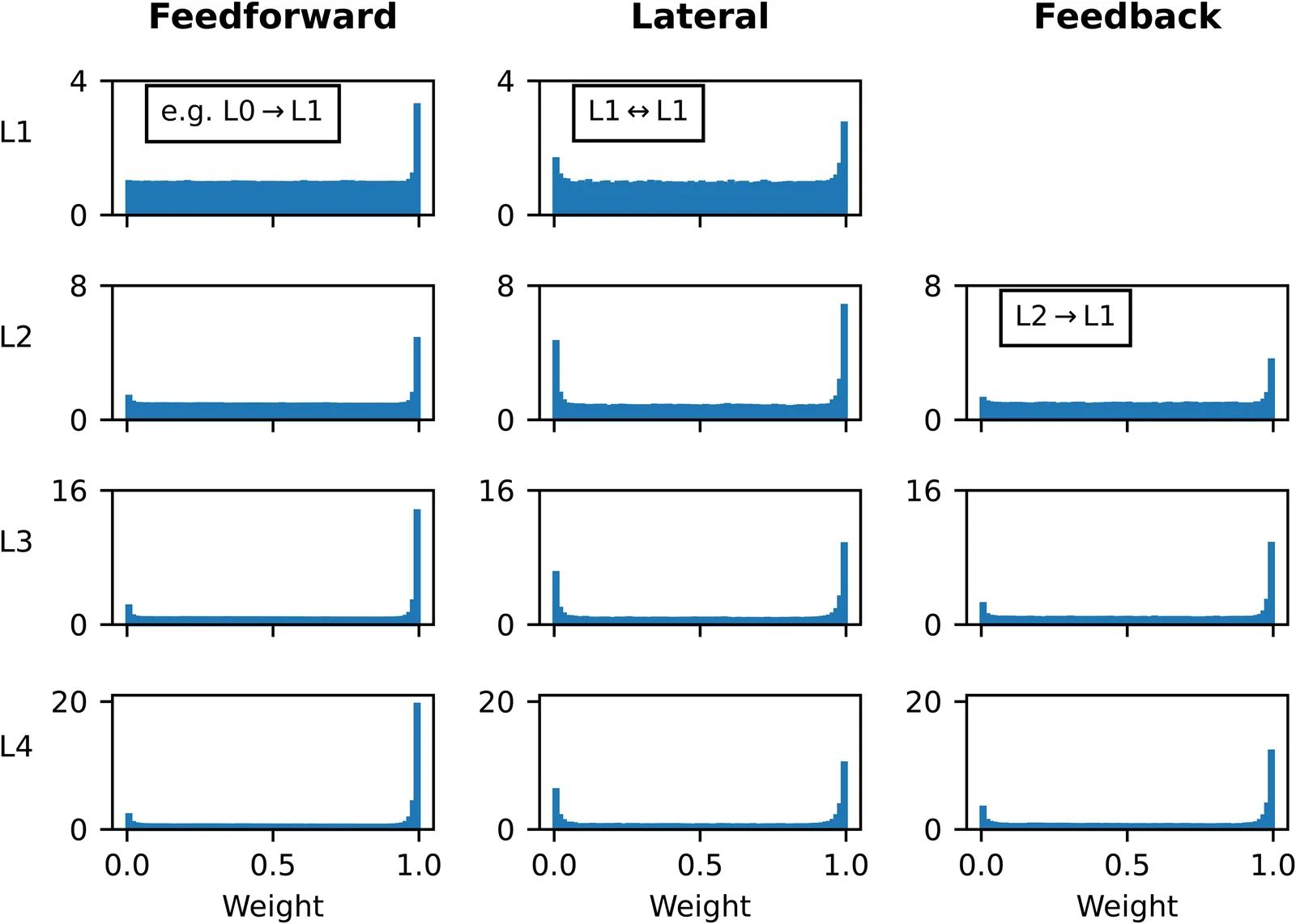
Bimodal distribution of synaptic weights in the network after training on N4P2 shapes, shown for modifiable connections between excitatory neurons. Each panel represents the relative frequency distribution of synaptic weights. The panels are organised by rows, corresponding to the network layer, where the type of synaptic projection is indicated by the column. These results were gathered from across three independent training runs. It can be seen that STDP training has resulted in bimodal distributions of synaptic weights, with many weights either fully potentiated or depressed. By comparison, the synaptic weights were initialised using a uniform distribution before training.

Fig 7 shows the synaptic weight distributions for the modifiable connections within the network following unsupervised competitive learning by STDP when exposed to the N4P2 dataset. As expected, STDP results in a bi-modal distribution of weights: synapses associated with regular pre- before post-synaptic firing events are potentiated to their maximum value, and vice-versa for post- before pre-synaptic firing events. Previous studies have shown that additive STDP results in such a bi-modal distribution, whereas multiplicative STDP leads to a unimodal distribution [44,45]. In the network, more neurons are activated in the higher layers, resulting in a larger proportion of potentiated synaptic weights, as reflected by the trend for feedforward synaptic projections. In contrast, a more balanced distribution of synaptic weights is apparent for the lateral connections, due to the competitive learning scheme which offsets potentiation in synaptic connections among neighbouring excitatory neurons against synaptic depression among more distantly connected ones. Feedback connections mirror the feedforward ones, forming an asymmetric distribution of synaptic weights skewed towards positive values. This supports recurrent activity in the network by introducing feedback loops, effectively providing a feedback mechanism from the final layer to the earlier layers.

**Fig 8:**
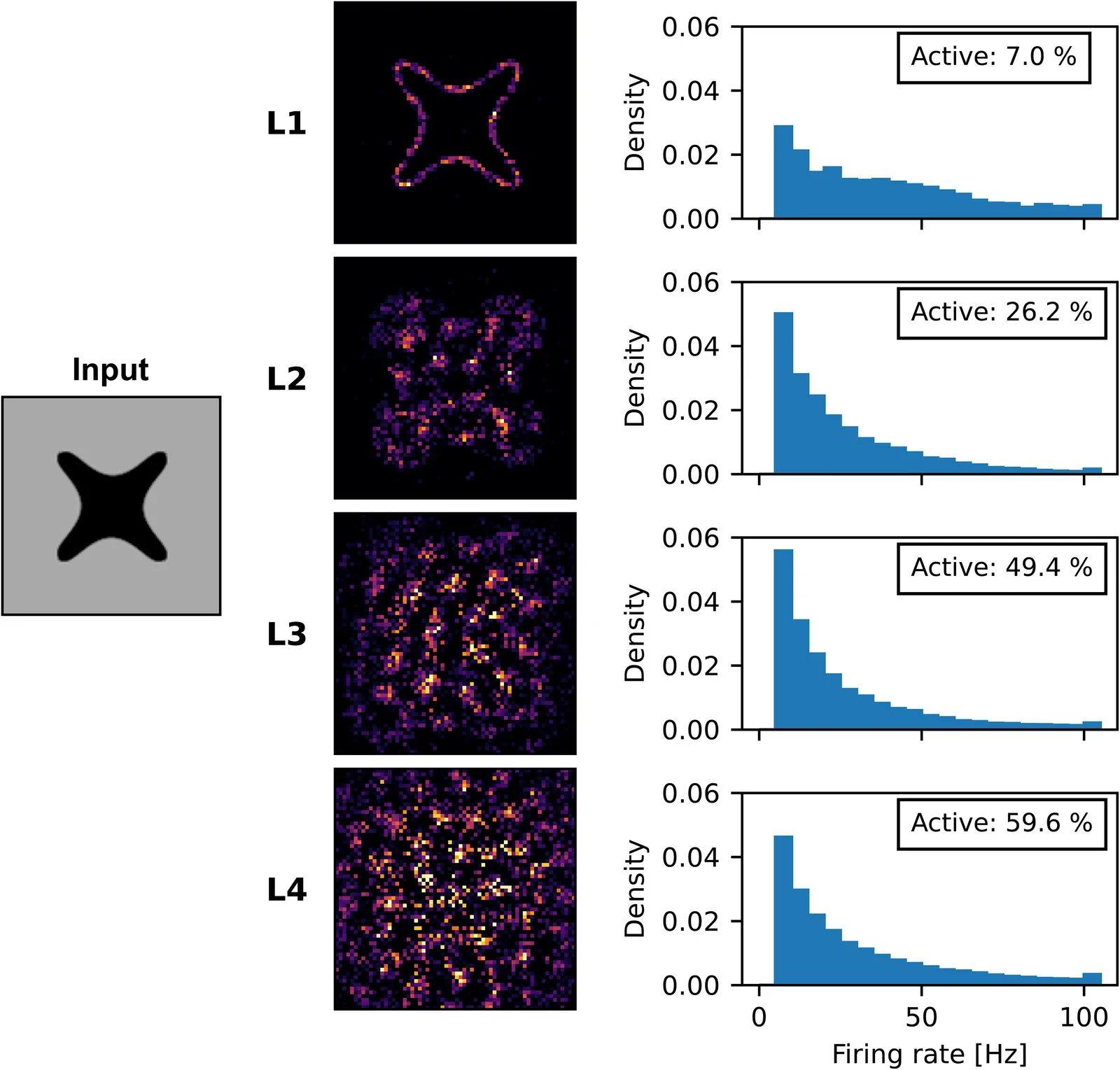
Progressive increase in the spatial extent and fraction of active excitatory neurons across successive layers after training on N4P2. This figure shows the neuronal responses to a presented four-sided object shape, depicted to the left. The following column displays the neuronal firing rates as spatial heatmaps, with each row representing a network layer. The final column shows the associated distribution of firing rates among active neurons, with inset text indicating the fraction of active neurons firing at least one spike.

Fig 8 demonstrates the topographic distribution of neuronal firing activity across the layers of the network, from the first layer to the last, along with the associated firing rates and fraction of active neurons. Clustered activity patterns are apparent, with localised groups of excitatory neurons co-activating while suppressing more distant ones due to lateral competition. The progressive spread of neuronal activity through the layers illustrates the hierarchical organisation of the network. Initially, a relatively small fraction of active neurons with localised feature maps is observed. As the input information propagates through the layers, the activity becomes increasingly diffuse. By the final layer, neurons integrate inputs over a much larger spatial scale, resulting in a higher fraction of active neurons capable of responding to the presented input shape.

#### Single neuron information analysis demonstrates increased feature selectivity.

**Fig 9:**
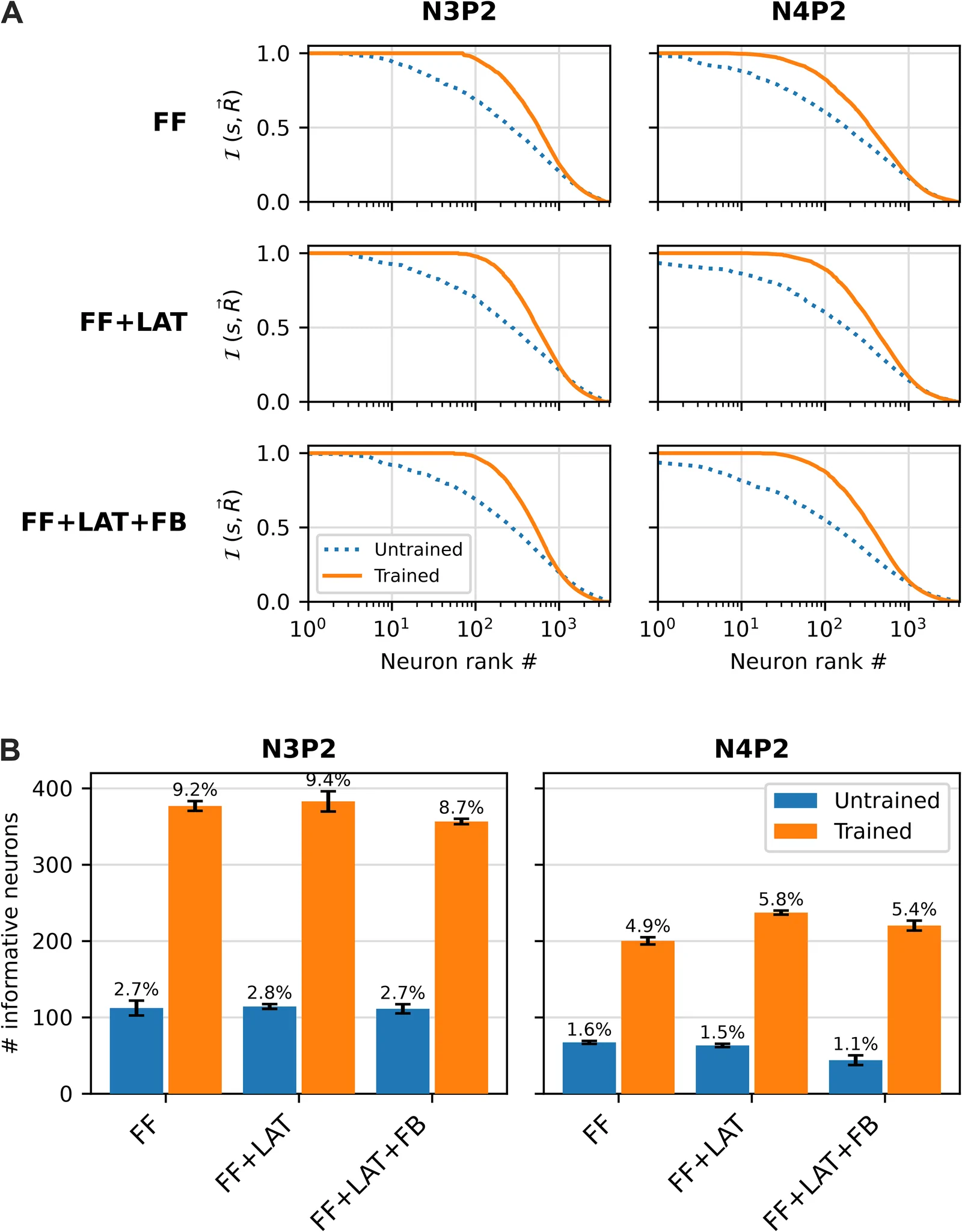
Single-neuron information analysis and informative-neuron counts for convex boundary contour elements. (A) Rank-ordered stimulus-specific information conveyed by output (L4) excitatory neurons regarding convexity (convex-vs-concave) at each neuron’s preferred object side. For each neuron, $ℐ(s,\vec{R})$ is computed separately for each object side and the maximum value across sides is retained, providing a location-independent summary; neurons are then rank-ordered by this maximum value. Curves show the mean ranked information profile averaged across three independent trials for networks before training (dotted line) and after training (solid line), for both datasets (N3P2 and N4P2) and for three network architectures (FF, FF + LAT, FF + LAT + FB). Here, ‘FF’, ‘LAT’ and ‘FB’ refer to the presence of feedforward, lateral and feedback synaptic connections between excitatory neurons, respectively. **(B)** Number of output neurons exceeding an information threshold of 2/3 bits based on the same per-neuron maximum across sides, shown as the mean ± s.e.m. across three trials. Percentages above bars indicate the proportion of the L4 excitatory population (out of *n* = 4096) exceeding the threshold. Overall, STDP increases both the information carried by output neurons and the number of informative neurons about convex boundary elements across datasets and architectures.

Fig 9A shows the amount of information conveyed by final layer excitatory neurons regarding convexity at their preferred object side. This is depicted as a rank-order plot, illustrating the effect of STDP in supporting the emergence of neurons that respond selectively to convex boundary elements (see Methods section *Single cell information analysis*). For a given training dataset, there is a significant increase in the informativeness of neurons after training, regardless of the network architecture. Throughout this section, we refer to the excitatory neurons in the final layer (L4) as the *output neurons*. A parallel analysis for concave selectivity, using the same rank-ordered stimulus-specific information measure is provided in S1A Fig.

Due to the inherent noise in our spike-based model that arises from input Poisson activity, we consider neurons to be sufficiently informative if they convey at least 2/3 bits regarding convexity at their preferred object side; because this analysis is binary (convex-vs-concave per side), the maximum stimulus-specific information is one bit. We therefore use 2/3 bits (i.e., ≥67% of the theoretical maximum) as a conservative cutoff for summary visualisation.

Based on this criterion, Fig 9B shows that a number of convex-informative neurons are already present in the untrained networks; the existence of informative neurons prior to training is expected, given the limited set of boundary contour elements from which the objects are constructed. However, after a period of network training, the number of informative neurons increases by several-fold. Neurons displaying an initial preference towards a certain boundary element are reinforced by STDP, forming more robust responses. Comparatively, networks trained on the N3P2 dataset show a higher number of neurons exceeding the 2/3 bits threshold than those trained on the N4P2 dataset. The corresponding concave-selective informative-neuron counts and proportions are shown in S1B Fig. Additionally, *side-resolved* single-neuron information analyses (reported separately for each object side, and for convex- and concave-selective neurons) are provided in S3 and S4 Figs. Comparing the convex and concave analyses (both the ranked profiles and thresholded counts) indicates a bias towards convex-selective responses in the final layer under the same criteria, the architectural origin of which we examine in the Discussion.

**Fig 10:**
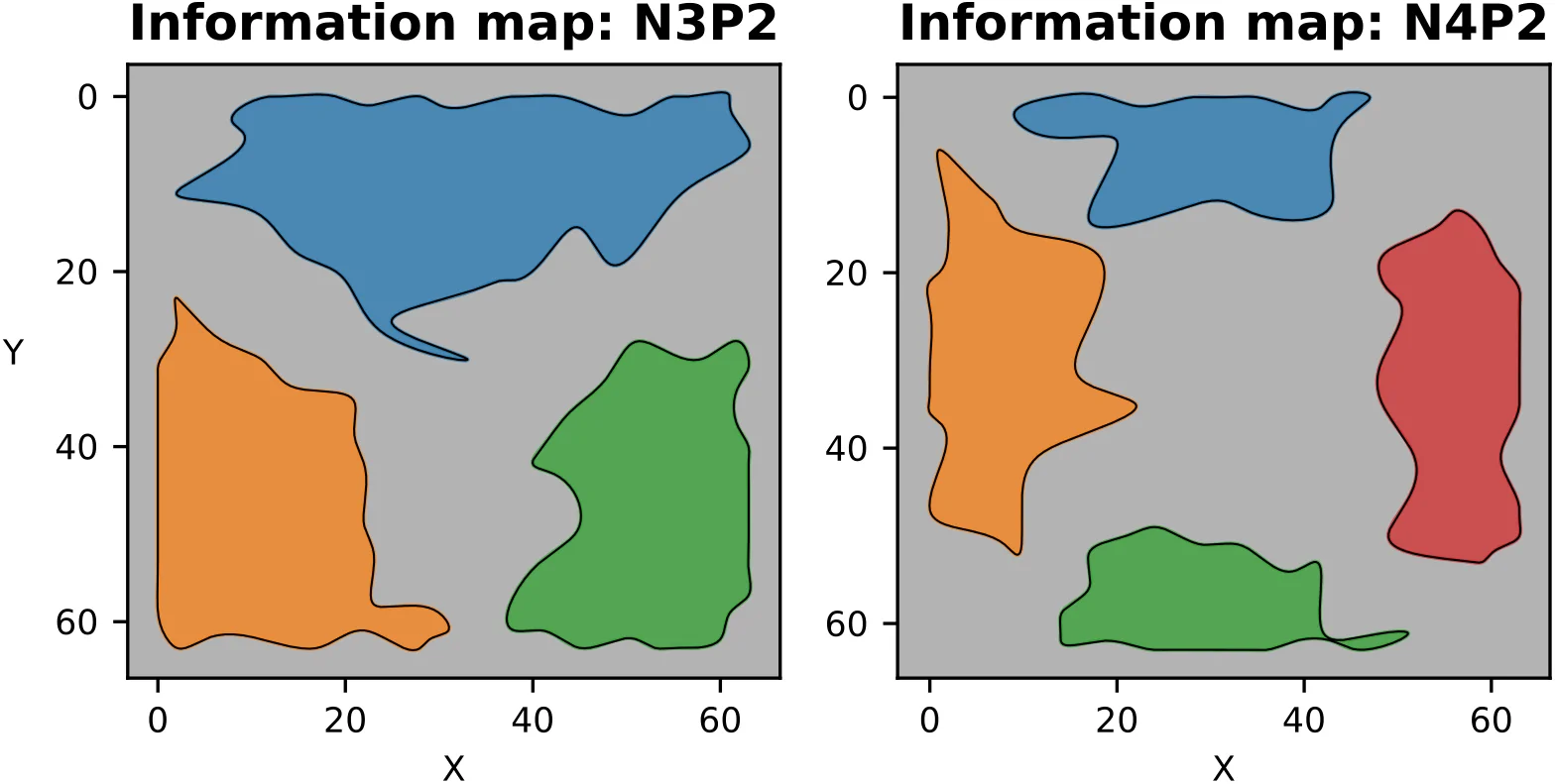
Self-organised feature maps in the final layer representing specific convex boundary elements after network training. These contour plots correspond to the distinct clusters of excitatory neurons in L4 which carry at least 2/3 bits of information regarding convexity at their preferred object side. This 64×64 final layer is topologically aligned with the 128×128 sized images, with wider spacing between output neurons to maintain alignment. Left: The blue, orange, and green regions correspond to neuron clusters selective to top, left, and right convex boundary elements, respectively. Right: The blue, orange, green, and red regions correspond to neuron clusters selective to top, left, bottom, and right convex boundary elements, respectively.

Shown in Fig 10 are information maps which depict the topological organisation of output neurons which were determined to carry at least 2/3 bits of information regarding convexity at their preferred object side (c.f. Fig 9B). Clusters of neurons emerge that are selectively responsive to specific shape features, with these clusters being topologically aligned with their preferred visual features. This alignment suggests that synaptic pathways are organised to project relevant visual information with minimal interference from other elements of the visual object. These findings are consistent with experimental studies that indicate a topographic arrangement of neurons within the ventral visual pathway, which are responsible for similar sensory functions [46–48]. It remains to be seen how these topological groupings might change when object shapes are translated across the retinal space during both training and testing. A corresponding information map for neurons selective to concave boundary elements is provided in S2 Fig.

#### Resilience of the network to input noise preserves feature selectivity.

To assess the resilience of the network to input noise, we investigate the performance of trained and untrained networks when varying levels of Gaussian noise are injected into the presented images. The objective is to establish the extent to which the neuronal response profiles developed through STDP confer robustness against increasingly variable incoming spikes, beyond that already present through variable Poisson input spikes, and if the network is still capable of demonstrating neuronal selectivity to specific boundary contour elements.

As previously, single neuron information analysis is used to measure the performance of a network containing all types of modifiable synapses, including feedforward, lateral, and feedback connections between excitatory neurons. Measurements are taken before and after network training based on inference spike recordings of final layer excitatory neurons. Gaussian noise is applied only during network inference, not during the training phase, and the images used in this case belong to the N4P2 dataset. Unlike Fig 9, here the stimulus-specific information is evaluated for a single fixed object side (the left-side boundary element) rather than taking the maximum across sides, in order to assess how resolving a localised feature degrades as input noise increases.

**Fig 11:**
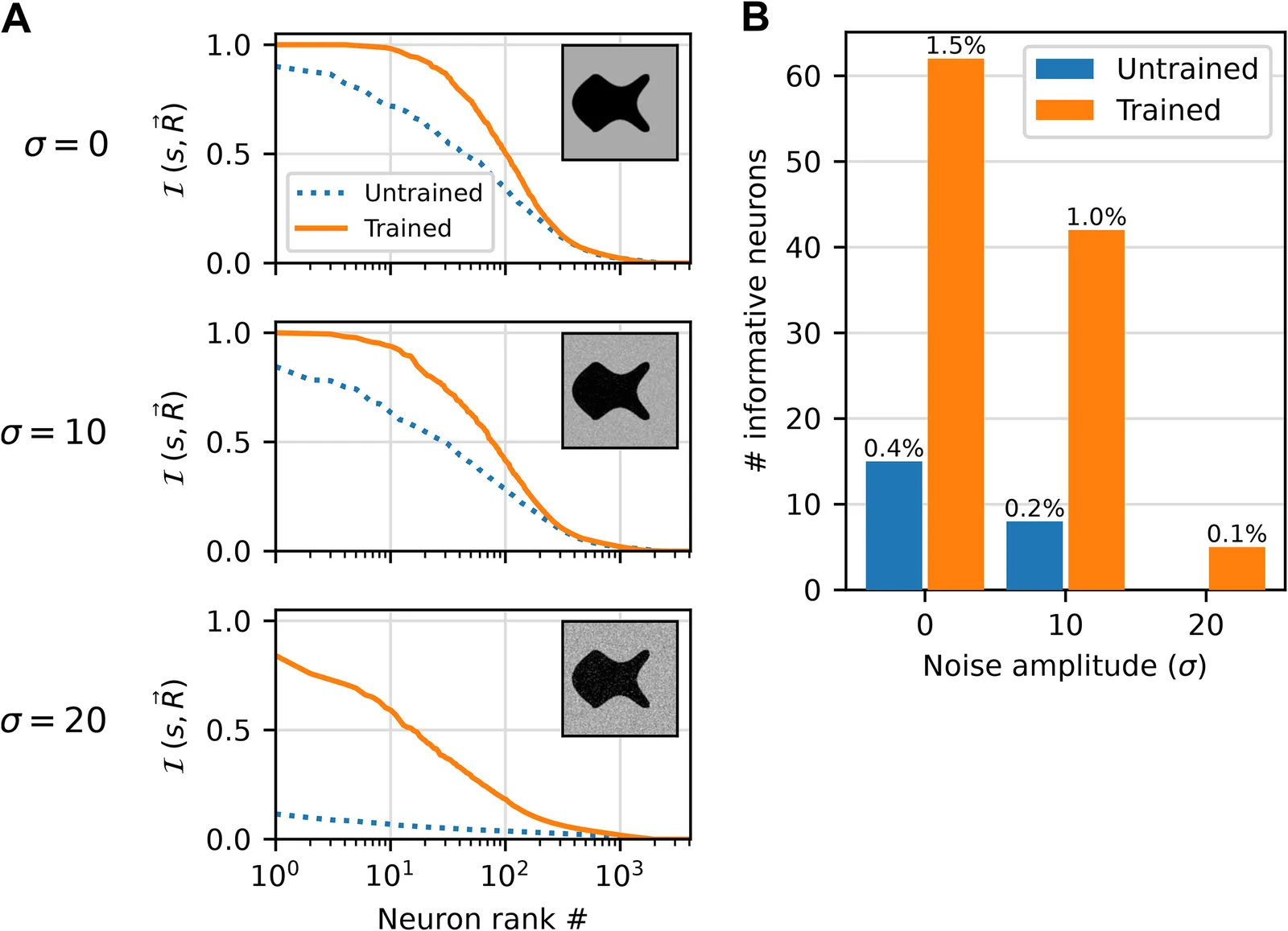
Network robustness to Gaussian input noise measured using single neuron information analysis. (A) Information conveyed by all final layer excitatory neurons regarding the *left-side* convex boundary element under different input noise amplitudes (σ), where the neurons are plotted in rank order; the inset panels show an example N4P2 test shape with the corresponding level of Gaussian noise applied for each σ condition. Unlike Fig 9, here the stimulus-specific information is calculated for this single fixed object side (left) rather than being evaluated across all sides and pooled. Measurements are shown for an untrained (dotted line) and trained (solid line) network state. **(B)** Number of output neurons conveying at least 2/3 bits of information about the left-side convex boundary element under different input noise amplitudes. The network architecture consisted of all types of modifiable synapses, including feedforward, lateral and feedback connections between excitatory neurons. The network was exposed to N4P2 object shapes subject to increasing noise levels. It can be seen that STDP training increases the amount of information carried by neurons about shapes containing this convex boundary element. Moreover, the recognition performance of the trained networks is more robust for increasing levels of image noise.

As expected, Fig 11A demonstrates an increase in the amount of information conveyed by output neurons regarding the left-side convex boundary element after training, which is evident for all levels of noise applied to the visual objects during testing. As the input noise increases, there is a slight decrease in the information conveyed by both trained and untrained neurons at an intermediate level of noise (σ=10), which becomes more substantial under the highest noise conditions (σ=20). Notably, the information content across final layer neurons in the untrained network approaches zero under high noise, effectively eliminating the chance selectivity of neurons that arises due to random network initialisation; to a large extent this effect is mitigated by network training.

Fig 11B quantifies the number of informative output neurons with respect to the left-side convex boundary element, based on a minimum threshold criterion for information conveyed (2/3 bits). This plot shows a substantial relative difference in the number of informative neurons before and after network training. This trend persists across increasing noise amplitudes, with only the trained network maintaining sufficiently informative neurons in the final layer under the highest noise condition.

For illustrative purposes, we compare the response properties of a select output neuron before and after network training with a high level of Gaussian noise (σ=20) applied to each visual object from the N4P2 dataset. The studied neuron had previously been identified as preferentially responding to shapes with a left-side convexity (L4 neuron #2176 depicted in Fig 6). As before, the neuron’s averaged firing rate responses are compared across two sets of shapes: those possessing a left-side convexity and those with a left-side concavity.

**Fig 12:**
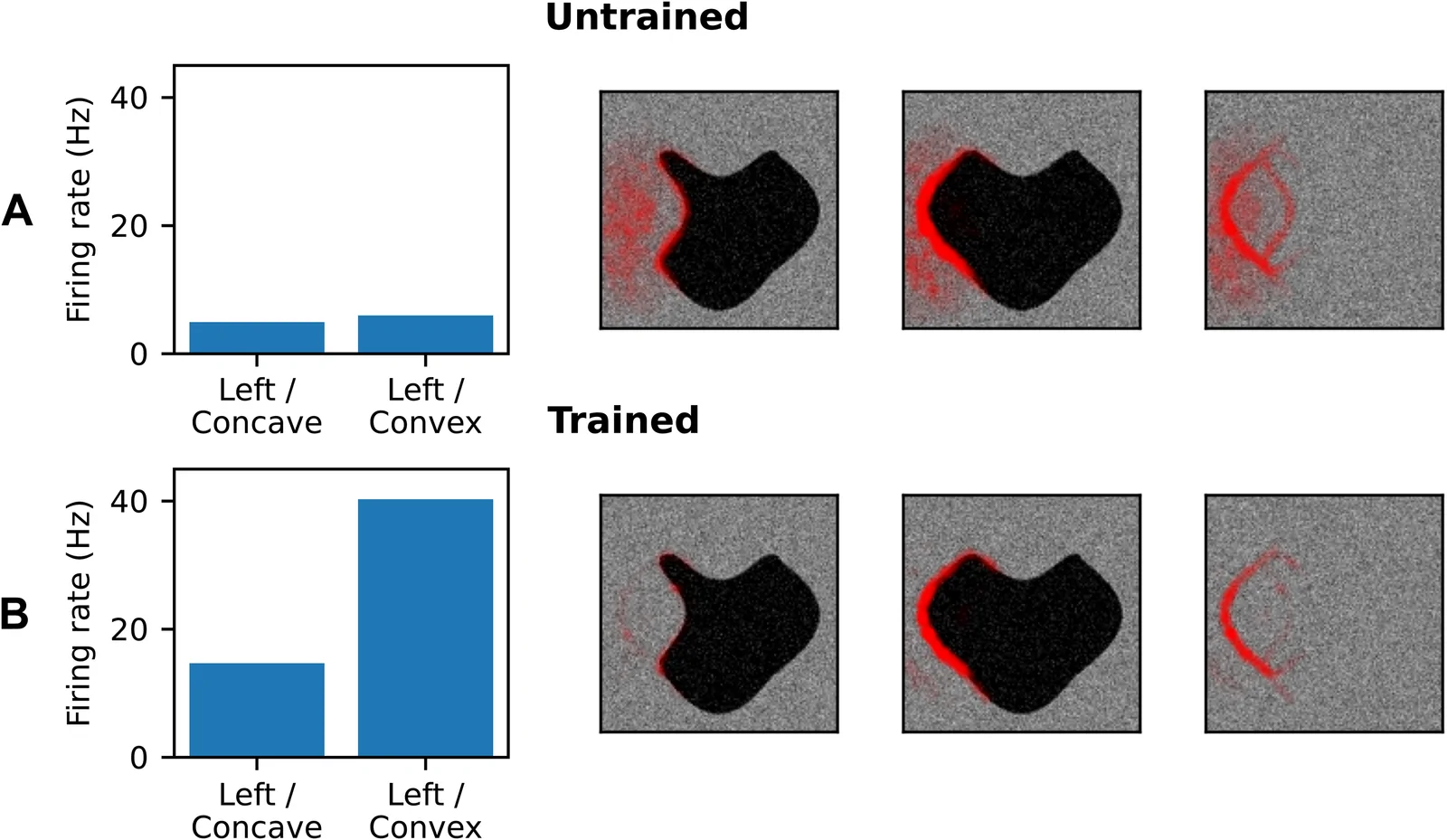
Neuronal response properties before and after training with Gaussian noise applied to shapes from N4P2. Measurements are taken from an output excitatory neuron (# 2176) in the final layer (L4), corresponding to the one depicted in Fig 6, but with input Gaussian noise (σ=20). **(A)** Neuronal firing responses and sensitivity maps before training. The left column shows the averaged firing rates in response to two sets of object shapes: those with a left-side concavity versus those with a left-side convexity. The next three columns illustrate the sensitivity maps of the neuron with respect to the input Gabor filters: (left) the neuron presented with a left-concave shape; (middle) presented with a left-convex shape; (right) the sensitivity averaged across all the presented shapes. **(B)** Similar to row (A) but determined after the neuron has been trained. It is evident that after training the neuron has learned to respond selectively to the left-side convexity.

The left-most column of Fig 12 shows a marked change in the neuron’s firing rate in response to left-sided concave and convex shapes after training. Prior to training, the neuron’s firing rate was approximately 5 Hz in response to both sets of shapes, indicating no significant selectivity. However, after training, the neuron’s firing rate increases to around 15 Hz and 40 Hz for concave and convex left-sided shapes, respectively, becoming tuned to this left-convex feature. This was the case despite Gaussian noise being applied to the visual objects during testing, further demonstrating that resilience is conferred to neurons through STDP.

The following columns of Fig 12 depict a traceback analysis for this neuron, showing the regions of the input layer, and by extension Gabor filters, that the neuron is sensitive towards. For the untrained neuron, diffuse regions of sensitivity are apparent, regardless of the example shapes presented to the network. By contrast, after training, the neuron forms a sharper sensitivity map that is more specific to a left-convex boundary element, demonstrating resilience against interference from the applied Gaussian noise.

#### Feature selectivity is resilient to partial visual occlusion.

To evaluate the robustness of learned feature representations against missing visual data, we subjected the trained network to an occlusion test where the left-convex boundary contour of the input images is progressively masked. This determines the extent to which learned feature selective responses degrade as parts of the stimulus are hidden, an important consideration for robust object recognition under partial occlusion.

**Fig 13:**
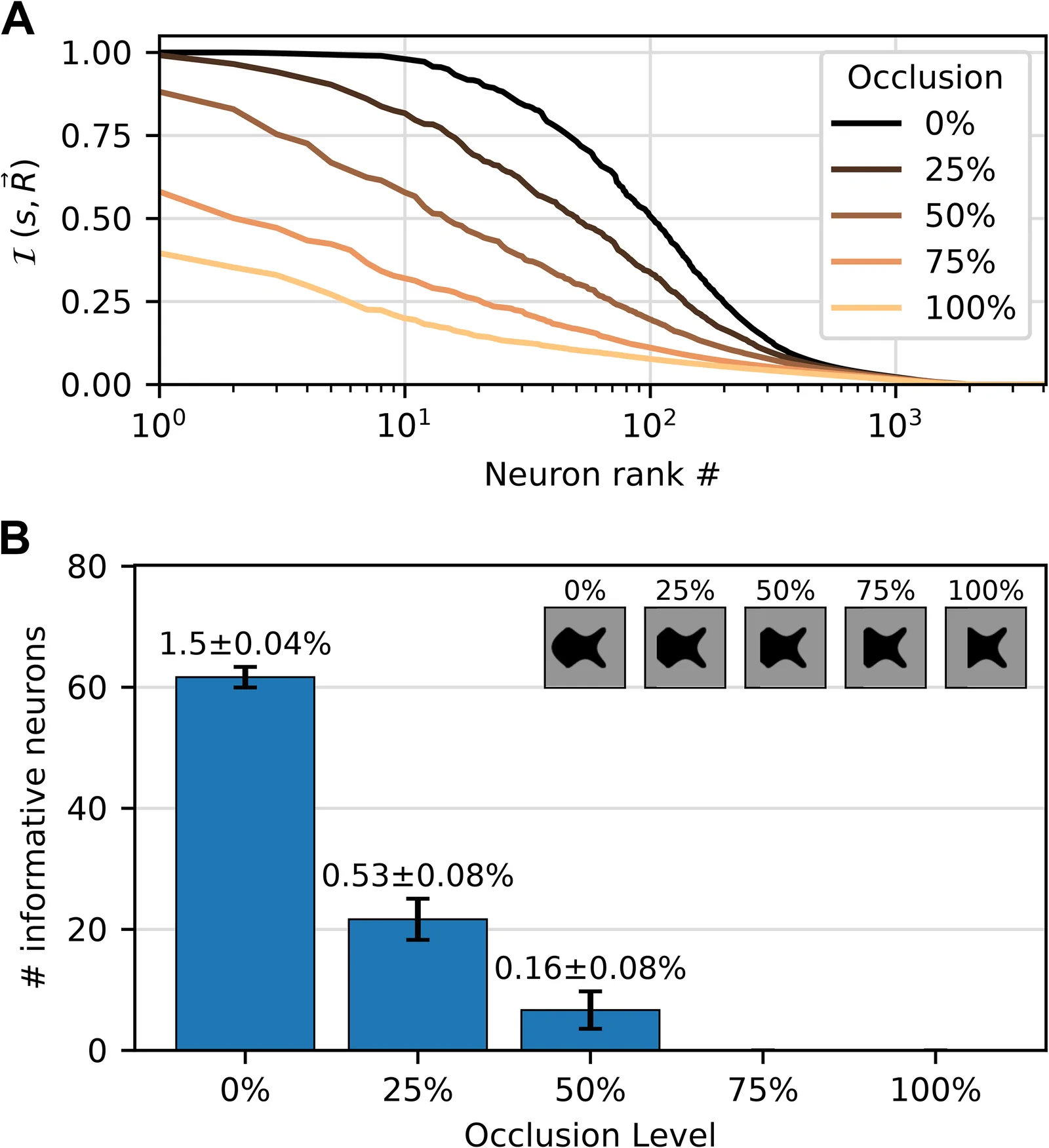
Robustness of learned feature selectivity to progressive visual occlusion. (**A)** Stimulus-specific information conveyed by final layer excitatory neurons regarding the *left-side* convex boundary element, plotted in rank order for increasing levels of feature occlusion (0% to 100%) and averaged across three independent trials. **(B)** Number of output neurons conveying at least 2/3 bits of information about the target feature as a function of occlusion level (mean ± s.e.m.). Insets illustrate the “sliding curtain” mask applied to the specific pixel region of the target feature on an example N4P2 shape. The trained network demonstrates graded degradation, maintaining a population of selective neurons even when the diagnostic feature is partially hidden (25–50%), before selectivity collapses as the feature is completely obscured (75–100%).

Final layer excitatory neuron spike recordings were gathered from three independently trained networks on the N4P2 dataset using a “sliding curtain” protocol that progressively masked the Gabor filter outputs (L0 Poisson input activations) within a vertical strip corresponding to the left-side convex boundary element, across all Gabor channels. The mask width was varied to occlude from 0% to 100% of the feature’s spatial extent (Fig 13B, inset), while keeping the rest of the image visible. The analysis focused on the stimulus-specific information conveyed by output neurons regarding the presence of this partially obscured feature.

Fig 13A shows the ranked stimulus-specific information for varying degrees of occlusion. At low occlusion levels (0–25%), the top-ranked neurons maintain high information values (approaching 1 bit), indicating that the network can reliably identify the feature even when it is partially hidden. As expected, this drops off rapidly as the feature becomes fully obscured. This resilience is further quantified in Fig 13B, which tracks the number of informative neurons (exceeding 2/3 bits). While the population of identifying neurons naturally decreases as the diagnostic information is removed, a functional subset of neurons retains selectivity up to 50% occlusion, demonstrating that the learned responses tolerate significant loss of afferent drive before recognition fails.

### Emergence of polychronization supports hierarchical feature binding

This section investigates the extent to which groups of neurons in the higher network layers exhibit the property of polychronization, characterised by the emission of spikes in regularly repeating spatiotemporal patterns (PNGs). Additionally, we explore whether these PNGs form three-neuron HFB circuits that encode the hierarchical binding relationships between boundary contour elements at different spatial scales (see Fig 2). For tractability, we restrict our analysis to a specific type of HFB circuit, where both the high-level and binding neurons reside in the layer that immediately follows the low-level feature neuron, although other types of binding circuits are possible.

#### Identifying PNGs across different network architectures.

To quantify the extent of polychronization in the network, we use the SPADE method to detect the number of unique PNGs that structurally correspond to a three-neuron HFB circuit (see Methods section *Polychronous neuronal group detection*). This detection is applied to various network architectures to establish their influence on PNG formation, considering different configurations of modifiable feedforward, lateral, and feedback connections between excitatory neurons. The number of detected PNGs is recorded for each network layer, with the layer assignment based on the second-firing neuron that constitutes a PNG.

**Fig 14:**
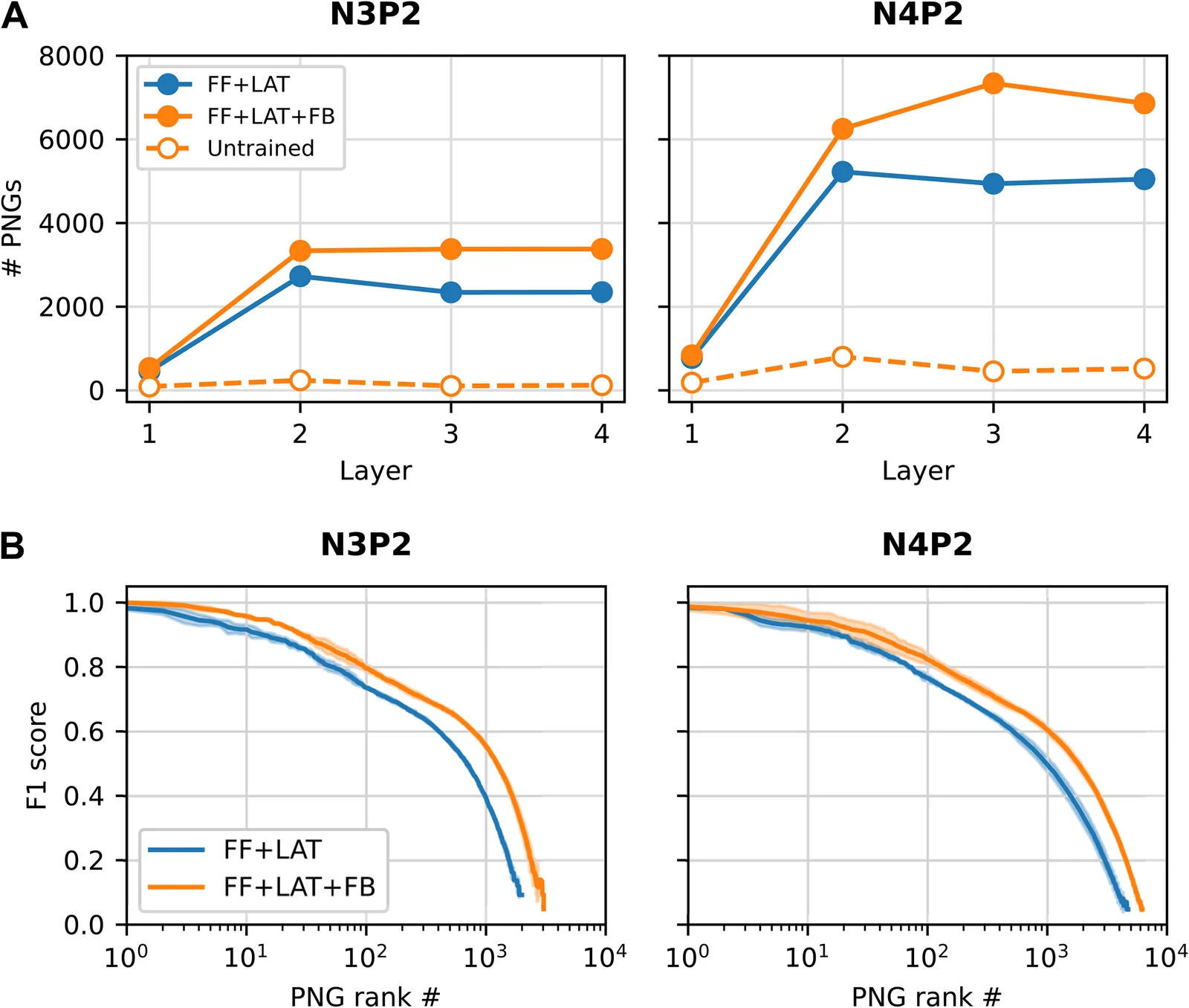
Emergence and representational performance of PNGs in hierarchical networks. (A) The count of distinct PNGs detected in each network layer for datasets N3P2 (left) and N4P2 (right). Results compare trained networks (solid lines) with untrained networks (dotted lines). These PNGs structurally correspond to three-neuron HFB circuits, where the layer of each PNG count is assigned based on that of the second-firing neuron. **(B)** The representational performance of PNGs detected in the final network layer regarding convex boundary elements, shown as a rank-order plot of F1 scores for N3P2 (left) and N4P2 (right). In both panels, networks act under two configurations: feedforward and lateral connections (FF + LAT) or with added feedback (FF + LAT + FB). Error bars/shading indicate standard deviation across three independent runs. In **(A)**, the mean relative error is 4.5%, rendering error bars smaller than the markers. As shown by the negligible counts in **(A)**, STDP training is necessary for PNG emergence; the inclusion of feedback connections increases both the quantity of PNGs formed (A) and their classification performance **(B)**.

Fig 14A presents the number of distinct PNGs detected across different layers of the network for both the N3P2 and N4P2 datasets. Following training, the number of PNGs increases significantly in layers 2, 3, and 4, suggesting that the network is more capable of developing HFB relationships in the higher layers. This trend is consistent across both network configurations (FF + LAT and FF + LAT + FB), indicating the effectiveness of unsupervised competitive learning in supporting precisely coordinated PNGs. The notable absence of PNGs in the first layer is expected, given that input spikes were randomly distributed according to a Poisson process, resulting in a lack of temporal and spatial correlation between pre- and postsynaptic spike trains. It is also apparent that STDP is necessary for the emergence of polychronization, based on the minimal number of PNGs in the untrained network across all layers.

There is a greater number of PNGs formed in response to the N4P2 dataset as compared to those formed in response to N3P2. We suggest two main factors that underlie this difference. Firstly, the twice as many shapes in N4P2 leads to twice the number of collected spike recordings. This inherently leads to more PNGs being detected. Secondly, we speculate that more PNGs are formed in response to the four-contour shapes due to the increased diversity of spatial features within the shapes available for the PNGs to relate.

Notably, Fig 14A demonstrates that the inclusion of feedback connections (FF + LAT + FB) results in a significantly higher number of PNGs compared to the network with only feedforward and lateral connections (FF + LAT). This improvement was consistent across three independent simulations per architecture, with a two-way ANOVA (analysed layers L2–L4) confirming a robust main effect of architecture for both the N3P2 (*F*_1,12_ = 242.2, *p* < 0.001) and N4P2 (*F*_1,12_ = 512.1, *p* < 0.001) datasets. Additionally, a significant interaction in both datasets (*p* < 0.05) revealed that this benefit was amplified in the deeper network layers (L3 and L4).

To quantify how detected patterns are filtered by our post-processing criteria and surrogate-based significance testing, we report the number of triplet patterns at each stage of the detection pipeline (detected, eligible, and significant), broken down by layer, in S5 Fig. This shows the attrition from the initial set of detected triplets after enforcing the HFB eligibility criteria and statistical significance testing.

#### Sensitivity of PNG detection to temporal span and timing tolerance.

We now determine the extent to which observed PNG counts depend on the SPADE temporal-span constraint and the imposed timing tolerance used for delay matching. Across detected patterns, PNG temporal spans were broadly distributed, and relaxing the maximum-span threshold did not reveal an abrupt increase that would indicate our default span (20 ms) was truncating the number of eligible patterns; as expected, increasing the timing tolerance monotonically increased retained PNG counts (see S7 Fig). Together, these analyses support our conclusions that PNG prevalence is not an artefact of a narrowly chosen detection window.

#### PNG selectivity for convex boundary elements.

The representational performance of PNGs is assessed using the F1 score when classifying the convex boundary elements of shapes from the N3P2 and N4P2 datasets. For this task, when a PNG is activated at least once during the presence of a particular convex element, it is considered as a positive identification of that element. A concave element in the same position is considered as the corresponding negative class. Using these as ground truth labels, the TP, FP, and FN rates are calculated for each PNG-element pairing, and the F1 score is calculated for PNGs in cases where the precision >0.5.

Each evaluated PNG is screened to consist of a low-level and a high-level neuron both projecting to a feature binding neuron. This guarantees that all evaluated PNGs have the potential to act as HFB circuits, binding low-level and high-level features when activated. In this context, convex elements are considered the bound features, but without assumptions about the specific low and high-level features beyond their hierarchical positions, connections and conduction delays.

Fig 14B shows the F1 scores for PNGs across both datasets and network architectures, ranked from highest to lowest. While no PNG perfectly represents a convex element, between 10 and 40 PNGs in each task achieved high F1-scores (>0.9). The network with feedback connections consistently outperformed the one without. The larger N4P2 dataset produced more PNGs with sufficient precision (>0.5) compared to N3P2. However, the top-ranked N4P2 PNGs had slightly lower F1 scores and higher variability between runs, especially in the network with feedback connections. This suggests that increased shape complexity leads to more PNGs being formed and strengthened under STDP, but also creates a trade-off between complexity and reliable activation for a wider range of convex elements. We also performed a parallel analysis on the representational performance of PNGs with respect to concave boundary elements (see S6 Fig), demonstrating a bias towards convex selectivity. Overall, these results show that a subset of activated PNGs achieves a good balance between precision and recall in representing specific convex elements. This indicates their potential to encode high-level feature binding information as functional three-neuron HFB circuits.

#### HFB activations are selective for boundary shape features.

**Fig 15:**
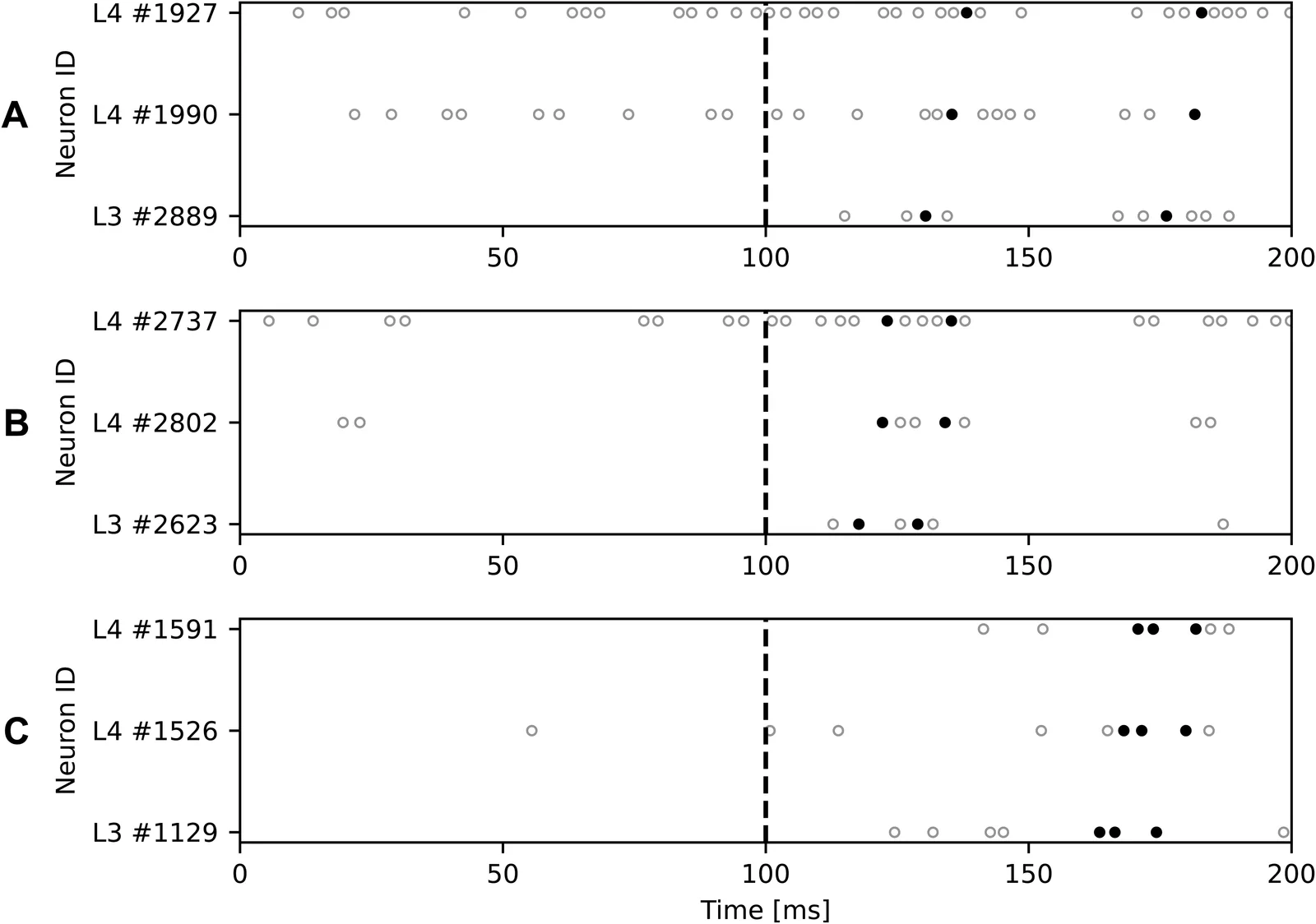
Spike rasters of neuronal activity involved in three PNGs that structurally conform to three-neuron HFB circuits. Each panel (A, B, C) corresponds to different shape configurations of objects from N3P2 with specific convex boundary elements: A) left-side convexity, B) right-side convexity, and C) top-side convexity. Each raster is divided by a vertical dashed line at 100 ms. The first segment (0-100 ms) shows responses to a shape with no convex boundary elements, while the second segment (100-200 ms) shows responses to a shape with a convex boundary element on a specific side. Neurons are ordered from top to bottom according to their role: binding, high-level feature, and low-level feature. The circular markers represent neuronal firing times, with solid circles indicating spikes participating in a PNG activation. Three-neuron PNGs can be seen in the last 100 ms of each of the subplots, A, B, and C.

**Fig 16:**
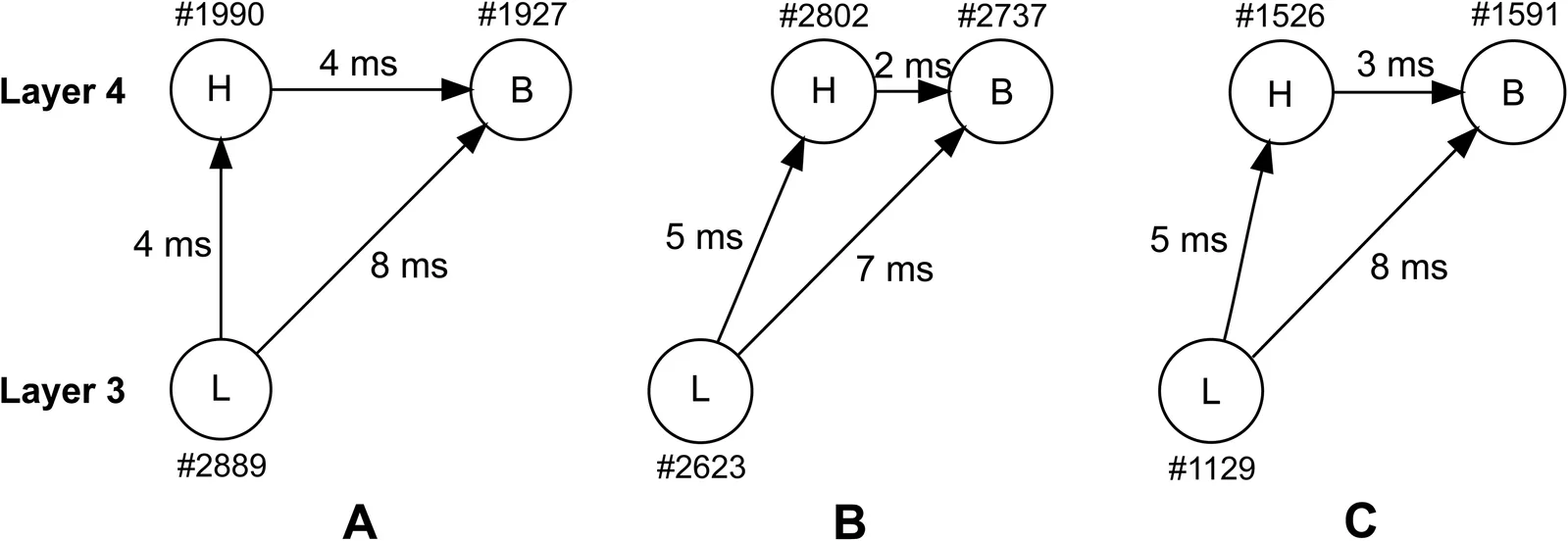
Three-neuron HFB circuits corresponding to the PNG activations in Fig 15. These circuits have a one-to-one label correspondence with the panels depicting the PNG activations in Fig 15. The node labels L and H represent low- and high-level feature neurons, respectively, while B denotes the binding neuron that encodes the hierarchical relationship between L and H. The label next to each node indicates the neuron ID, with neuron L residing in layer 3 (bottom node) and neurons H and B residing in layer 4 (top nodes). The measured spike-timing differences between neurons are annotated next to their respective connections. The circuits are of the form depicted in Fig 2, and have patterns of axonal conduction delays consistent with Eq (1).

To illustrate the activity and structure of representative HFBs, we examine three high-performing convex-boundary selective HFBs (F1-score > 0.9) specialised in detecting left, right, and top-side convexities (Fig 15). The initial 100 ms of the spike rasters depict neuronal responses to a shape lacking convex boundary elements, while the subsequent 100–200 ms period represents responses to shapes containing the preferred convex boundary. The corresponding circuit diagrams for these HFBs are presented in Fig 16.

In each instance, the HFB circuits activate two to three times in the presence of their preferred convexity and remain silent when the non-convex shape possessing no convex boundary elements is presented. This observation highlights distinct firing patterns among HFB neurons in response to shapes featuring their preferred convex boundary elements. Moreover, the second 100 ms segment exhibits greater regularity of activation compared to the initial 100 ms, suggesting some degree of firing rate selectivity among individual neurons. This underlying neuronal activity collectively supports the activation of the HFBs as a whole. Together, these findings demonstrate the network’s capacity for selective processing and differentiation of shape features.

A consistent temporal firing sequence emerges, characterised by the sequential activation of low-level feature neurons, high-level feature neurons, and binding neurons. This pattern, observed across multiple trials (as evidenced by two/three activations per panel in Fig 15), underscores the role of neuronal temporal coordination in HFB circuit formation. The binding neuron’s consistent firing following the activation of low- and high-level feature neurons suggests a causal relationship and implies its function in feature integration: the binding neuron fires whenever the lower-level feature neuron is participating in the firing of the higher-level feature neuron, and this carries information about the binding relationship between these two features. This supports the hypothesis that HFB circuits encode hierarchical binding relationships among visual features.

#### Reuse of feature and binding neurons preserves selective polychronous patterns.

The ability of neurons to participate in multiple pattern encodings simultaneously is a key aspect of polychronization theory. This property underlies the efficient information processing, high pattern encoding capacity, and spatiotemporal integration observed in spiking neural networks.

**Fig 17:**
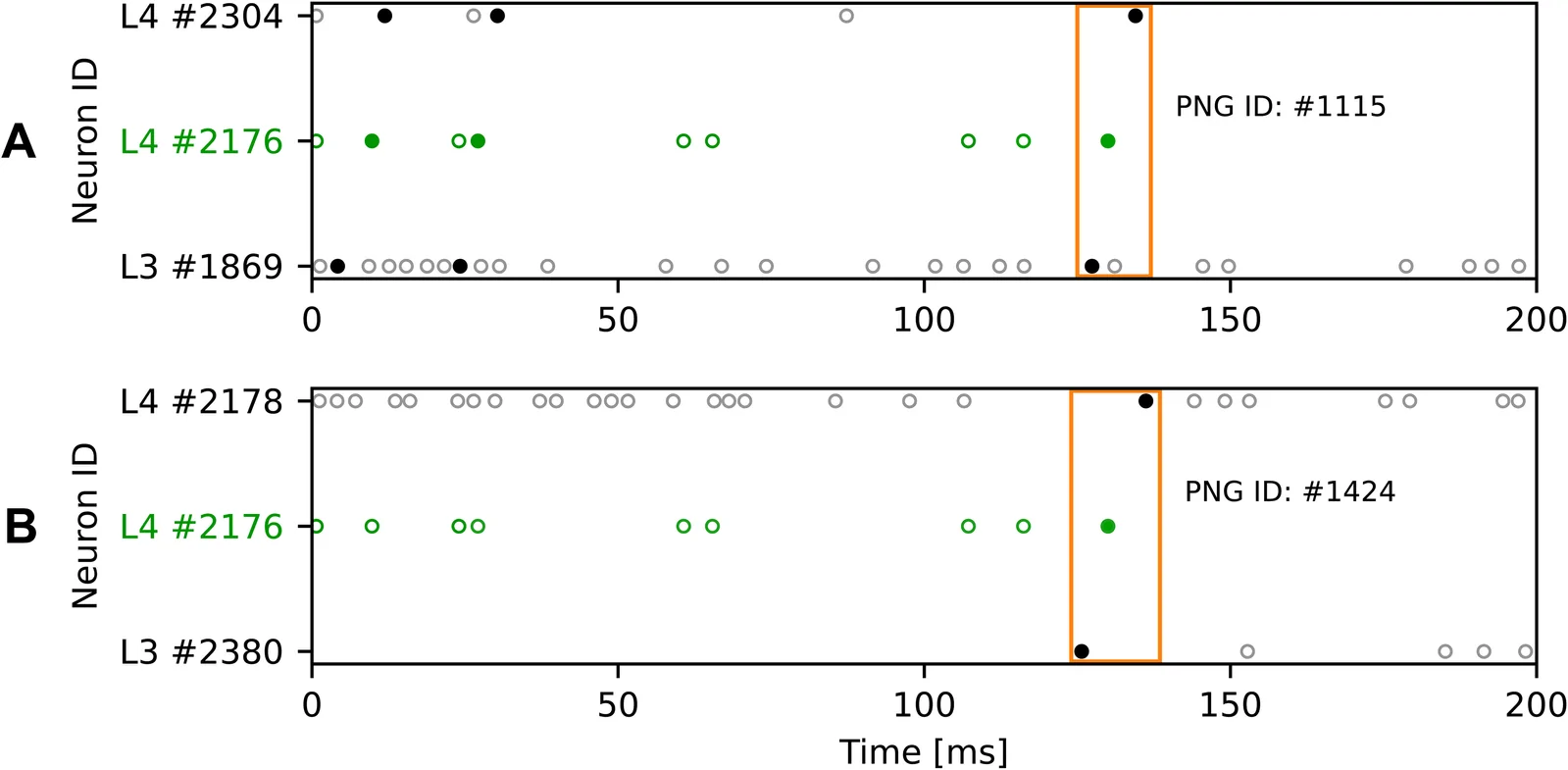
Spike rasters of neuronal activity involved in two PNGs: PNG #1115 (panel A) and PNG #1424 (panel B), in response to a left-side convex object shape from the N4P2 dataset. Each raster plots a three-neuron HFB circuit, with the high-level feature neuron (green) common to both PNGs. The top and bottom neurons are the binding and low-level feature neurons, respectively. Filled dots indicate spikes participating in PNG activations, and the orange box highlights the period of concurrent activation. Low- and high-level neurons are preferentially selective to left-side convex shapes (Fig 6).

We examined the responses of neurons in the last two network layers when presented with a shape from the N4P2 dataset exhibiting left-side convexity. Fig 17 displays the spike rasters of selected neurons in layers 3 and 4 involved in two distinct HFBs. These neurons preferentially respond to left-convex boundary elements of shapes from the N4P2 dataset. The high-level neuron in layer 4, shared by both HFBs, integrates information from lower-level neurons in layer 3 to represent the left-convex boundary element at a larger spatial scale (cf. Fig 6).

**Fig 18:**
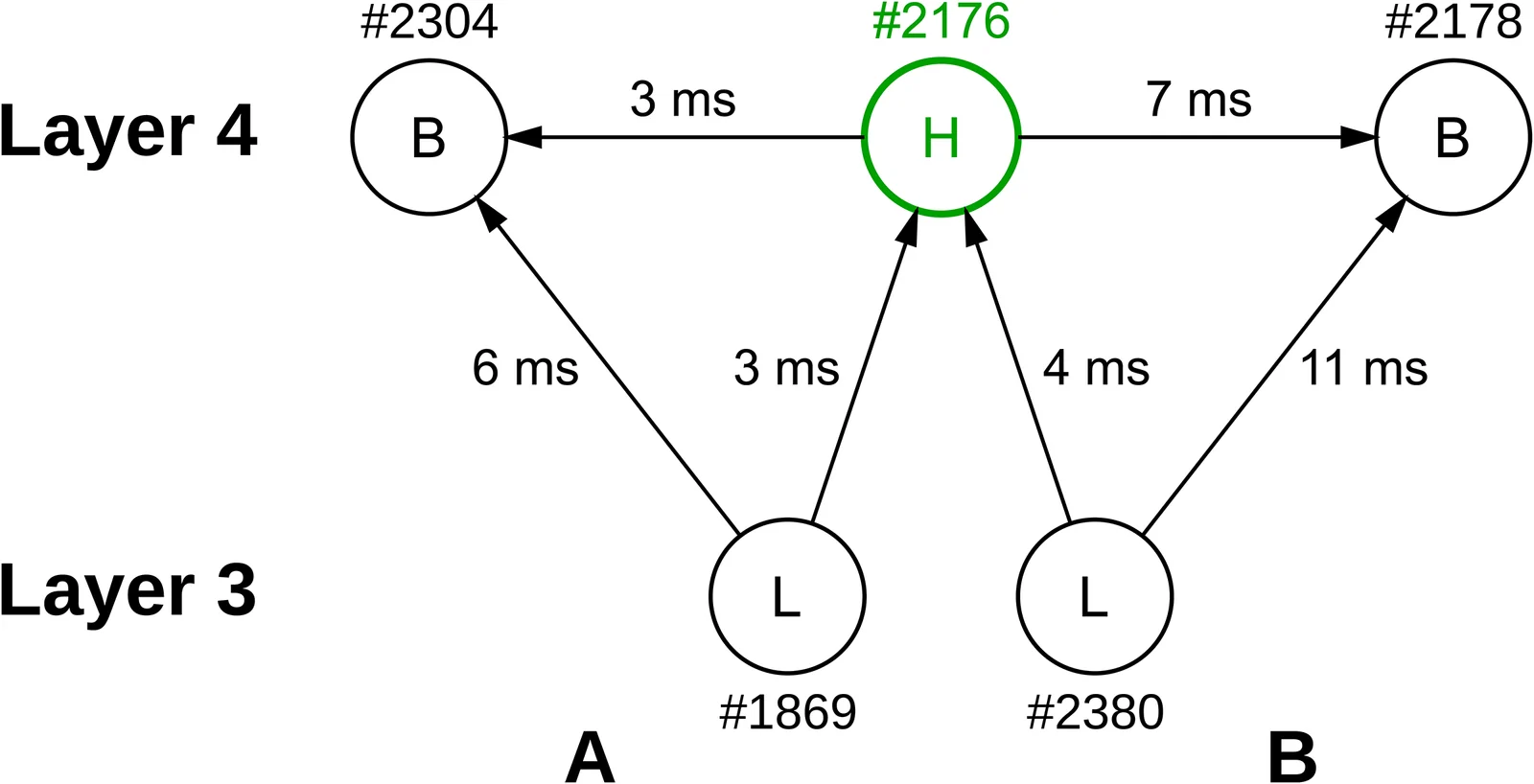
Three-neuron HFB circuits corresponding to the PNG activations in Fig 17. The green-edged H node corresponds to the shared high-level feature neuron that integrates information from multiple low-level feature neurons, denoted by **L.** In each triplet, a binding neuron, B, relates these representations that exist at different spatial scales. Labels and spike-timing differences follow the same conventions as described in Fig 16. Circuits A and B correspond to spike raster subplots A and B shown in Fig 17, respectively.

As shown in Fig 17, the high-level feature neuron (L4 #2176) is active in both depicted HFBs, as indicated by the orange bounding box. This region highlights the period during which the neuron participates in both pattern groups simultaneously. The structure of the two HFBs, with the shared high-level feature neuron L4 #2176 emphasised, is illustrated in Fig 18. The concurrent activation of this neuron suggests it integrates information from multiple lower-level neurons, supporting its role in hierarchical visual feature processing.

**Fig 19:**
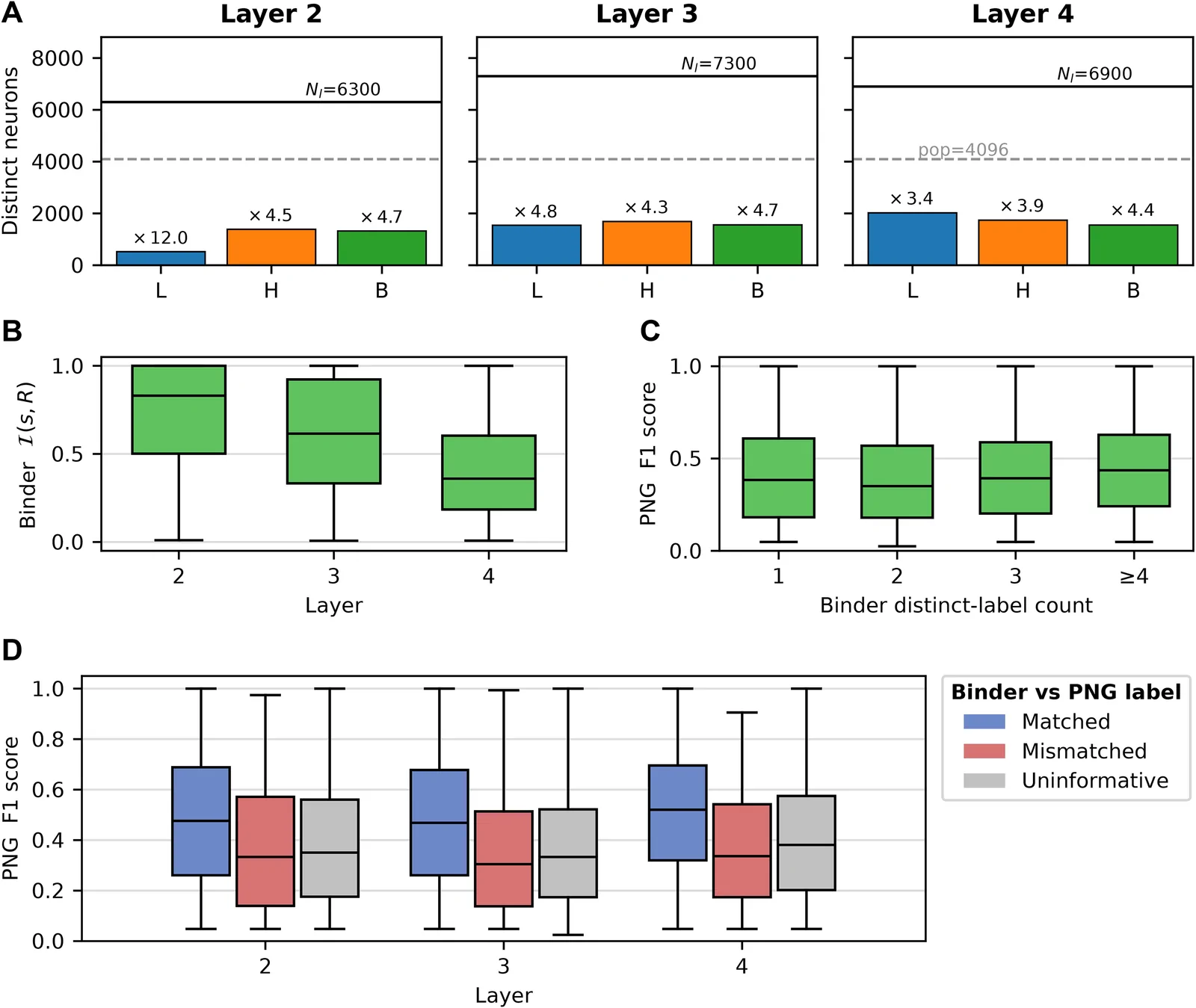
Reuse of low-level, high-level and binding neurons across PNGs conforming to HFB circuits, with the resulting binding-neuron ambiguity resolved at the level of the time-locked triplet. Results are shown for networks trained on N4P2 with all modifiable synapses (FF + LAT + FB). Each PNG is assigned to the anchor layer of its high-level (second-firing) neuron, so the low-level neuron of a layer-*l* circuit lies in layer l−1; PNGs anchored at layer 1 are omitted because their low-level neuron is a fixed Poisson input. **(A)** Number of distinct excitatory neurons occupying each circuit role, low-level (L, blue), high-level (H, orange) and binding (B, green), within the detected PNGs anchored at layers 2 to 4. The dashed line marks the per-layer excitatory population (4096 neurons) and the solid line the average number of PNGs anchored at that layer ($N_{l}$); the per-role reuse factor ($N_{l}$ divided by the distinct-neuron count) is annotated above each bar. **(B)** Distribution of the stimulus-specific information $ℐ(s,\vec{R})$ that each binding neuron conveys about its preferred boundary feature, grouped by anchor layer. **(C)** Distribution of PNG F1 score, for the PNG’s preferred boundary feature, against the number of distinct feature labels its binding neuron participates in across its circuits. **(D)** Distribution of PNG F1 score by anchor layer, with circuits split by whether the binding neuron’s own preferred label matches the PNG’s feature (matched, blue), differs from it (mismatched, red), or is undefined because the binding neuron is not individually informative and conveys less than 2/3 bits (uninformative, grey). Values reported in panel A are the mean, and panels B–D are pooled, across three independent detection trials. Overall, reuse of every circuit role, including the binding role, is expected when more binding relationships are represented than there are neurons, and the results are consistent with information about the bound feature being conveyed by the time-locked co-firing of the triplet rather than by the binding neuron alone.

The shared high-level neuron of Figs 17–18 is one example of a property that holds throughout the network. To quantify it, we counted the distinct excitatory neurons occupying each circuit role across the detected PNGs that structurally conform to HFB circuits, anchored at layers 2–4 (Fig 19A; the full per-role participation distributions are given in S8 Fig). At every layer the number of distinct neurons in each role lies well below both the excitatory population of 4096 and the number of PNGs anchored at that layer ($N_{l}$), so each role is reused across many circuits. For the binding neuron this reuse is unavoidable on the counts alone: there are at most 4096 binding neurons, yet they must account for the $N_{l}\approx 6300$ to 7300 PNGs anchored at each layer. The per-role reuse factor ($N_{l}$ divided by the distinct-neuron count) is between about 4 and 5 for the high-level and binding neurons and is broadly stable with depth, whereas the low-level pool feeding each layer grows as the layers are ascended.

These roles are shared rather than partitioned across distinct populations. Examining the full repertoire of each neuron (S8 Fig), within a given layer about two-thirds to three-quarters of the high-level neurons also act as binding neurons in other circuits, well above the roughly one-third expected were the roles assigned independently, since binding neurons make up about a third of the layer. Across layers, about 84% of the high-level and binding neurons in layers 2 and 3 additionally serve as the low-level neuron of a circuit one layer higher, reflecting the expected hierarchical composition. The binding role is therefore not an intrinsic property of particular neurons but a contextual one, set by which partners a neuron co-fires with in a given circuit, so a single neuron may act as a high-level feature neuron in one PNG and as a binding neuron in another.

A consequence of this reuse is that the binding neuron is individually ambiguous. The stimulus-specific information that each binding neuron conveys about its preferred boundary feature decreases with depth, with the median falling from about 0.83 at layer 2 to about 0.36 at layer 4 (Fig 19B), so the deeper binding neurons are individually uninformative. Correspondingly, the proportion of a binding neuron’s PNGs whose feature matches the binding neuron’s own preferred label falls from about 72% at layer 2 to about 41% at layer 4. A reused binding neuron thus fires across PNGs that encode different feature combinations, so its identity alone does not specify the bound feature, and this ambiguity grows with depth as binding becomes more combinatorial.

The results support the interpretation that information about the bound feature is represented at the level of the circuit rather than by the individual neuron. The F1 score of a PNG for its preferred feature is essentially invariant to the number of distinct labels its binding neuron participates in, including the high-F1 upper tail and even for binding neurons spanning four or more labels (Fig 19C), so reusing a binding neuron across feature combinations does not reduce the selectivity of the circuits it binds. Partitioning circuits by whether the binding neuron’s own preferred label matches the PNG’s feature (Fig 19D), the matched circuits have a higher median F1, but a substantial high-F1 tail persists in the mismatched and uninformative groups at every layer, including the deepest, where the binding neurons are most ambiguous; about 40% of strongly selective PNGs in layer 4 (F1 > 0.9) have a binding neuron that does not itself specify the bound feature, comprising about 38% in which the binding neuron is individually uninformative and about 2% in which it prefers a different feature. This pattern is consistent with the proposed role of the binding neuron as a coincidence detector: it fires when its low- and high-level partners arrive together at matched conduction delays, so an individually uninformative binding neuron is expected, and the persistence of high circuit selectivity in these cases supports the interpretation that information about the bound feature is conveyed by the time-locked co-firing of the triplet rather than by the binding neuron alone. Having established that reuse does not cost a circuit its selectivity, we next quantify how completely these circuits recruit the informative low-level features available to them.

#### Binding circuits cover the feature repertoire at moderate selectivity.

To assess whether the binding circuits represent the feature repertoire completely rather than sparsely, we compared the neurons recruited as the low-level member of at least one binding circuit selective for their own preferred feature, against the informative low-level population. Reuse was collapsed by counting distinct neurons, and recruitment was graded by circuit F1 score (S9 Fig). Coverage is complete at moderate selectivity: every feature is represented by at least one circuit exceeding an F1 score of 0.7 in both datasets (six of six for N3P2, eight of eight for N4P2), thinning to as few as four of six and six of eight at the strict F1 > 0.9 criterion, where the omitted features are always concave. A broad fraction of informative low-level neurons enter a labelled circuit, about 60% for N3P2 and 83% for N4P2.

Only a small fraction of informative neurons are recruited into a strongly selective circuit (about 4.2% for N3P2 and 3.8% for N4P2 at F1 > 0.9), and both this subset and the informative pool itself are convex-biased, with a convex-to-concave ratio of about 4.0 for N3P2 and 2.2 for N4P2 between the pools. The strongly selective representation is therefore biased toward particular features rather than uniformly sparse, and its presence in both datasets points to an architectural origin (addressed in the Discussion).

To test whether a recruited low-level neuron places the label of the circuit it joins, we measured, across all of its moderately selective binding circuits (F1 > 0.7), the fraction whose preferred feature matches the neuron’s own. This fraction is high, about 96% for N3P2 and 98% for N4P2, so a low-level neuron almost always sets the feature of the circuits it enters, and does so exclusively among the most selective circuits (F1 > 0.9). Within these same circuits, the high-level and binding neurons match the circuit’s label far less often (about 61% to 67%), which identifies the low-level neuron as the principal determinant of what a binding circuit encodes.

#### PNG onset timing and precision correlate with feature selectivity.

This subsection further investigates the temporal dynamics of PNGs activated in response to three-sided shapes with varying boundary configurations. Specifically, we aim to establish the distribution of their initial onset times in response to these shapes, and whether this correlates with their selectivity towards a specific convex boundary element.

For these simulations, the network included all modifiable synapses (feedforward, lateral, feedback), and inference was conducted after network training on the N3P2 dataset. To isolate the network responses to shapes with a specific convex boundary element and to account for residual activity between successive image presentations, the network was initially exposed to a ‘null’ shape consisting of only concave boundary elements before running inference on each of the test shapes. Accordingly, inference spike recordings were gathered for three distinct shapes, each featuring a single convex boundary element at one side (i.e., top, left, or right), for a duration of 200 ms per shape with ten independent repetitions and different random seeds (refer to Methods section *Training on visual stimuli and inference*). In this analysis, the first 50 ms of each recording was included to analyse the network’s early responses. Following the procedure described in Methods section *Polychronous neuronal group detection*, PNGs structurally conforming to three-neuron HFB circuits were identified from these spike recordings, restricted to those containing neurons spanning the final two network layers. To obtain reliable estimates of early onset times, identified PNGs associated with the null shape were excluded from this analysis.

**Fig 20:**
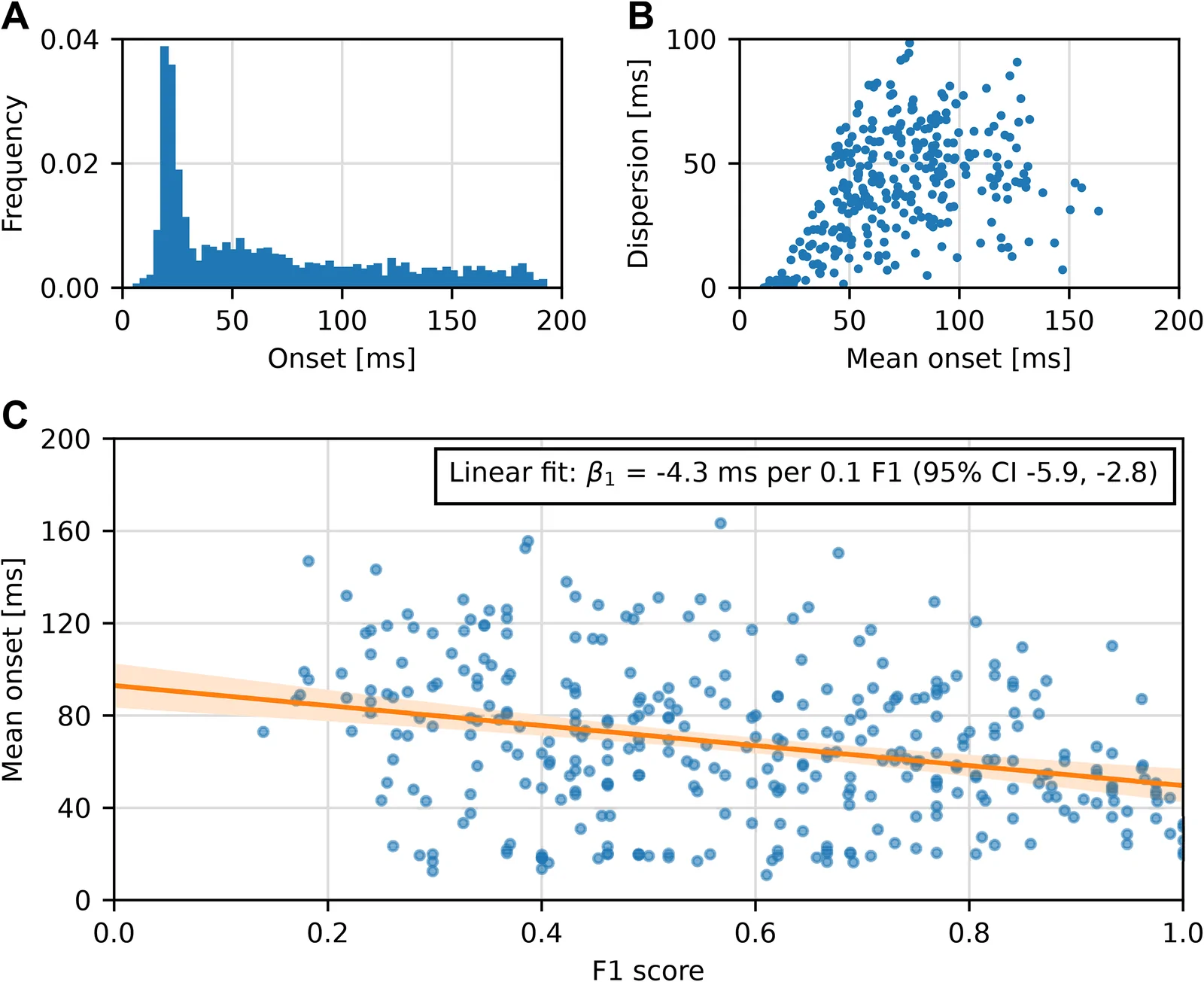
PNG onset timing, timing precision, and feature selectivity for three-sided shapes (N3P2). After training on N3P2 with all modifiable synapses (FF + LAT + FB), inference responses were recorded for three test shapes, each containing a single convex boundary element (top, left, or right), preceded by a concave-only *null* shape to reduce carry-over activity. Detected PNGs were restricted to those structurally conforming to three-neuron HFB circuits spanning the final two network layers (L3–L4), and PNGs associated with the *null* shape were excluded. **(A)** Empirical distribution of PNG onset times pooled across all PNG–side pairings and repetitions. **(B)** Timing dispersion (standard deviation) versus mean onset time computed across repetitions for each PNG–side pairing; only pairings with at least three recorded onset times are shown. **(C)** Mean onset time versus convex-boundary selectivity (F1 score) for each PNG–side pairing (*n* = 340). The line shows a least-squares fit and the shaded band indicates the 95% confidence interval for the fitted mean; a weak but reliable negative association is observed (*R*^2^ = 0.083, $p=6\times 10^{-8}$).

Fig 20 A–B shows the overall distribution of PNG onset times across all presented shapes, as well as the relationship between the mean and variability of onset times for individual PNGs responding to specific shapes. The histogram demonstrates a pronounced peak in PNG onset times around 20 ms, followed by a median value of 50 ms. This suggests that while most PNGs respond rapidly to the stimuli, a subset exhibits delayed activation, in some cases occurring as late as 200 ms. Regarding individual PNG responses, the scatterplot shows that PNGs with later mean onset times tend to exhibit greater variability in their activation times. This trend suggests that the precision of PNG activation timing decreases for those with later mean onset times, likely due to the accumulating effects of network dynamics and noise.

We next explore whether PNG onset timing is related to selectivity for a specific convex boundary element, quantified by the F1 score, treating this relationship as purely correlational without assuming a direction of influence. As shown in Fig 20C, across PNG–side observations (*n* = 340), onset time was weakly negatively associated with F1-scored selectivity (least-squares slope =−4.3 ms per 0.1 F1, 95% CI [−5.9,−2.8]; *R*^2^ = 0.083; $p=6\times 10^{-8}$). Thus, higher selectivity tended to coincide with slightly earlier onsets, although F1 explained only a small fraction of the variance in onset time.

We now examine the localised firing activity of an excitatory neuronal population within layer L3 of the network in response to shapes with or without a particular convex boundary contour element. This analysis focuses on how the early activation times of a PNG, which is selective for this boundary element, relate to the activity of nearby neurons.

**Fig 21:**
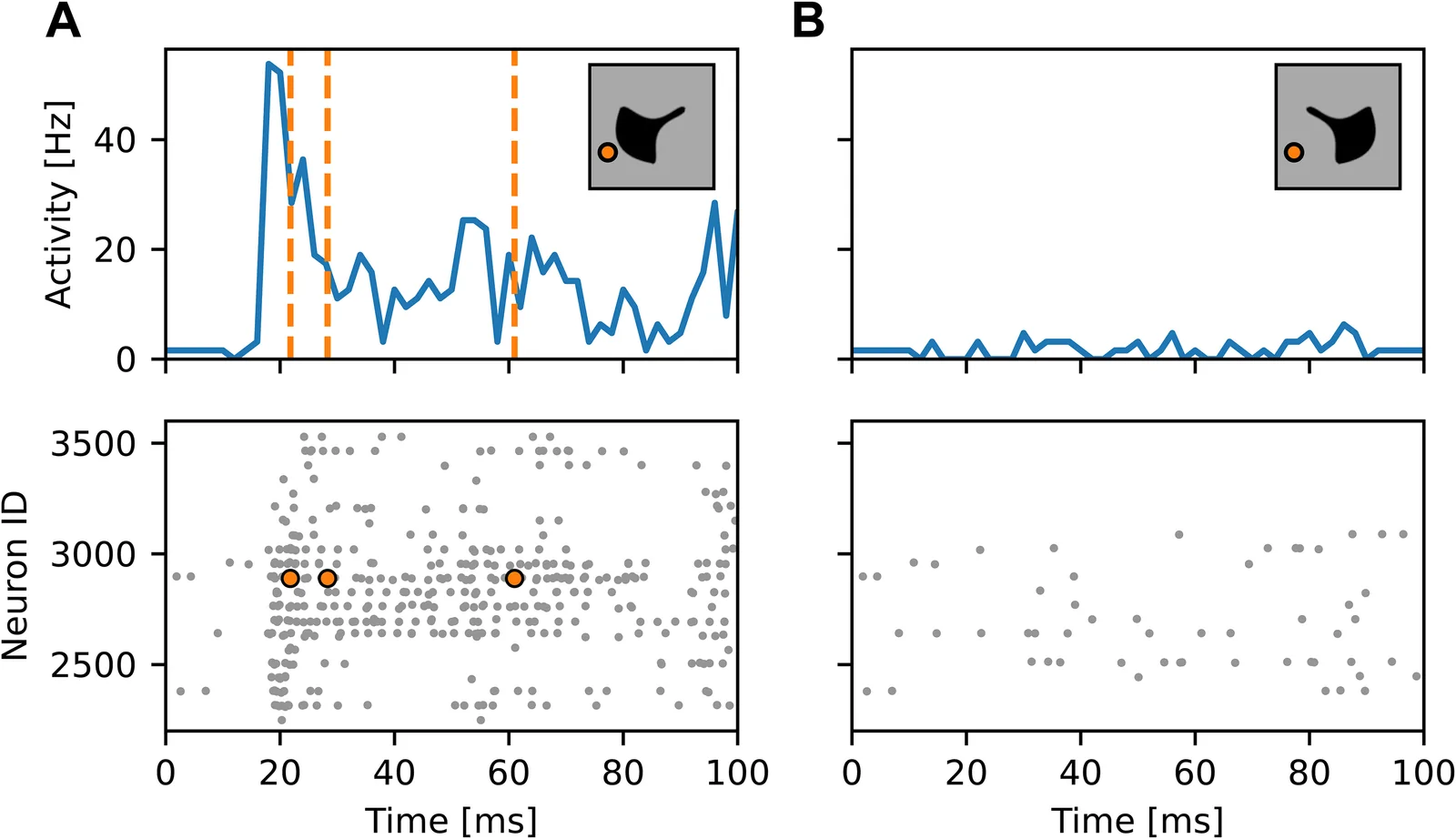
Neuronal firing activity in response to distinct three-sided shapes (N3P2). This figure depicts the firing activity of a population of excitatory neurons localised to a small region within the second-last network layer (L3). This region is centred around neuron #2889 (marked in orange), where this neuron is the first to activate as part of a PNG that selectively responds to shapes possessing a left-convex boundary element. The PNG activation times are indicated by the orange vertical dashed lines. The top panels show the localised population activity, while the bottom panels display their associated spike rasters for the first 100 ms following the presentation of the shape (inset). **(A)** Population response to a shape possessing the preferred left-convex boundary element. **(B)** Population response to a shape lacking a left-convex boundary element.

Fig 21 depicts the localised firing activity of a neuronal population, centred around an excitatory neuron #2889 in L3 that is the first to activate as part of a PNG that selectively responds to shapes with a left-convex boundary element (F1 score ≈1), where this PNG structurally conforms to a three-neuron HFB circuit. Panel A shows that this neuronal population exhibits a strong and rapid response to the presentation of a shape with a left-convex boundary element. The population activity peaks shortly after the shape is presented, with high firing rates that coincide with the initial activation of the PNG. This is evident in both the activity plot and the dense spiking activity in the spike raster, highlighting the tight temporal coupling between early PNG activations and surrounding neuronal activity. In contrast, Panel B demonstrates minimal firing activity when the same neuronal population is presented with a shape lacking the preferred left-convex boundary element. The low levels of neuronal firing observed in both the activity plot and the spike raster indicate the specificity of the population’s response to the preferred shape feature, and by extension, that of the PNG.

Visual inspection of spike rasters and application of spectral analysis to the population activity traces did not reveal sustained oscillations, indicating that the temporal structure of the activity lacks persistent periodic components. The absence of continuous sinusoidal rhythms suggests that the network’s dynamics are dominated by rapid, transient waves of excitatory activity counteracted by inhibitory responses, rather than ongoing oscillatory behaviour. This observation implies that the polychronous patterns observed in this network are not rhythm-mediated, unlike those observed in networks exhibiting strong oscillations [21].

In summary, the clear difference in firing activity between the preferred and non-preferred shapes underscores the network’s ability to selectively and temporally encode specific shape features. The alignment of early PNG activation times with peak population activity suggests that the network leverages these early activations to drive selective responses to relevant shape features.

#### Robustness to key model parameters.

We assessed the robustness of both feature selectivity and polychronization to key parameter choices by sweeping (i) the strength of inhibitory competition (I→E), (ii) the maximum excitatory conduction delay, and (iii) the learning rate. Across these sweeps, the ranked information profiles, the number of informative output neurons, and the detected PNG counts varied smoothly around the default regime (see S10 Fig), indicating that our main conclusions do not rely on finely tuned parameter settings.

## Discussion

In this article, we have explored how neuronal representations of object shape may develop within the primate ventral visual pathway through simulations of biologically detailed SNN models exposed to 2D shapes. This work advances a previous related modelling study [7], by transitioning from a *rate-coded* neural network model to one that incorporates the precise firing times of individual spikes for increased realism. This has enabled us to examine how a hierarchy of shape features at different spatial scales develops and the emergence of hierarchical feature binding between these features through polychronization [1,2,21].

The studied SNN models included bottom-up, top-down, and lateral synaptic connections, with a realistic hierarchical architecture approximating four key processing areas in the ventral visual pathway: V2, V4, TEO and posterior TE [8,9]. Additionally, the axonal conduction delays of synaptic connections were randomised in accordance with experimental data, which has been implicated in supporting the emergence of regularly repeating spatiotemporal patterns, also referred to as PNGs [21], and hypothesised to encode the hierarchical relationships between visual features represented at different spatial scales [1,2].

Regarding network training, we extended the methods of Eguchi and Isbister et al. [1,2] to a more biologically relevant test case by exposing the simulated SNNs to more ecologically realistic datasets comprised of many different 2D object shapes. These shapes were configured using distinct boundary contour elements, with either a convex or concave conformation on each object side. Through network self-organisation via STDP, the objective was to identify groups of shape-boundary selective neurons in the higher layers that align with the response characteristics of neurons in the primate visual system [8–10]. The main hypotheses and outcomes are now discussed.

### STDP facilitates the development of feature selective neurons

STDP is biologically-observed from experiments demonstrating mechanisms of synaptic plasticity, such as those examined in [28,29], and is based on the relative spike-timing differences between paired pre- and postsynaptic neurons. In its common implementation, when a presynaptic neuron fires just before a postsynaptic neuron it leads to LTP of the synaptic strength, while reversed spike-timing causes LTD. In this paper, STDP is used in the context of unsupervised learning, and can be interpreted as maximising the likelihood of generating observed sequences of precisely-timed postsynaptic spikes [49]. This process effectively trains a neuron to learn and encode the statistical regularities of presented spatiotemporal patterns [50].

By applying a Hebbian learning rule akin to STDP, and through repeated exposure of an SNN to a large collection of 2D object shapes, it has been found that neurons learn to represent regularly occurring primitive visual features, such as localised boundary contour elements, through a process termed *statistical decoupling* [7]. This is a process whereby competitive learning within the network forces higher layer neurons to learn to respond to individual primitive features rather than the combinations of features on which the network is trained.

Our present study similarly demonstrated statistical decoupling in the SNN models when exposed to large collections of 2D shapes, constructed from a common alphabet of boundary feature primitives, where synaptic weights were modified by STDP to drive unsupervised competitive learning. As a result, neurons in the final layer emerged that selectively responded to specific boundary contour elements, such as left-side convexity, indicating the network’s adaptation to the statistical regularities in the visual objects. This outcome was further validated through single-cell information analysis, which identified subsets of neurons collectively capable of perfectly discriminating between 2D shapes based on their preferred boundary contour features. This pattern of selective activation is comparable to the response properties observed in area V4 neurons within the primate ventral visual pathway, which are known to encode the conformations of localised boundary contour elements that constitute whole object shapes [8].

Incorporating cell-rate adaptation was necessary to maintain homeostatic firing activity during visually-guided learning. Without this mechanism, the network exhibited a tendency to overlearn, resulting in excitatory neurons firing at biologically unrealistic rates, exceeding 100 Hz. To support the emergence of PNGs, we implemented an ‘additive’ variant of STDP rather than a ‘multiplicative’ one [44,45], following the approach used in previous studies on polychronization [21].

### Neurons in higher layers respond selectively to shape features of increasing spatial scale

The primate ventral visual pathway processes visual information through a hierarchical series of cortical areas, progressively representing features of increasing complexity and spatial scale. Accordingly, we designed a hierarchical SNN model with a feedforward synaptic fan-in radius that increased in size across ascending layers [1,7]. In this way, early-level neurons (layers L1 and L2) had more localised spatial coverage over the input retinal space, whereas higher-level neurons (L3 and L4) covered a much broader region.

With this architecture, the trained SNN models resulted in a hierarchy of visual features being learned at increasing spatial scales. While early neurons encoded small, localised segments of particular boundary elements in their activations, higher neurons were capable of representing the entire curvature of a boundary element. This was evidenced by applying traceback analysis to an SNN exposed to four-sided shapes, with respect to an informative L4 neuron that received convergent inputs from several L3 neurons with narrower receptive fields. While the L3 neurons individually exhibited a marginal preference for a left-side convex boundary element, the L4 neuron combined these inputs to form a more coherent representation that encompassed the entire feature.

In this study, we did not observe higher-level neurons (L3 and L4) that selectively responded to combinations of local boundary contour elements or whole object shapes, as would be expected based on neurophysiological observations of neurons in late-stage areas TEO and posterior TE of the primate ventral visual pathway [10]. Rather, our simulations primarily reproduced the intermediate response properties of neurons in area V4, which encode single boundary contour elements [8].

Our results demonstrated a representational bias towards convex, as opposed to concave, boundary contour elements, which was apparent both before and after network training (Figs 9B, S1, S3, and S4). We attribute this asymmetry to the geometric differences between the two features, together with the network’s lateral competition. A convex element protrudes into empty space and is therefore cleanly isolated, eliciting a strong and consistent response with minimal interference from the object’s other elements. By contrast, a concave element indents into the object interior and is embedded among the surrounding contour elements, so that its oriented response is less isolated and is accompanied by competing activity from these neighbouring elements; this activity drives topologically-aligned excitatory neurons that, in turn, dampen the nearby response via lateral inhibition, a mechanism that is fixed from initialisation and therefore active even before learning. This account is consistent with experimental studies reporting a perceptual dominance of convexity in macaque area V4 [8,51], in which the bias has been attributed to surround inhibition, analogous to the lateral inhibition in our model, acting on concave features that are inherently embedded within the surrounding object contour.

Scaling up the network to a greater number of layers, wider fan-in radii, and increasing the density of synaptic projections [2], may help to produce representations of combinations of boundary contour elements and whole object shapes. Moreover, incorporating an invariance learning mechanism should confer network generalisation [52,53]. This would enable higher-level neurons to generalise across shape transformations, such as positional changes, thereby promoting more stable representations of boundary contour elements that regularly co-occur. Such improvements could support the network’s ability to recognise whole objects or specific combinations of local features, as found with VisNet [7].

Other differences between the rate-based VisNet model [7] and the present SNN model may account for the former being more able to selectively respond to whole object shapes. Firstly, the SNN reliance on STDP for unsupervised competitive learning. Secondly, there is no clear weight vector renormalisation mechanism that can be implemented in a biologically plausible manner.

### PNGs form circuits which encode hierarchical feature binding relationships

The binding problem in neuroscience refers to the challenge of how the brain integrates distinct features of a visual scene, such as colour, shape, and motion, into a coherent perception of objects. A number of mechanistic explanations for this phenomenon have been put forth, including feature integration theory [15] and binding by synchrony [16,52,53].

Recent work has suggested that feature binding may be supported at the level of single neurons through nonlinear dendritic integration [5,6]. In this view, dendritic compartments can act as independent computational subunits, and the mode of synaptic summation (e.g., sublinear versus supralinear) determines whether binding is best supported by globally scattered versus locally clustered synaptic inputs that convey object features. Such mechanisms demonstrate how combination-selective responses and other linearly non-separable computations can arise within a single cell, potentially reducing the burden on downstream circuitry. Our account is complementary to these single-neuron proposals, but targets a different representational requirement: whereas a neuron’s selectivity can reflect a bound combination of synaptic inputs, binding by polychrony is expressed in reproducible timing relationships between feature-selective neurons across hierarchical stages, thereby capturing “part-of” relations between lower- and higher-level features at the circuit level.

Motivated by this representational requirement, and inspired by the findings of [21], the authors of [1,2] hypothesised that polychronization presents a plausible solution to the binding problem, in the form of recurring spatiotemporal patterns (PNGs) that encode hierarchical relationships between primitive visual features represented at different spatial scales.

In contrast to binding by synchrony, which suggests that features are bound together by the synchronised firing activity of encoding neurons, binding by polychrony predicts that PNGs form HFB circuits composed of neurons that fire in a time-locked but asynchronous manner. As illustrated in Fig 2, and in order of their activations, an example of a three-neuron HFB circuit includes two neurons encoding low- and high-level features, followed by a binding neuron that relates these two features together.

The present study aimed to identify PNGs that form such three-neuron HFB circuits, specifically in response to visual features representing a more diverse and ecologically realistic set of 2D object shapes than those considered in previous studies [1,2]. Analysis of inference spike recordings gathered before and after network training revealed that STDP was essential for the emergence of PNGs that structurally correspond to HFB circuits.

Notably, these detections remained robust, despite the randomised timing of individual spikes emitted by input neurons according to a Poisson process. Indeed, we had hypothesised that training SNNs on randomised (Poisson) input spike timings would force the synaptic connections in the higher layers to self-organise using STDP to produce visual representations that are highly robust with respect to image noise after training.

Detailed analysis demonstrated PNGs that fulfilled HFB functionality, consisting of neurons spanning adjacent layers that were individually selective to visual features occurring at different spatial scales. Moreover, this analysis revealed a particular high-level (L4) feature neuron representing an entire contour of a boundary element that combined the narrower boundary representations of two low-level (L3) feature neurons. In this instance, a distinct HFB was associated with each of these pairwise associations, with a different binding neuron linking the low- and high-level features together.

Fig 2 illustrates an example of a classic three-neuron binding circuit, in which a binding neuron encodes the hierarchical binding relationship between a lower-level feature neuron in a lower layer and a higher-level feature neuron in a higher layer. However, the SNN model presented here mimics the known synaptic connectivity of the visual cortex by including both bottom-up and top-down connections between layers, as well as lateral connections within layers. Thus, the visual processing is not purely bottom-up (feedforward). In this case, the kind of binding circuit shown in Fig 2 could also operate as part of top-down processing, representing the fact that a neuron in a higher layer is participating in driving a neuron in a lower layer. Alternatively, this kind of binding circuit could also function within a single layer, indicating that one feature neuron participates in driving another feature neuron within the same layer. Indeed, because of the presence of extensive recurrent connections between excitatory neurons within individual layers, it is possible for hierarchical visual processing to operate within each layer. In each of these cases, the binding neurons essentially represent the causal relations between different features or feature neurons. This notion is in some sense consistent with the proposal that STDP is a learning mechanism that strengthens synaptic connections between neurons based on their causal relations. That is, STDP strengthens a synaptic connection *if and-only-if* the presynaptic neuron is participating in driving the postsynaptic neuron. However, STDP alone will not enable the brain to explicitly represent and utilise information about these causal relations to enrich perception and guide behaviour. To achieve this, the network must also learn to explicitly represent these causal relations between features or feature neurons through the emergence of additional ‘binding neurons’ as demonstrated in this paper. We find that the emergence of such binding neurons is ubiquitous wherever feature neurons participate in driving each other within the network.

Taken together, these results support the notion that binding by polychrony is a biologically plausible alternative to existing hypotheses of feature binding. In particular, it addresses a key shortcoming inherent in binding by synchrony, namely the latter’s reliance on homogeneous axonal conduction delays. In biological neural circuits, the transmission delay in spike propagation between connected neurons is typically non-uniform, varying from between one and several tens of milliseconds [21]. While synchrony is degraded by heterogeneous conduction delays, polychronization leverages this variability to form diverse PNGs capable of encoding a wide range of visual features and their hierarchical relationships.

The experimental evidence supporting binding by synchrony is limited [54], and its suggestion that visual scenes are decomposed into distinct object regions [17] fails to account for the rich hierarchical binding relationships between object features spanning different spatial scales. Here we have advanced an alternative hypothesis to address this issue that relies on binding by polychrony, in which heterogeneous axonal conduction delays facilitate the formation of PNGs, allowing for the encoding of complex, multi-scale feature relationships within the visual scene.

### Reuse of binding circuits reflects the capacity of a polychronous code

A notable property of the binding circuits identified here is that the neurons occupying each role, low-level, high-level and binding, are reused across many circuits rather than being dedicated to a single one. For the binding neuron this reuse is unavoidable, since a layer contains far fewer neurons than there are circuits to support, so each binding neuron participates in several circuits and the population of distinct binding neurons is much smaller than the set of binding relationships it represents. Rather than maintaining a separate copy of the lower-level representation at each higher level, the network thereby encodes far more relationships than it has neurons, which is the defining representational capacity of a polychronous code [21]. This is possible because each relationship is carried by the time-locked firing of its constituent neurons rather than by the identity of any one of them: a binding neuron fires whenever its low- and high-level partners arrive together at matched conduction delays, so the same neuron can participate in circuits that encode different feature combinations, with the particular combination specified by which partners co-fire and with what relative timing. A larger repertoire of low-level features is accommodated in the same way, since a single binding neuron can mediate several low-to-high pairings without requiring a correspondingly larger dedicated population.

Although the binding relationship is in this sense distributed across the firing pattern, the feature that a selective circuit encodes is anchored to its informative low-level constituent, which almost always sets the feature of the selective circuits it joins and is in this sense the principal determinant of what they represent. The high-level and binding neurons of a circuit are individually weaker predictors of its feature: the binding neuron because its firing as a coincidence detector depends on the context of its partners, and the high-level neuron because its larger receptive field and more integrative tuning render it less sharply selective for any single boundary element than the spatially localised, first-firing low-level neuron. Because the role a neuron plays is set by its context within a circuit rather than being intrinsic to it, this organisation extends through the hierarchy: a neuron that is the high-level feature neuron of one circuit is the low-level neuron of a circuit one layer above, where, if that circuit is selective, it in turn sets the feature. The feature a selective circuit represents is therefore carried by its informative low-level constituent, while the relationship binding it to the higher-level feature is carried by the time-locked pattern as a whole.

This reuse operates within the regulatory context of the lateral inhibition implemented throughout the network’s layers, which enforces sparseness and competition between feature neurons and thereby prevents circuits from proliferating uncontrollably, driving the network instead towards its most reliable feature associations. The three-neuron motif examined here is intentionally minimal, selected for the clear characterisation it affords of the relationship between polychronization and the feature hierarchy, and its binding functionality is expected to extend to larger circuits: complex object representations would be encoded by nested arrangements of such motifs, in which a high-level feature is bound to several lower-level features through binding neurons that are themselves reused across the wider ensemble. Any large-scale binding ensemble can in principle be decomposed into these constituent three-neuron motifs, operating in parallel or in sequence to bind features across increasing levels of abstraction. Represented in this way, the cost of binding an entire object scales with the number of feature relationships it contains rather than requiring a dedicated neuron for each association, allowing the representation of more complex objects without a corresponding growth in the binding population.

### Spike-based dynamics support robust selectivity

During testing, the firing rates of the input neurons representing the shape images were set according to the outputs of Gabor filters, which mimicked the responses of edge- and bar-detecting simple cells in cortical visual area V1. However, the precise spike timings of these input neurons were randomised according to a Poisson distribution. Despite this substantial variability (jitter) in input spike timings, neurons in the final layer continued to exhibit selective responses to their preferred visual features at multiple spatial scales. Several factors contribute to this robust performance, which are elaborated on as follows.

During training, the Poisson-induced variability in the timings of individual spikes emitted by input neurons, combined with biologically realistic STDP, drives the emergence of synaptic connectivity patterns throughout the network; this process selectively enhances synaptic connections with particular axonal conduction delays while pruning others, consequently endowing the overall network with high robustness with respect to noise in the timings of input spikes that are emitted during subsequent testing. Thus, biologically realistic STDP promotes the emergence of robust visual representations within SNNs.

Moreover, when STDP is implemented in the context of unsupervised competitive learning, it drives the emergence of neurons that encode HFB relationships between visual features existing at different spatial scales within the SNN. These ‘binding neurons’ enable the network to represent the spatial structure of object shapes in terms of the hierarchical relationships between entire shapes and their constituent parts, e.g., the boundary contour elements. Explicitly representing this spatial structure of object shapes provides a more robust foundation for object recognition than current deep neural networks that do not explicitly preserve and represent information about the hierarchical binding relationships between features at different spatial scales across network layers.

The SNN model presented here mimics the known synaptic connectivity of the visual cortex by including both bottom-up and top-down connections between layers, as well as including lateral connections within layers. As a result, the visual processing is not strictly bottom-up (feedforward). Instead, this kind of connectivity gives rise to *attractor* dynamics spanning all of the network layers; visual signals continue to propagate upwards, downwards, and laterally until the network falls into an asymptotically stable attractor state. Such a final state maximises the consistency between the lower-level constituent visual features, such as boundary contour elements, higher-level visual features, such as whole object shapes, and the HFB representations that encode the relationships between these features. In this way, the attractor dynamics help to produce the most consistent and coherent representation of the visual scene, including whole object shapes, in terms of the hierarchical relations between visual features at different spatial scales. The HFB binding neurons are likely to play a key role in these network-wide attractor dynamics.

Unsupervised competitive learning drives neurons within the network to learn to represent the *statistically significant* visual features that tend to reoccur regularly across (e.g., natural) images. The kinds of visual features that emerged within our SNN simulations with STDP were predominantly boundary contour elements spanning multiple spatial scales, including binding neurons encoding their hierarchical relationships. We propose that these natural, statistically significant reoccurring features establish a robust basis set for object representations, and that is resilient against various forms of noise and natural object transformations. This is contrasted with deep neural networks, trained with supervised learning methods, that often learn more abstract features within their intermediate layers. Such abstract features may give rise to arbitrary and unnatural decision boundaries that limit robustness to image noise and natural object transformations. The problem of ‘adversarial noise’ in deep neural networks, in which minimal pixel perturbations can radically alter image classifications (e.g., misclassifying a school bus as an ostrich [55]), may be related to this phenomenon.

### Limitations and scope

Our modelling choices intentionally prioritised experimental control over stimulus complexity. We trained and tested the network on restricted sets of 2D object shapes with binary boundary conformations, presented at a fixed, centred retinal location. This design was chosen to directly test the statistical decoupling argument under unsupervised competitive learning and to allow feature selectivity and HFB-structured PNGs to be systematically quantified. The trade-off is that these stimuli omit texture and clutter, are static, and they do not assess invariance to common transformations (e.g., translation, rotation, or scale). Extending the analysis to translated shapes and temporally structured visual inputs, and to more naturalistic image ensembles, is therefore an important next step (see Future work).

In addition, the model does not explicitly simulate cortical area V1. Instead, V1-like orientation selectivity is approximated by a fixed bank of Gabor filters whose outputs drive Poisson spike trains in the input layer. This approach follows prior SNN modelling studies [1,2] and provides a practical, spike-based encoding of continuous-valued visual inputs. However, it omits V1 microcircuit dynamics, including recurrent interactions and the emergence of complex-cell-like invariances [24], which could influence the statistics of the signals propagated to higher areas and hence the conditions under which downstream STDP and polychronization develop.

Finally, two further limitations concern the binding circuits themselves. First, our analysis shows that the bound feature is carried by the time-locked firing of a circuit and can be read from it, but we do not model an explicit downstream neuron that performs this read-out to make the binding available to subsequent processing; demonstrating such a mechanism is an important direction for future work. Second, the boundary-element representations characterised here correspond to an intermediate, V4-like stage of the ventral hierarchy rather than the object-level conjunctions associated with areas TEO and TE [10], and a fuller account of how higher-order conjunctive tuning develops over more abstract feature combinations would require larger and deeper networks than those studied here.

### Implications for neuroscience and AI

The results in this study provide evidence that the mechanism demonstrated here can support feature binding, which is a significant step towards addressing a problem that has puzzled neuroscientists for decades. This mechanistic account can help guide experimentalists towards identifying analogous circuits *in vivo*, and testing whether related dynamics contribute to feature binding in the brain. Obtaining such experimental evidence would help to identify canonical circuits for hierarchical feature binding and transform our understanding of perception.

However, even in the absence of experimental evidence, the mechanism demonstrated has utility in an engineering setting as it enables the development of information-rich representations of the data received through sensing. Implementing this mechanism in computer vision could produce visual field representations closer to biological vision, specifically by identifying feature-to-feature relationships across all spatial scales. Theoretically, this may influence a system’s resistance to adversarial inputs compared to CNNs and transformer architectures in, for example, object classification tasks. This is because the network could learn to exploit the predictive information carried by binding neurons for class prediction. Both CNNs and vision transformers show vulnerability to adversarial attacks such as human-imperceptible pixel/token perturbations resulting in object misclassification [56,57]. Although the precise susceptibility of such a system remains to be tested, it is possible that relying solely on binding information rather than texture or shape information to classify objects would alter how models respond to such attacks. Future work could explore whether models that use hierarchical feature binding for feature extraction display greater robustness to the types of noise observed in our simulations (Figs 11 and 12).

**Fig 22:**
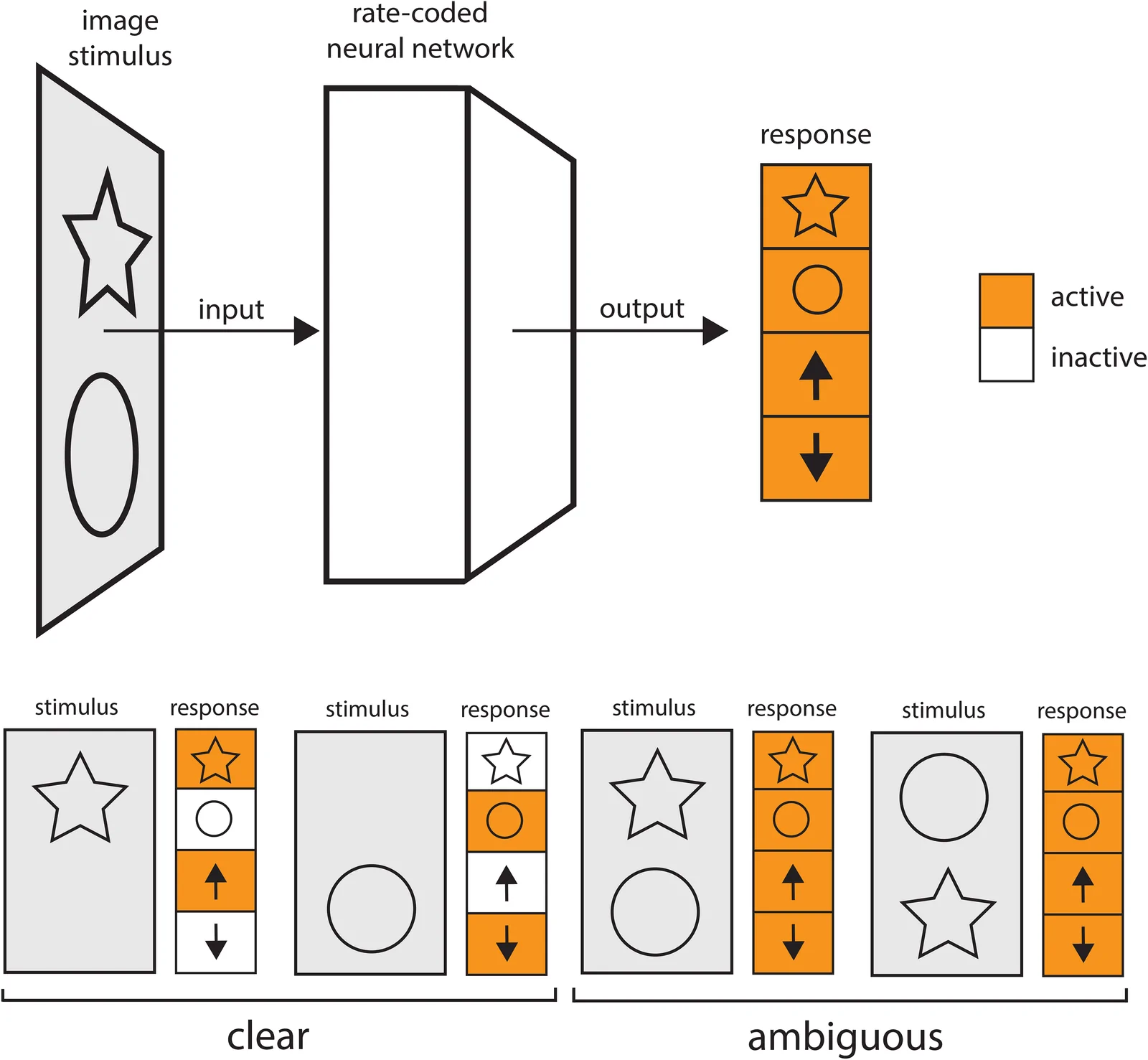
Illustration of the superposition catastrophe. When different objects are present in both positions, the location of each object is ambiguous because the information capacity of the network is not sufficient for representing this.

The reason for the fragility of modern computer vision architectures such as convolutional neural networks (CNNs) and vision transformers can be understood by their inability to solve the feature binding problem. This failure is clearly explained by the authors of [14], and the underlying weakness is illustrated in a famous toy example given in [13]. For this example, consider the model for visual recognition shown in Fig 22, which can derive four propositions and represent each of them with an output neuron. Two neurons recognise objects: either a star or a circle, invariant to position, and two neurons indicate the position invariant to object form. As illustrated, the network responds well when a single object is shown, but not when two objects are presented simultaneously. For example, if all four output neurons become active, it is not clear which shape is at which location. This problem is an example of a wider phenomenon known as the superposition catastrophe [14] and results from the model’s inability to solve the binding problem since it does not have any means of binding the location to the object type. This failure occurs because traditional neural networks are deficient in representing hierarchical feature binding information.

For instance, in CNN architectures, downstream pooling operations are applied that aggregate detected features, consequently discarding precise location information. This renders CNNs vulnerable to adversarial examples where necessary features are present but spatially rearranged. Such images might still be misclassified by the network as the intended object. This susceptibility is often illustrated by the ‘Picasso problem,’ where despite the distorted spatial arrangement of features, the network erroneously recognises the image as the correct object. Some work has been carried out in an attempt to improve the ability of deep neural networks to represent hierarchical information, the most notable example being the development of capsule networks. Capsule networks are CNNs which do not include pooling layers, therefore preserving more spatial information as layers of the network are ascended, and include ‘capsules’ of neurons which encode features such as the position and orientation for specific types of object [58]. However, the kinds of hierarchical relationship that these networks can encode are still limited by the type of objects they are trained on, and they remain susceptible to other types of adversarial inputs. For example, CNNs respond to texture over shape features during classification. Consequently, adversarial inputs can be created by modifying texture magnitudes to trick the CNN while remaining recognisable to humans. Other attempts to address the inability of such artificial neural networks to represent hierarchical information include training them to perform hierarchical segmentation through multi-class classification with hierarchically defined classes [59]. However, the segmentation network must be trained in a supervised manner. In contrast, hierarchical feature binding is capable of learning hierarchical relationships in an unsupervised manner.

Vision transformers have become a popular alternative to CNNs for computer vision tasks, but these have been demonstrated to show vulnerability to adversarial attacks and in some cases are even more susceptible than CNNs [57]. It could be argued that the self-attention mechanism in transformers performs a function analogous to binding, with attention scores encoding which parts of the input are relevant to other parts and different attention heads encoding different kinds of relevance. While weights for a given attention head may encode object membership, and self-supervised transformers can learn segmentation masks [59], these representations differ from HFBs in SNNs in several ways. First, self-attention lacks explicit hierarchical encoding. Although attention layers can be stacked, each layer’s input is already weighted by previous scores, leaving subsequent layers without direct knowledge of prior scores. Secondly, the binding representations in HFBs emerge through unsupervised training, whilst the formation of analogous representations in transformers requires supervised or self-supervised training. Thirdly, in order to learn useful attention scores, transformers require exposure to vast amounts of training data. In contrast, we demonstrate here that HFB representations in SNNs emerge after exposure to relatively few examples and therefore potentially offer a much more efficient method of learning binding representations.

### Future work

A key limitation of the present study is that we did not evaluate transformation invariance (e.g., translation) of boundary-contour responses, despite this being a known property of neurons along the ventral pathway [8,9]. In principle, such invariance could be learned through biologically plausible mechanisms such as *continuous transformation learning* or *trace learning* [7,52].

Continuous transformation (CT) learning relies upon the spatial continuity of continuously transforming visual stimuli and a Hebbian learning rule to associate together successive transformations of a stimulus, such as spatial translations, rotations or scalings [60]. When a stimulus is presented to a network via presynaptic neuronal activity, it activates one or more postsynaptic neurons, leading to the strengthening of synapses between these neurons via Hebbian learning. If a subsequently transformed version of this stimulus shares sufficient similarity (overlap) with the original, it activates the same postsynaptic neuron(s), further strengthening their synaptic connections to the presynaptic neurons representing the current transformation. Hence, this iterative process can integrate a sequence of incremental transformations, mapping them onto identical output neurons. Given that similar images are more likely to represent transformations of the same object rather than disparate entities, this CT learning mechanism provides an explanation for how transformation-invariant representations may develop within the ventral visual system.

By contrast, trace learning takes advantage of the temporal continuity of objects in the world to learn transformation-invariant representations [61]. The mechanism relies upon the intuition that over short time scales, successive images are more likely to be transformations of the same object rather than different objects. In a rate-coded neural network, the trace learning rule [30,61] incorporates a temporal memory trace of the previous neural activity into a Hebbian learning rule. The memory trace in the learning rule ensures that afferent synapses onto a recently active postsynaptic neuron continue to be potentiated when successive transformed stimuli are presented. Through further synaptic modifications using a trace learning rule, successive transforms of an object may become associated together onto the same output cells leading to transformation-invariant neurons. In an SNN, trace learning operates slightly differently. In this case, a long time constant for the synaptic conductances keeps the postsynaptic neuron firing longer. This enables subsequent transforms of an object to be associated onto the same output cell [52].

Both CT learning and trace learning are biologically plausible unsupervised competitive learning mechanisms that operate simultaneously within every layer of a hierarchical neural network architecture, driving the development of a hierarchy of transform invariant features at every spatial scale. As shown in [52], populations of neurons in the higher layers of SNNs trained in an unsupervised and competitive manner develop transform-invariant responses to input stimuli under both CT learning and trace learning mechanisms. By extension of this phenomenon, we predict that both feature selective neurons and binding neurons existing at every level of our studied hierarchical SNN should be capable of developing transformation-invariant responses if either of these two learning mechanisms are implemented. In further support of this, such an effect has been demonstrated for boundary contour feature neurons trained via trace learning for rate-based neural networks, with a similar architecture to our own [7].

These learning rules, implemented within a biologically realistic SNN model, drive the emergence of a hierarchy of transformation-invariant features at every spatial scale. This outcome is particularly powerful, as it facilitates ‘one-shot’ learning, whereby the model can generalise to novel transforms of an object not previously encountered, given a single exposure to just one transformation (e.g., view) of the object during training. The ability of the network to perform one-shot learning rests on the emergence of a hierarchy of transform-invariant feature primitives at every spatial scale through successive layers; this hierarchy develops during initial training with an invariance learning mechanism such as CT or trace learning. Any new object is encoded using this hierarchy of transform-invariant feature primitives. Since the constituent feature primitives are already transform-invariant, the network can immediately generalise to novel views of an object. Such generalisations to novel transformations have been demonstrated for both position invariance [62] and view invariance [60,63]. This ability to perform one-shot learning marks a significant departure from the learning paradigms prevalent in most artificial neural networks and highlights a distinctive aspect of learning in biological brains. The exploration of biologically realistic neural network architectures and learning rules that facilitate this phenomenon could indicate the underlying mechanisms enabling such capabilities in the brain.

A further direction for future work is to examine how polychronization and hierarchical feature binding scale with network depth and neuronal density, and how they behave under greater structural heterogeneity. While the present study uses largely homogeneous neuron and synapse parameters for computational tractability (Table 1), hierarchical organisation is still introduced by the layer-dependent increase in fan-in (receptive field size), and binding emerges through local STDP dynamics acting on heterogeneous axonal delays together with competitive E/I interactions. On this basis, we speculate that introducing biologically inspired heterogeneity (e.g., varying neuron numbers, connection densities, or time constants across layers) would be expected to modulate where and at what spatial scales PNGs and binding neurons emerge, rather than eliminate the underlying delay-matched binding mechanism, and may increase the diversity of potential polychronous circuits. Similarly, increasing depth or density should expand the reservoir of candidate delay-matched pathways available for selection. We have not yet performed a systematic simulation of these larger and more heterogeneous regimes because PNG detection is computationally expensive and the search space grows rapidly with network size; a comprehensive exploration, together with more efficient spike-pattern discovery methods, is left for future work.

A key aspect of future research using this model should focus on exploring the holographic principle within the network. This principle posits that information about features at every spatial scale, including HFB relations, is retained in the network’s uppermost layer: this retention allows a large amount of information to be conveyed and preserved at higher levels for subsequent retrieval, particularly in regions such as the prefrontal cortex (PFC) for advanced cognitive processes. The holographic principle has been substantiated by the emergence of border ownership cells in every layer of a rate-coded hierarchical model [64]. This was extended to spiking networks in [1] while [2] explains how this principle solves the feature binding problem. Such findings underscore the principle’s potential role in biological systems and its implications for advancing more general intelligence. It is evident that artificial neural networks currently do not capture the same semantically rich representations of the world that the biological brain does, possibly due to the absence of mechanisms akin to HFB and the holographic principle.

In addition to their functional advantages, polychrony-based computations are intrinsically energy-efficient: spikes are emitted sparsely, computation is event-driven, and synapses are updated only when pre- and postsynaptic spikes arrive. A recent analysis [65] shows that SNNs can compete with efficient ANN implementations when spike activity is sufficiently sparse. This property is particularly relevant for low-power edge-AI devices with on-chip learning.

The hierarchical feature binding mechanism demonstrated in this study holds promise for significantly enhancing artificial intelligence systems, particularly within the field of computer vision. However, applying this mechanism to real-world scenarios presents certain challenges. Although useful information about object identity and location can be extracted from neuronal firing rates alone, leveraging HFB relationships, as encoded by PNGs, enables the capture of more semantically complete information. Specifically, they reveal the precise sets of visual features responsible for driving these neuronal responses, including their hierarchical relationships. Therefore, developing efficient algorithms that can rapidly identify PNGs and decode their feature representations is crucial for tasks requiring a deeper understanding of feature interactions, such as detailed object recognition and localisation. This advancement is essential for the real-time utility of such networks in more complex applications. Moreover, future investigations are essential to determine the most salient hierarchical feature relationships, which are likely to vary considerably across different visual processing tasks. The potential applications for these types of networks are substantial, especially when integrated with traditional neural network models such as CNNs or transformers to create hybrid networks. Such integration aims to combine the best attributes of both systems.

## Supporting information

S1 FigSingle-neuron information analysis for concave boundary contour elements.(A) Rank-ordered stimulus-specific information conveyed by each final layer excitatory neuron regarding concavity (concave vs. convex). For each neuron, $ℐ(s,\vec{R})$ was computed separately for concavities on each object side and the *maximum* value across sides is plotted (i.e., the side for which the neuron was most informative). Only neurons that preferentially increased their firing for a concave vs. convex contour on the corresponding side were considered concave-selective for this analysis. Curves are shown for untrained (pre-learning) and trained networks, for both datasets (N3P2 and N4P2) and for the three network architectures (FF, FF + LAT, FF + LAT + FB), matching the analysis in Fig 9, and averaged across three independent trials. (B) Number of concave-informative neurons exceeding an information threshold of 2/3 bits about concavity at their preferred side, shown as the mean ± s.e.m. across three trials, with the corresponding proportions of the L4 excitatory population annotated above each bar.(PDF)

S2 FigSelf-organised feature maps in the output layer representing specific concave boundary elements after network training.These contour plots correspond to the distinct clusters of excitatory neurons in L4 which carry at least 2/3 bits of information regarding their preferred concave boundary elements. This 64×64 output layer is topologically aligned with the 128×128 sized images, with wider spacing between output neurons to maintain alignment. Left: Information map for neurons selective to concave boundary elements in the N3P2 dataset. Right: Information map for neurons selective to concave boundary elements in the N4P2 dataset.(PDF)

S3 FigSide-resolved single-neuron information analysis for N3P2 boundary contour elements.(A) Rank-ordered stimulus-specific information conveyed by final layer excitatory neurons regarding convexity (left column) and concavity (right column), computed separately for each object side (rows). Curves show pre-trained (dotted) and trained (solid) network states, averaged across three independent trials. (B) Number of neurons exceeding an information threshold of 2/3 bits for each side, shown as the mean ± s.e.m. across trials, with the corresponding proportions of the L4 excitatory population annotated above each bar.(PDF)

S4 FigSide-resolved single-neuron information analysis for N4P2 boundary contour elements.Similar design as in S3 Fig. (A) Rank-ordered stimulus-specific information conveyed by final layer excitatory neurons regarding convexity (left column) and concavity (right column), computed separately for each object side (rows). (B) Number of neurons exceeding an information threshold of 2/3 bits for each side, shown as the mean ± s.e.m. across trials.(PDF)

S5 FigStage-wise retention of three-neuron PNGs through the HFB detection pipeline.Bar plots show the number of three-neuron spatiotemporal spike patterns detected by SPADE for a single network (FF + LAT + FB) trained on N3P2, grouped by the layer *L* of the second-firing neuron, for motifs with the target hierarchical structure [L−1,L,L] (low-level feature neuron in L−1, followed by high-level and binding neurons in *L*). Blue bars (Detected; D) report unconstrained detections that enforce only this layer structure, the prescribed firing order, and the required HFB connectivity, but do *not* impose a minimum synaptic weight and do *not* require delay-aligned spike timing. Orange bars (Eligible; E) show the subset retained after applying the HFB eligibility criteria used throughout the main text, including a minimum synaptic weight ($\Delta g_{ij}\geq 0.5$) on the required feedforward and lateral connections and alignment of observed spike-timing differences with axonal conduction delays within a tolerance of δt=3 ms. Green bars (Significant; S) show the subset of eligible patterns that pass surrogate-based significance testing (*p* < 0.05; 100 surrogates; 5 ms spike-time dithering). The inset box summarises overall retention across layers: *E*/*D* is the fraction of detected patterns satisfying the HFB constraints, *S*/*E* is the fraction of eligible patterns that are statistically significant, and *S*/*D* is the overall retention from detection to significance. For the detected stage (D), counts are the number of *unique* neuron triplets defined by neuron IDs and firing order; for the eligible and significant stages (E/S), repeated detections are merged into unique characteristic PNGs as described in Methods.(PDF)

S6 Fig Representational performance of PNGs with respect to concave boundary elements. Rank-order plots of F1 scores for PNGs detected in the final network layer, evaluated against concave boundary elements from the N3P2 (left) and N4P2 (right) datasets. Networks were trained with either feedforward and lateral connections (FF + LAT) or with added feedback (FF + LAT + FB). By comparison with the convex analysis, PNGs exhibit decreased selectivity for concave features, with a negligible number of PNGs achieving high F1 scores (>0.9) across all conditions. Error bars indicate standard deviation across three independent runs.
(PDF)

S7 FigSensitivity of detected PNG counts to temporal-span and timing-tolerance parameters.All analyses consider three-neuron PNGs that structurally conform to the target HFB motif, and counts are reported by the layer *L* of the second-firing neuron, with the total shown in black. (A) Distribution of PNG temporal spans, defined as the time between the first and last spikes within each detected pattern; the dashed vertical line indicates the mean span, and PNGs are pooled across all layers. (B) Number of retained PNGs as a function of the imposed maximum span used during detection. The default choice used in the main analyses (20 ms) lies in the plateau regime, indicating that the reported PNG counts are not tightly constrained by this parameter. (C) Number of retained PNGs as a function of the delay-matching tolerance δt applied when comparing observed spike-timing differences to the underlying axonal conduction delays. As expected, increasing δt increases the number of admissible PNGs by relaxing the timing constraint.(PDF)

S8 FigPer-neuron participation and role-switching across the three circuit roles.For each of the three circuit roles, low-level (L), high-level (H) and binding (B), this figure quantifies how individual neurons are reused across, and switch roles between, the three-neuron PNGs that structurally conform to HFB circuits. Results are shown for a single representative trial of a network trained on N4P2 with the FF + LAT + FB architecture. (A) Histograms report, for each anchor layer *L* = 2–4, how many neurons in a given role participate in a given number of these PNGs. Each row corresponds to one role, and each column to PNGs anchored at layer *L*, taken to be the layer of the second-firing (high-level) neuron in the [L−1,L,L] triplet motif; the low-level neurons of these PNGs therefore reside in the preceding layer L−1. The x-axis reports the number of distinct PNGs in which a given neuron appears in that role, while the y-axis reports the number of neurons with that participation level (i.e., the number exhibiting that degree of reuse). Dashed vertical lines indicate the mean participation level (μ) across neurons in that role, and *n* is the total number of neurons recruited into that role at that layer. (B) Role co-membership across the three roles, shown as one matrix per anchor layer. Cell (*r*,*c*) is the fraction of neurons occupying role *r* that also occupy role *c* somewhere in their participation, so each matrix is row-conditional and the diagonal is one by construction. Rows and columns follow panel (A): the high-level and binding rows are neurons in the anchor layer, while the low-level row comprises neurons in the layer below. Within a layer, most high-level neurons are also binding neurons in other circuits, and conversely (about two-thirds to three-quarters at every layer). The low-level cells are cross-layer: (*H*,*L*) and (*B*,*L*) give the fraction of anchor-layer neurons that also serve as the low-level neuron of a circuit one layer higher (about 0.84 at layers 2 and 3), and (*L*,*H*) and (*L*,*B*) give the converse for the layer below. The low-level column in the layer-4 matrix is greyed, since a final-layer neuron has no higher layer for which it could be the low-level neuron. Both panels omit the layer-1 anchor layer, since the low-level neuron of a layer-1 circuit is a fixed Poisson input rather than a learned neuron. Together, these results show that reuse and role-switching occur at all three positions of the HFB circuit, consistent with the binding relationships being represented combinatorially by a shared population of reused neurons rather than by a one-to-one allocation of neurons to relationships.(PDF)

S9 Fig Completeness and convex bias of the binding-circuit representation of boundary contour elements. This figure quantifies the completeness of the three-neuron HFB representation by comparing the low-level (L) neurons recruited into binding circuits against the informative low-level population, for both datasets: (A) N3P2 (six boundary contour elements) and (B) N4P2 (eight elements). Networks were trained with the FF + LAT + FB architecture, and counts are the mean across three independent trials. Binding circuits are anchored at the final layer (L4), so their low-level neurons reside in the preceding layer (L3); a low-level neuron is counted as informative if it carries at least 2/3 bits of stimulus-specific information about convex versus concave at its preferred object side. For each (side, conformation) feature, nested bars on a logarithmic axis report the number of distinct informative low-level neurons selective for that feature (the informative pool; lightest bar, labelled Informative, with its count printed above each group) together with the subset of those neurons recruited as the low-level member of at least one binding circuit selective for the same feature, at increasing circuit selectivity: a labelled circuit of any positive F1 score (F1 > 0), or a circuit exceeding an F1 score of 0.5, 0.7 or 0.9. Counting distinct neurons collapses the reuse of low-level neurons across circuits. Features are clustered by conformation, and a feature lacking a bar at a given threshold is one for which no qualifying circuit was detected, so feature coverage can be read directly at each level of selectivity. Coverage of the feature repertoire is complete at moderate selectivity, with every feature represented by a circuit exceeding an F1 score of 0.7, and thins to a sparse, convex-biased subset at the strict F1 > 0.9 criterion.
(PDF)

S10 FigSensitivity of single-neuron selectivity and HFB-structured PNGs to key network parameters.We varied three parameters–lateral inhibition strength ($\lambda^{I\to E}$), the maximum axonal conduction delay ($d_{\max}$), and the STDP learning rate (ρ)–and quantified the effect on both output selectivity and detected PNGs, averaging results across three independent trials. (A) Rank-ordered stimulus-specific information $ℐ(s,\vec{R})$ conveyed by each final-layer (L4) excitatory neuron regarding convexity at its preferred object side (computed per side and reported as the maximum across sides), shown for each parameter setting. (B) Corresponding counts of informative L4 excitatory neurons exceeding an information threshold of 2/3 bits (percentages above bars); error bars indicate s.e.m. across the three trials. (C) Number of detected three-neuron PNGs that structurally conform to the target HFB triplet motif spanning the final two layers; error bars indicate s.e.m. across the three trials. Orange curves/bars mark the default parameter values used throughout the main text. Across all three sweeps, both the ranked information profiles and the summary counts vary smoothly, indicating that output selectivity and HFB-structured PNG detection are robust to moderate changes in these key parameters.(PDF)
