# Supplementary material for: Hierarchical feature binding in a spiking neural network model of the primate ventral visual pathway
Source: PLoS Comput Biol. 2026 Sep 16;22(9):e1014752. doi: 10.1371/journal.pcbi.1014752 (PMC13581216; doi:10.1371/journal.pcbi.1014752)
Supplement: Supplementary file 1 [file pcbi.1014752.s001.pdf]

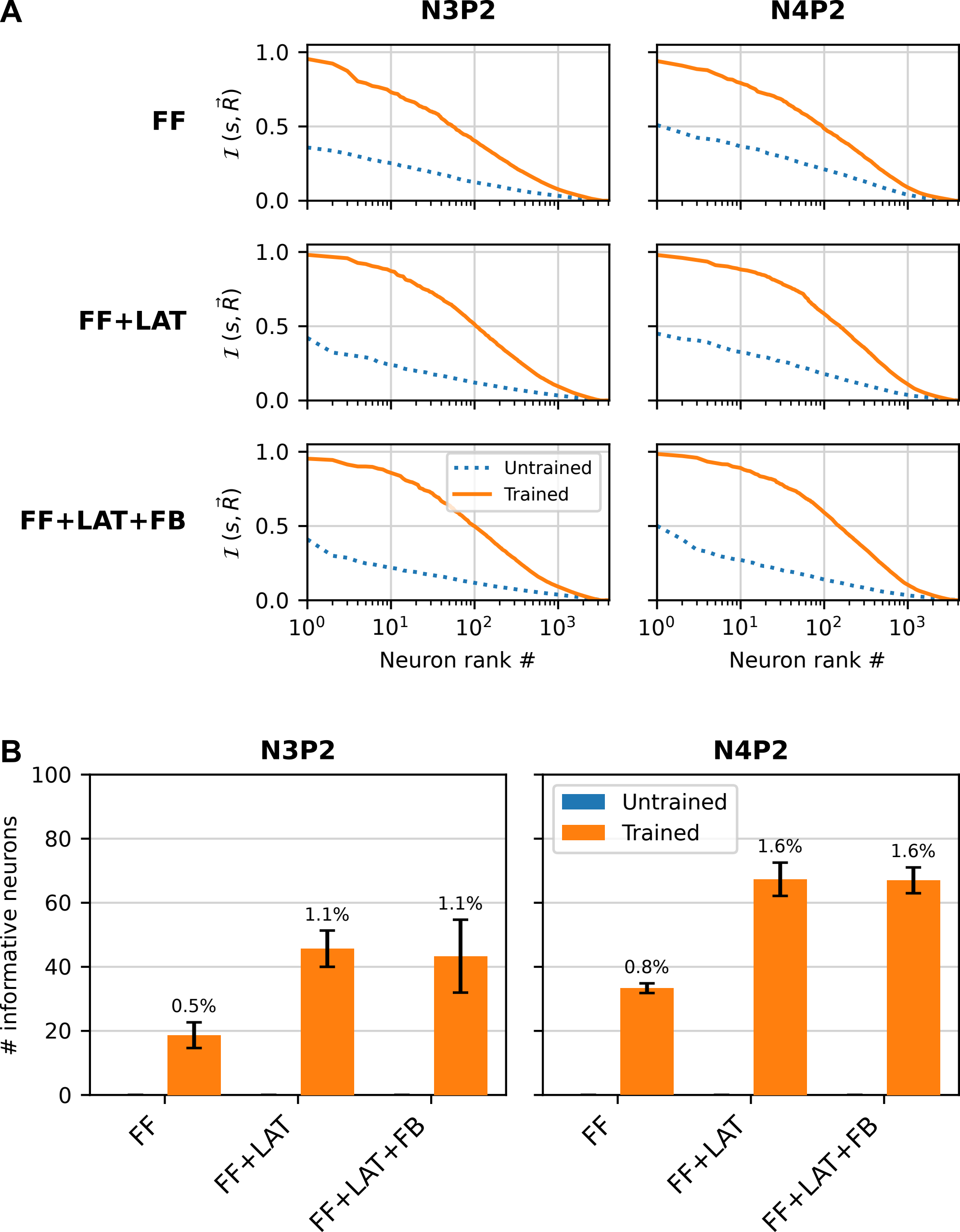
A
N3P2
N4P2
FF
FF+LAT
FF+LAT+FB
$\mathcal{I}\,(s,\vec{R})$
1.0
0.5
0.0
Untrained
Trained
10^0
10^1
10^2
10^3
Neuron rank #
B
N3P2
N4P2
informative neurons
100
80
60
40
20
0
0.5%
1.1%
1.1%
0.8%
1.6%
1.6%
Untrained
Trained
FF
FF+LAT
FF+LAT+FB
