# Supplementary material for: Hierarchical feature binding in a spiking neural network model of the primate ventral visual pathway
Source: PLoS Comput Biol. 2026 Sep 16;22(9):e1014752. doi: 10.1371/journal.pcbi.1014752 (PMC13581216; doi:10.1371/journal.pcbi.1014752)
Supplement: Supplementary file 2 [file pcbi.1014752.s002.pdf]

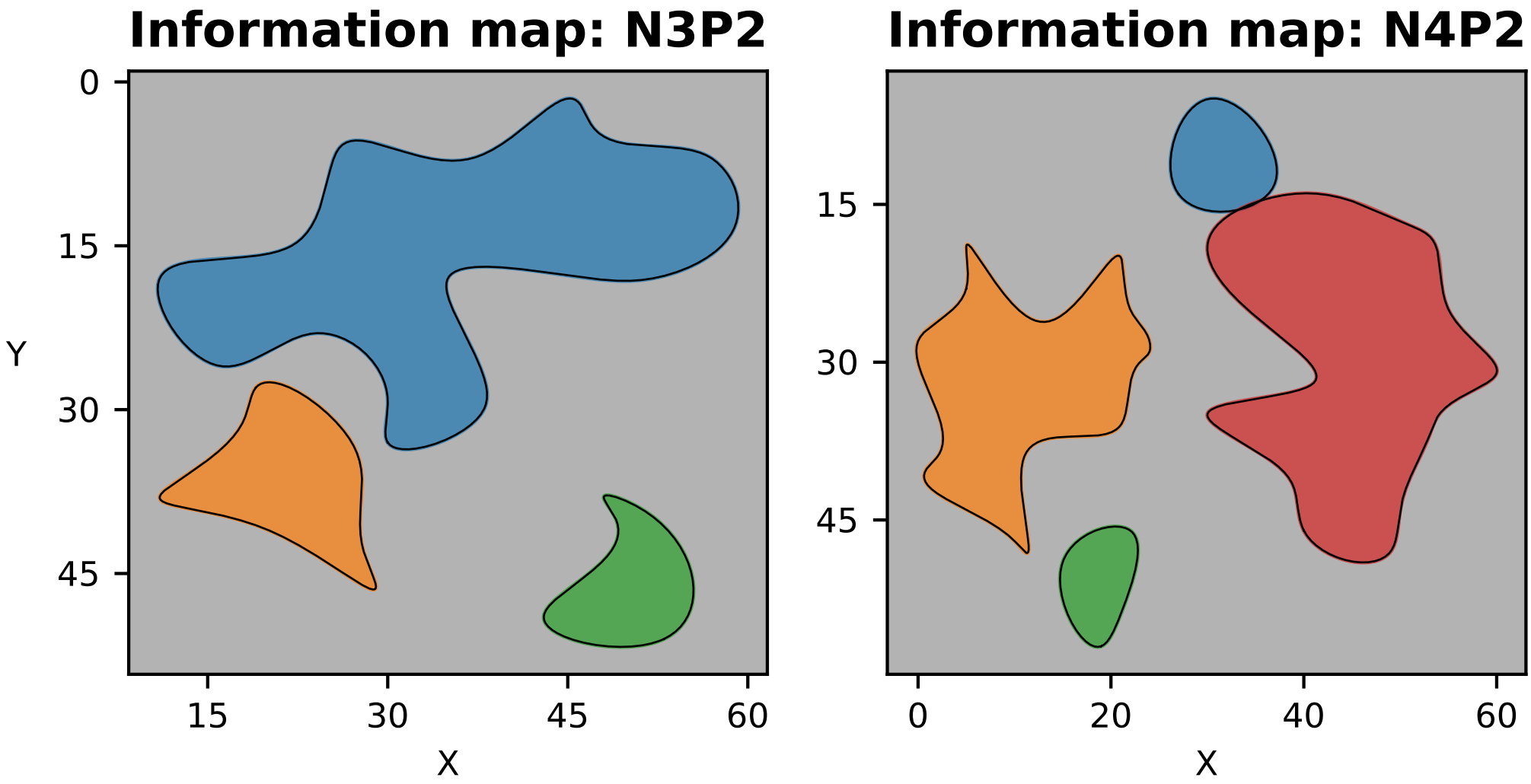

Information map: N3P2
Information map: N4P2
0
15
30
45
Y
15
30
45
60
X
15
30
45
0
20
40
60
X
