# Supplementary material for: Hierarchical feature binding in a spiking neural network model of the primate ventral visual pathway
Source: PLoS Comput Biol. 2026 Sep 16;22(9):e1014752. doi: 10.1371/journal.pcbi.1014752 (PMC13581216; doi:10.1371/journal.pcbi.1014752)
Supplement: Supplementary file 3 [file pcbi.1014752.s003.pdf]

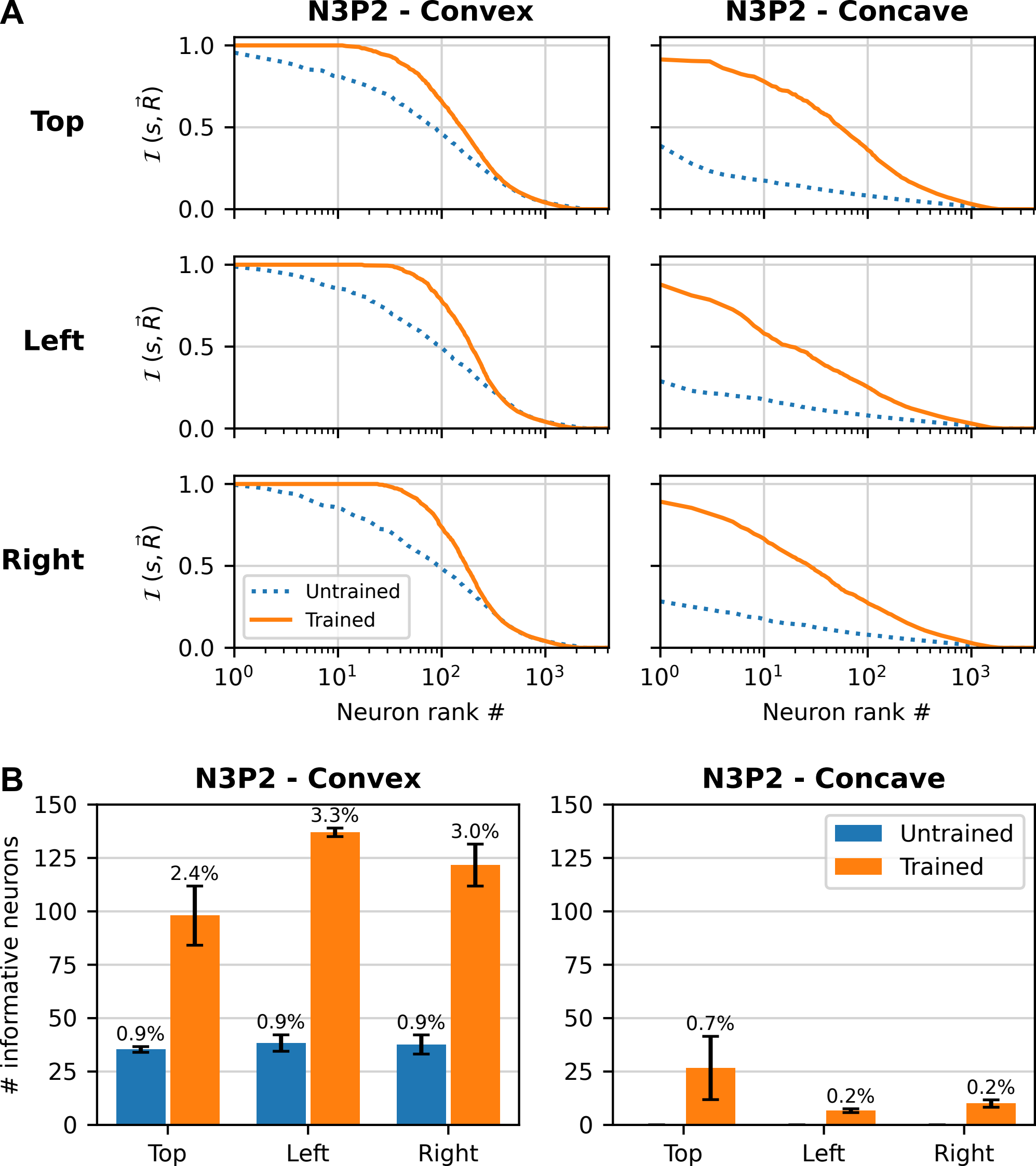
A
N3P2 - Convex
N3P2 - Concave
Top
Left
Right
$\mathcal{I}(s, \vec{R})$
1.0
0.5
0.0
10^0
10^1
10^2
10^3
Neuron rank #
Untrained
Trained
B
N3P2 - Convex
N3P2 - Concave
informative neurons
150
125
100
75
50
25
0
0.9%
2.4%
0.9%
3.3%
0.9%
3.0%
0.7%
0.2%
0.2%
Top
Left
Right
Untrained
Trained
