# Supplementary material for: Hierarchical feature binding in a spiking neural network model of the primate ventral visual pathway
Source: PLoS Comput Biol. 2026 Sep 16;22(9):e1014752. doi: 10.1371/journal.pcbi.1014752 (PMC13581216; doi:10.1371/journal.pcbi.1014752)
Supplement: Supplementary file 4 [file pcbi.1014752.s004.pdf]

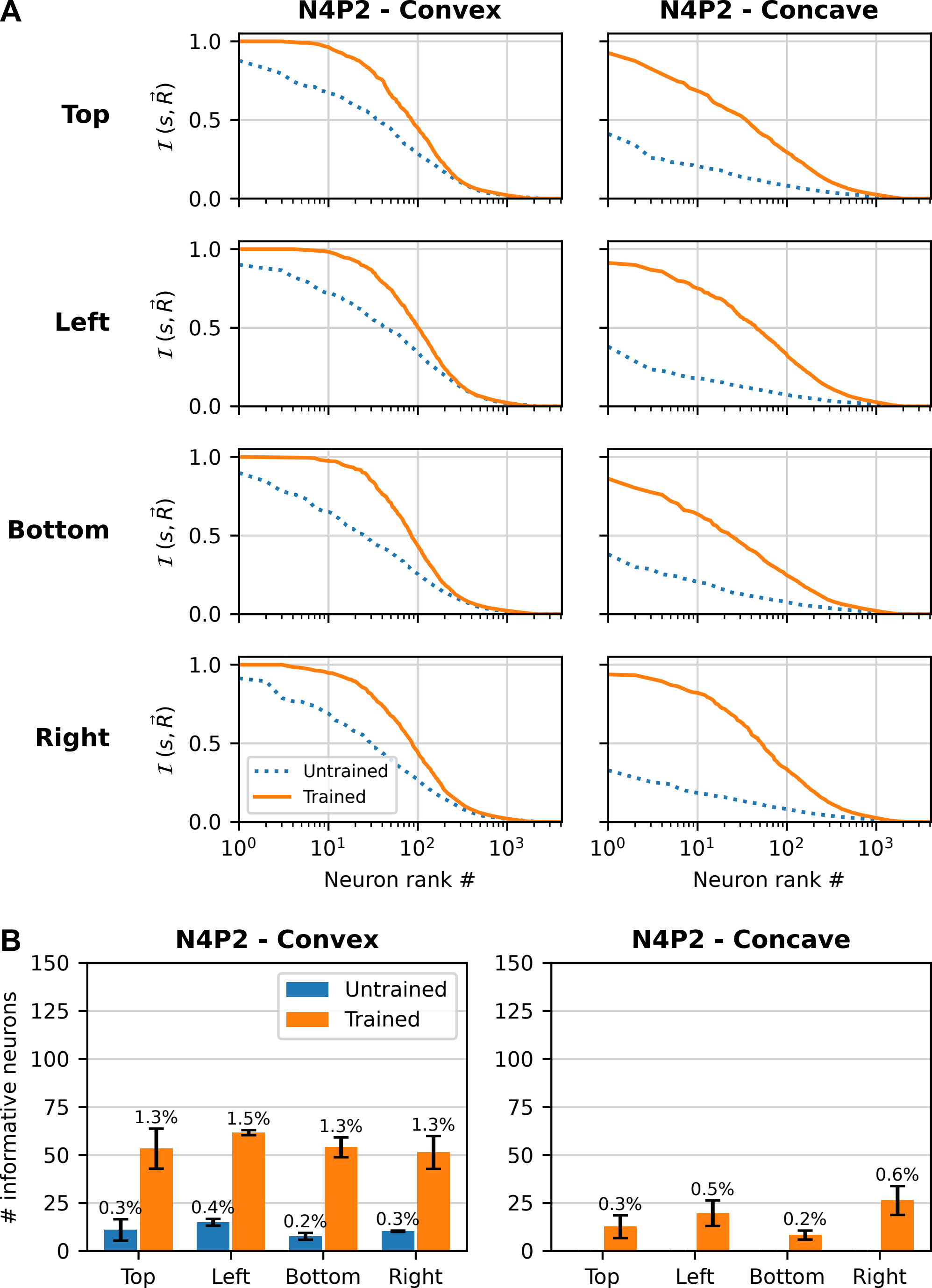
A
N4P2 - Convex
N4P2 - Concave
Top
Left
Bottom
Right
$\mathcal{I}(s, \vec{R})$
1.0
0.5
0.0
10^0
10^1
10^2
10^3
Neuron rank #
Untrained
Trained
B
N4P2 - Convex
N4P2 - Concave
informative neurons
150
125
100
75
50
25
0
Untrained
Trained
0.3%
1.3%
0.4%
1.5%
0.2%
1.3%
0.3%
1.3%
0.3%
0.5%
0.2%
0.6%
Top
Left
Bottom
Right
