# Supplementary material for: Hierarchical feature binding in a spiking neural network model of the primate ventral visual pathway
Source: PLoS Comput Biol. 2026 Sep 16;22(9):e1014752. doi: 10.1371/journal.pcbi.1014752 (PMC13581216; doi:10.1371/journal.pcbi.1014752)
Supplement: Supplementary file 5 [file pcbi.1014752.s005.pdf]

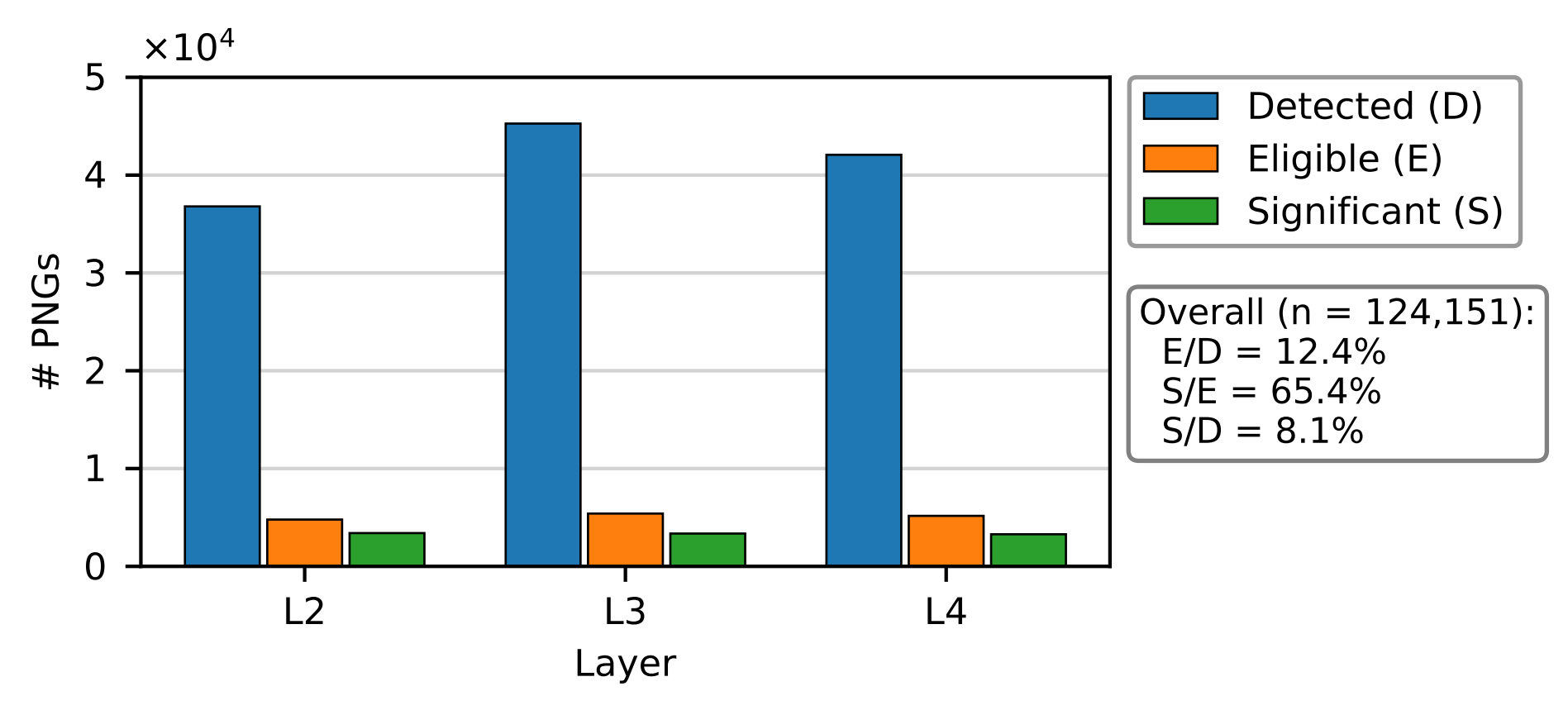

×10^4
5
4
3
2
1
0
PNGs
L2
L3
L4
Layer
Detected (D)
Eligible (E)
Significant (S)
Overall (n = 124,151):
E/D = 12.4%
S/E = 65.4%
S/D = 8.1%
