# Supplementary material for: Hierarchical feature binding in a spiking neural network model of the primate ventral visual pathway
Source: PLoS Comput Biol. 2026 Sep 16;22(9):e1014752. doi: 10.1371/journal.pcbi.1014752 (PMC13581216; doi:10.1371/journal.pcbi.1014752)
Supplement: Supplementary file 6 [file pcbi.1014752.s006.pdf]

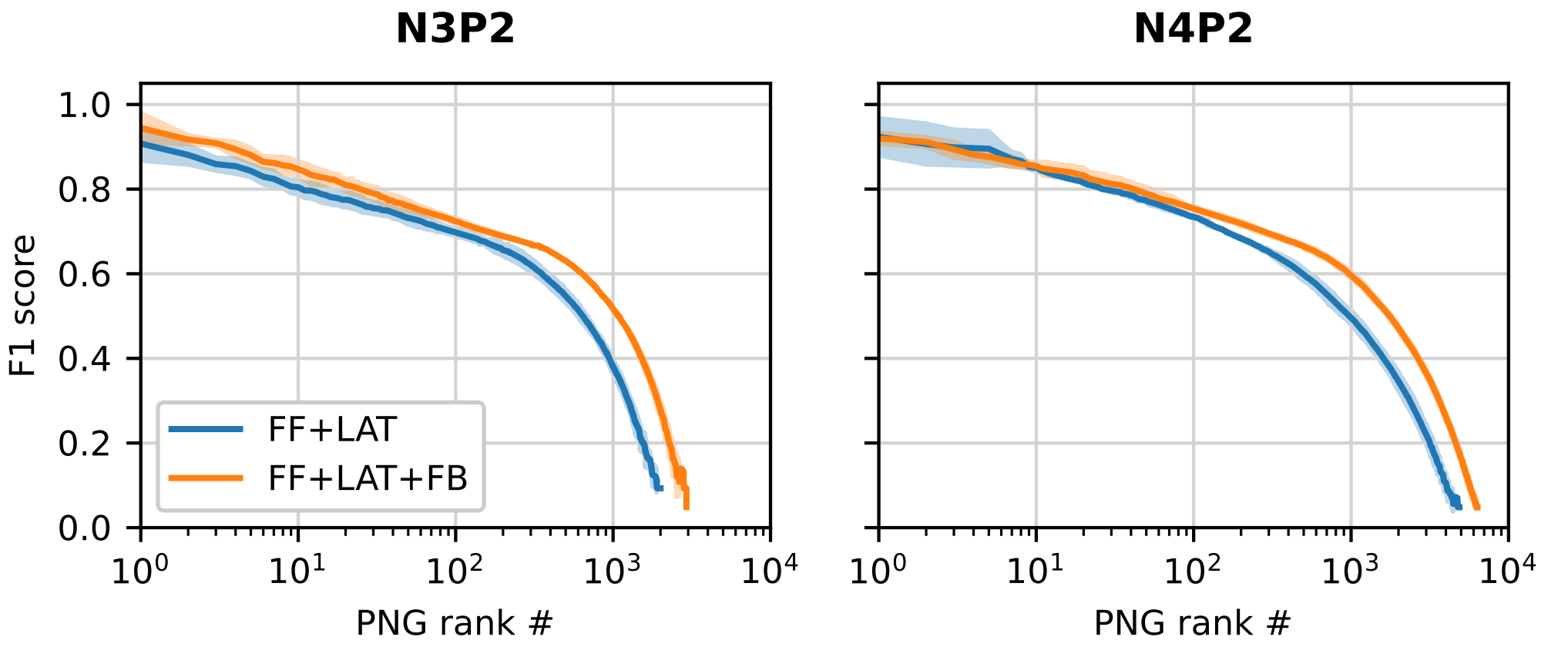
N3P2
N4P2
F1 score
1.0
0.8
0.6
0.4
0.2
0.0
10^0
10^1
10^2
10^3
10^4
PNG rank #
FF+LAT
FF+LAT+FB
