# Supplementary material for: Hierarchical feature binding in a spiking neural network model of the primate ventral visual pathway
Source: PLoS Comput Biol. 2026 Sep 16;22(9):e1014752. doi: 10.1371/journal.pcbi.1014752 (PMC13581216; doi:10.1371/journal.pcbi.1014752)
Supplement: Supplementary file 7 [file pcbi.1014752.s007.pdf]

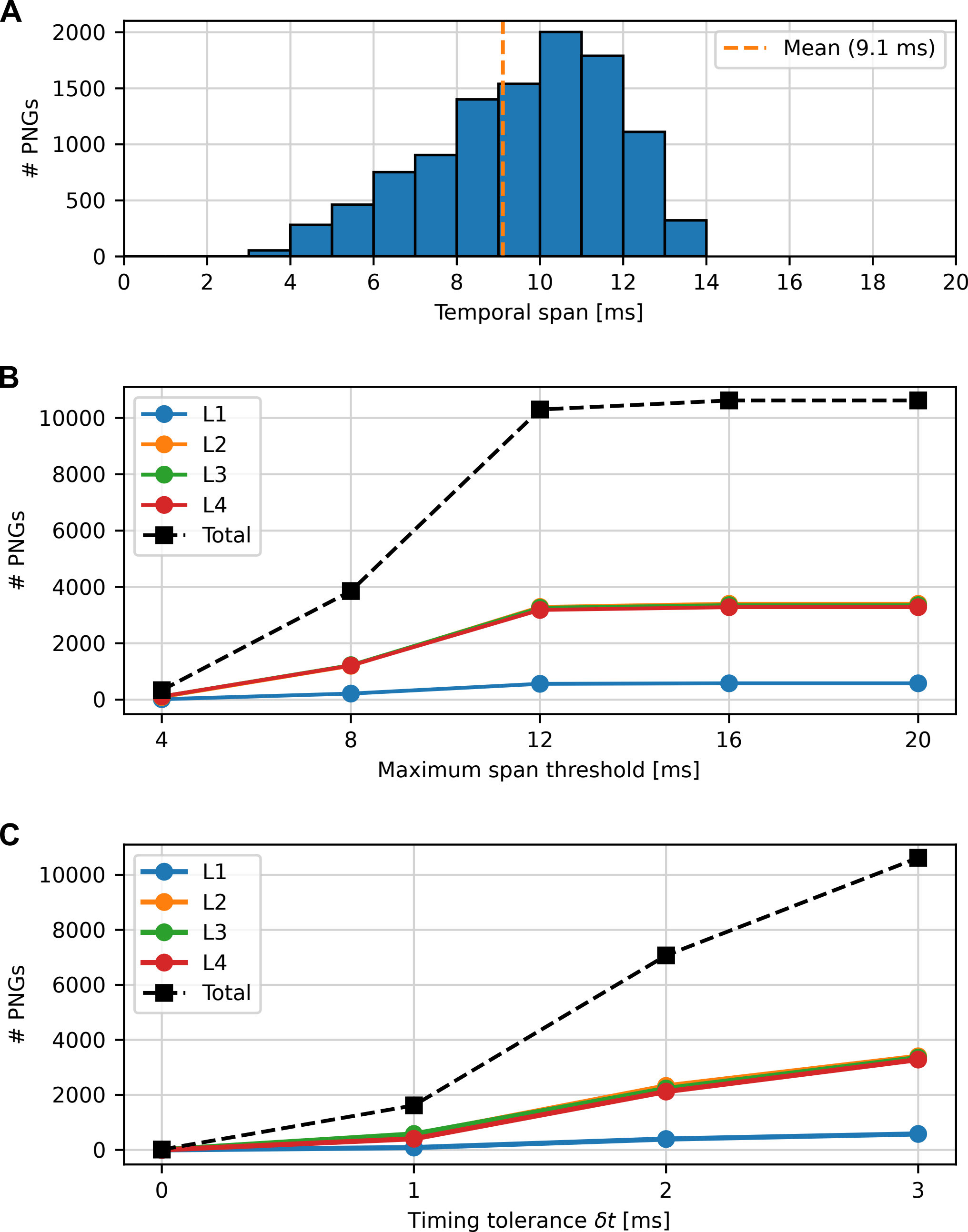
A
PNGs
Mean (9.1 ms)
Temporal span [ms]
B
L1
L2
L3
L4
Total
Maximum span threshold [ms]
C
Timing tolerance δt [ms]
