# Supplementary material for: Hierarchical feature binding in a spiking neural network model of the primate ventral visual pathway
Source: PLoS Comput Biol. 2026 Sep 16;22(9):e1014752. doi: 10.1371/journal.pcbi.1014752 (PMC13581216; doi:10.1371/journal.pcbi.1014752)
Supplement: Supplementary file 8 [file pcbi.1014752.s008.pdf]

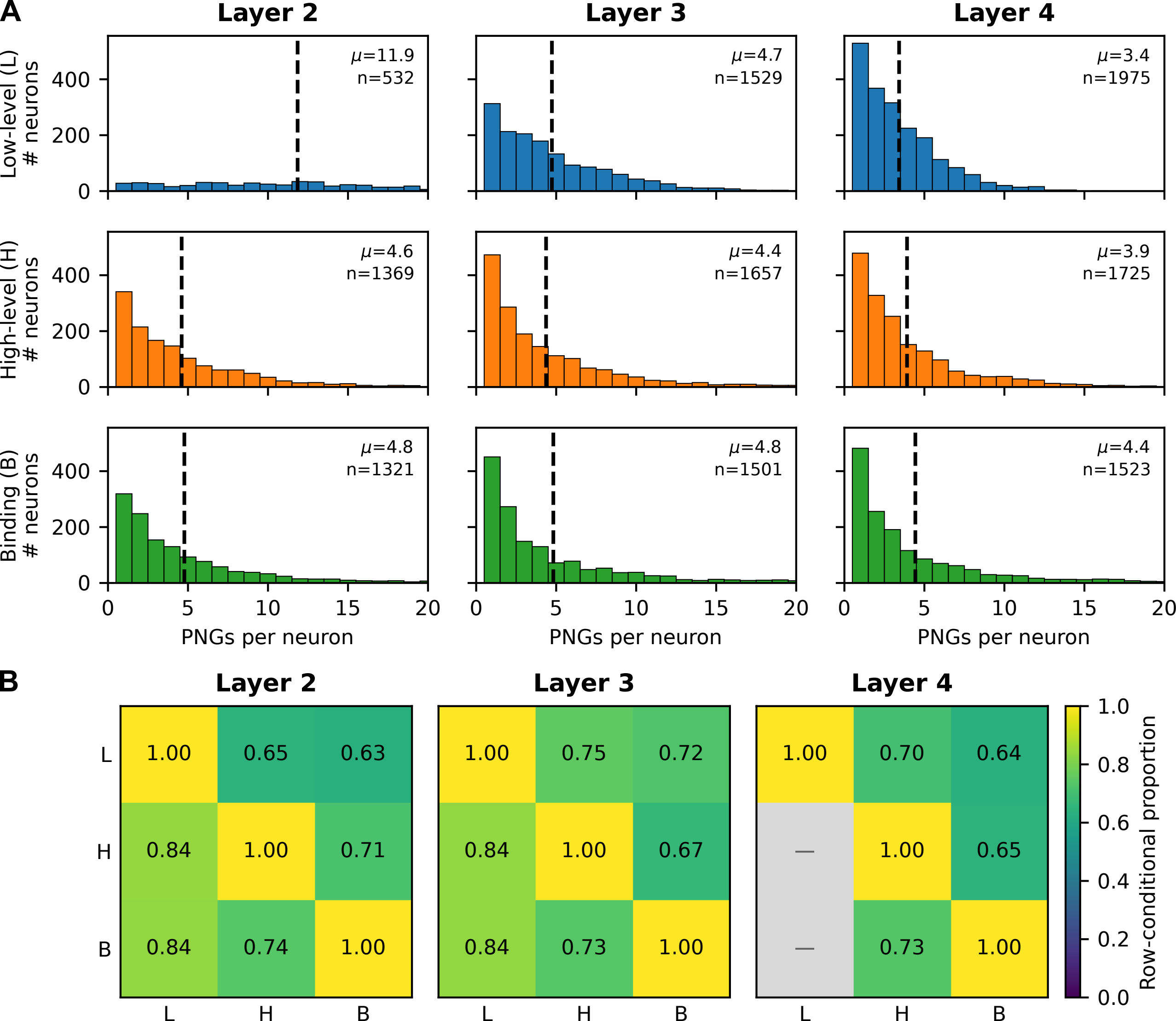
A
Layer 2
Layer 3
Layer 4
Low-level (L)
neurons
High-level (H)
neurons
Binding (B)
neurons
μ=11.9
n=532
μ=4.7
n=1529
μ=3.4
n=1975
μ=4.6
n=1369
μ=4.4
n=1657
μ=3.9
n=1725
μ=4.8
n=1321
μ=4.8
n=1501
μ=4.4
n=1523
PNGs per neuron
B
Layer 2
Layer 3
Layer 4
L
H
B
1.00
0.65
0.63
0.84
1.00
0.71
0.84
0.74
1.00
0.75
0.72
0.67
0.73
0.70
0.64
0.65
—
Row-conditional proportion
