# Supplementary material for: Hierarchical feature binding in a spiking neural network model of the primate ventral visual pathway
Source: PLoS Comput Biol. 2026 Sep 16;22(9):e1014752. doi: 10.1371/journal.pcbi.1014752 (PMC13581216; doi:10.1371/journal.pcbi.1014752)
Supplement: Supplementary file 9 [file pcbi.1014752.s009.pdf]

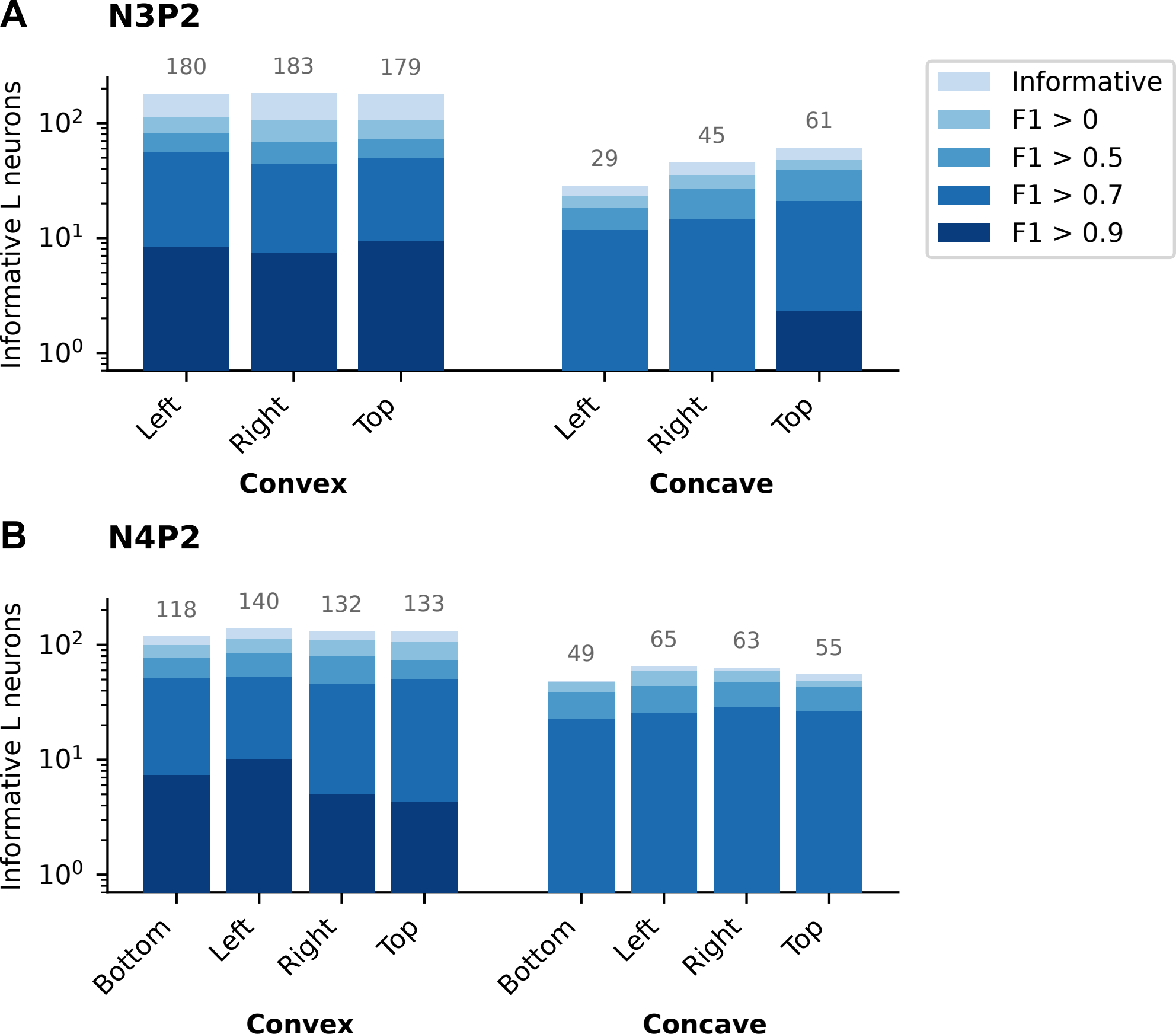
A
N3P2
180
183
179
29
45
61
Informative
F1 > 0
F1 > 0.5
F1 > 0.7
F1 > 0.9
Informative L neurons
10^2
10^1
10^0
Left
Right
Top
Left
Right
Top
Convex
Concave
B
N4P2
118
140
132
133
49
65
63
55
Informative L neurons
10^2
10^1
10^0
Bottom
Left
Right
Top
Bottom
Left
Right
Top
Convex
Concave
