# Supplementary material for: Hierarchical feature binding in a spiking neural network model of the primate ventral visual pathway
Source: PLoS Comput Biol. 2026 Sep 16;22(9):e1014752. doi: 10.1371/journal.pcbi.1014752 (PMC13581216; doi:10.1371/journal.pcbi.1014752)
Supplement: Supplementary file 10 [file pcbi.1014752.s010.pdf]

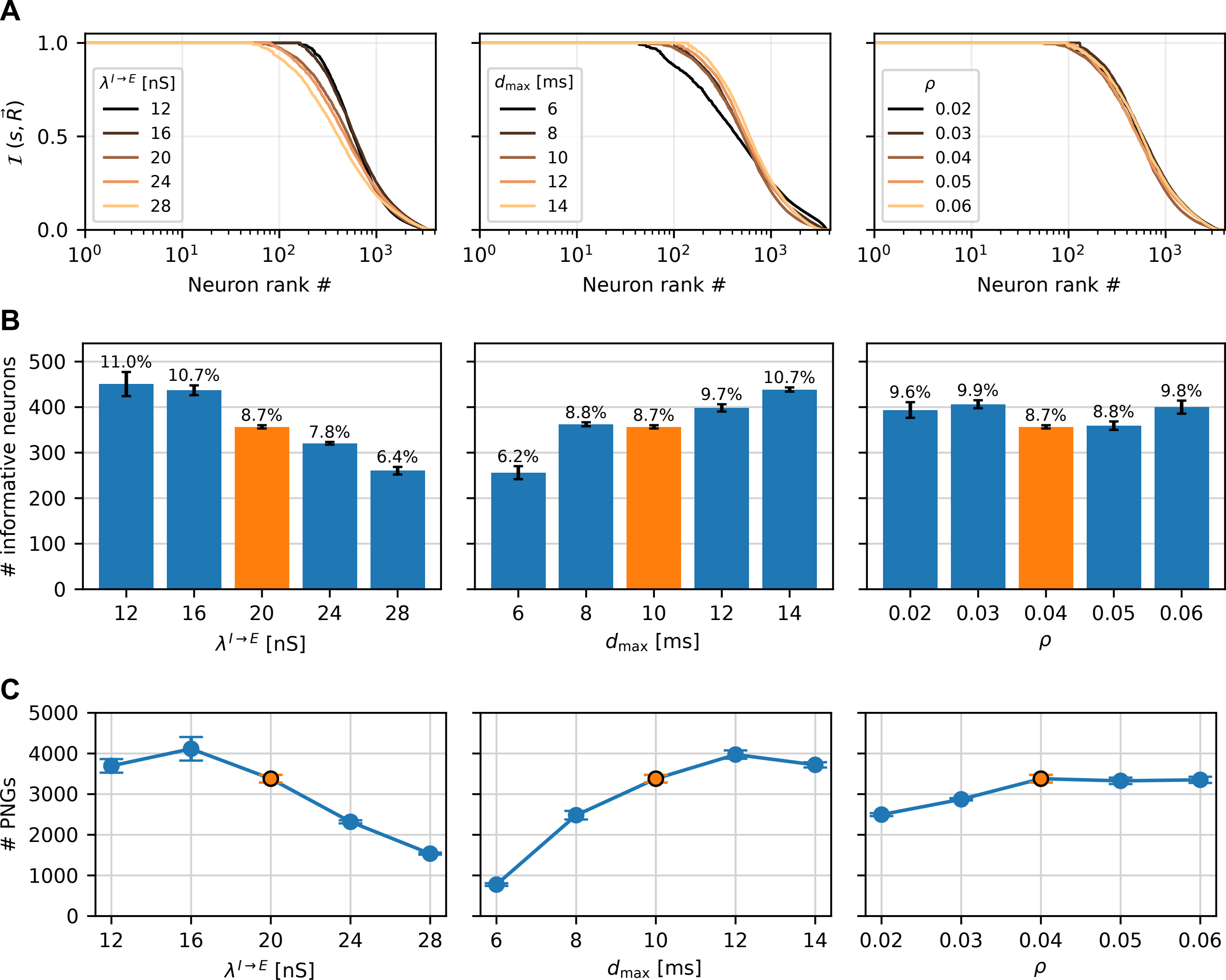
A
$\mathcal{I}(s, \vec{R})$
$\lambda^{I \to E}$ [nS]
12
16
20
24
28
d_{max} [ms]
6
8
10
12
14
ρ
0.02
0.03
0.04
0.05
0.06
Neuron rank #
B
informative neurons
11.0%
10.7%
8.7%
7.8%
6.4%
6.2%
8.8%
8.7%
9.7%
10.7%
9.6%
9.9%
8.7%
8.8%
9.8%
$\lambda^{I \to E}$ [nS]
d_{max} [ms]
ρ
C
PNGs
$\lambda^{I \to E}$ [nS]
d_{max} [ms]
ρ
