# Supplementary material for: Hierarchical feature binding in a spiking neural network model of the primate ventral visual pathway
Source: PLoS Comput Biol. 2026 Sep 16;22(9):e1014752. doi: 10.1371/journal.pcbi.1014752 (PMC13581216; doi:10.1371/journal.pcbi.1014752)
Supplement: Supplementary file 11 [file pcbi.1014752.s011.pdf]

Dear Editor,

In their manuscript, *“Hierarchical feature binding in a spiking neural network model of the primate ventral visual pathway”*, the authors build on the previous works that suggest polysynchrony as a biologically plausible binding solution in vision, and advance the methodology for detailed empirical validation of this conceptual framework. Concretely, the significant contribution of this work is: i) introduction of a novel dataset with convex vs. concave boundary stimuli, ii) explicit and systematic detection of binding motifs (SPADE analysis), iii) quantitative evaluation of selectivity of detected motifs (F1-scores), and iv) explicit tests of robustness to jitter/noise.

The manuscript is clearly written, and the methodological description is sufficiently detailed to allow reproducibility. The introduction of ablation studies, along with careful reporting of implementation details, further supports the technical rigor of the work. Overall, the study presents a thoughtful and biologically grounded extension of earlier polychronization-based binding models, and the results are presented systematically and transparently.
While the study is carefully executed, several aspects could be strengthened to improve both the clarity of the claims and the impact of the results. Here is a list of my suggestions for each section.

1. Introduction

1.1. The introduction is somewhat lengthy and would benefit from streamlining, with a clearer focus on motivating the study, highlighting the novel contributions, and briefly pointing to the main findings. Background material could be moved into a separate *related work* section to better position this contribution in the literature.

1.2. Lines 17–20: *“Given the core role…”* --> *“…feature binding has long been thought by psychologists and neuroscientists to underpin consciousness, itself”* requires justification or citation.

- 1.3. Line 74: *“The hierarchical character of visual perception mirrors the hierarchical architecture of the visual brain, which is comprised of a hierarchy of ascending areas.”* While this framing is intuitively appealing, it remains ambiguous given the multiple definitions of “hierarchy” in neuroscience (Hilgetag & Goulas, 2020; DOI: 10.1098/rstb.2019.0319). In practice, the hierarchy in the present model is best understood as a functional hierarchy: representations become progressively more abstract across layers, with binding operations occurring at higher levels (e.g., edges --> contours --> objects), rather than a structural hierarchy reflecting anatomical specialization. In Hilgetag & Goulas’ taxonomy, this aligns most closely with a *progression of scales* (nested feature representations), and less with laminar projection sorting, cytoarchitectonic gradients, or strict topological ordering. Clarifying this would avoid overinterpretation and position the work more accurately.

2. Materials and Methods

2.1. Table 1 shows that network parameters are homogeneous across layers. It would be useful to clarify how this homogeneity influences the emergence of PSGs and binding neurons. Are the hierarchical motifs simply a consequence of depth and STDP dynamics, or would introducing biologically inspired heterogeneity (e.g., varying neuron numbers, connection densities, receptive field sizes) change the nature of binding? Some speculation would strengthen the link to cortical diversity.
2.2. Although the work is positioned as an advance, the core conceptual framework (polychronization via STDP with conduction delays) is largely inherited from Eguchi (2018) and Isbister (2018). The authors do mention rate-coded assemblies and VisNet-style conjunction units in their discussion, but they do not provide direct benchmarks or performance comparisons. Including such evaluations would demonstrate more convincingly the extent to which polychronous binding provides explanatory power beyond these existing models.

2.3. While the ablations with jitter/noise are useful, the paper would benefit from exploration of scalability in two senses:

- Stimuli: more complex inputs (occluded contours or multi-object scenes).
- Model: increased number of layers or higher neuron densities to test whether polychronous binding motifs persist as the architecture scales.
  Even a discussion or preliminary simulations would strengthen the generality of the claims.

2.4. The detection of PNGs is restricted to short temporal spans and specific 3-neuron motifs. A sensitivity analysis varying the detection window and motif size would reassure readers that results are robust to analysis parameters.

2.5. The manuscript notes that implementation details are available, but the provided code is hosted on the University of Surrey GitLab, which is restricted to account holders and not publicly accessible. This prevents reviewers (and eventually readers) from verifying reproducibility.

3. Results

3.1. Analyses emphasize convex-selective neurons, while concave boundaries and multiple concavity combinations are underreported, though present in the dataset. Including parallel analyses for concave features would provide a more balanced view and test whether the model develops true category selectivity rather than convexity bias.
3.2. Example neurons (Figs. 6–7) appear more retinotopic than feature-category selective. A useful addition would be to quantify spatial invariance versus category selectivity, e.g. by translating convex/concave boundaries across input positions and measuring tuning curve stability (half-width at half-maximum). This would clarify whether binding mechanisms are feature-invariant or location-dependent.

3.3. Line 1081 links early PNG activation to selective responses. Could the authors clarify which mechanisms produce heterogeneous onset times? Is variability driven mainly by random conduction delays and STDP-shaped pathways, or do lateral competition and feedback play a role? Ablation experiments (e.g., fixed delays, no competition) would clarify whether timing diversity is emergent or design-dependent.

3.4. The absence of narrowband oscillations in population spectra is interpreted as evidence that the network is "optimized for polychronous activity." This inference is too strong: lack of oscillations should be described more cautiously as "not rhythm-mediated."

4. Discussion

4.1. The authors mention adversarial attacks in CNNs as motivation, but do not clarify whether their SNN model is expected to be more or less susceptible. Given that polychronous motifs rely on precise spike timing and appear robust to jitter/noise, one could hypothesize reduced vulnerability compared to rate-based CNNs. At the same time, strong retinotopy and homogeneous layers might leave the model open to targeted contour perturbations. A short discussion of expectations, or how adversarial robustness could be tested, would strengthen the broader relevance of the work.

4.2. Feedback is described as promoting attractor states that "minimize energy." Unless a formal energy function or convergence metric is provided, this language should be moderated.

4.3. Links to holographic principles or prefrontal working memory are interesting but speculative. These could be moved to a "future directions" section with clearer caveats.

In summary, the manuscript presents a technically careful and biologically motivated extension of earlier polychronization-based binding models, with useful innovations in dataset design and motif detection. However, several substantive issues remain regarding the balance of convex vs. concave analyses, the extent of spatial invariance, the robustness of results to analysis choices, and the strength of some claims relative to prior work. Addressing these points would significantly improve the clarity and impact of the paper.

Kind regards,
