# Supplementary material for: Hierarchical feature binding in a spiking neural network model of the primate ventral visual pathway
Source: PLoS Comput Biol. 2026 Sep 16;22(9):e1014752. doi: 10.1371/journal.pcbi.1014752 (PMC13581216; doi:10.1371/journal.pcbi.1014752)
Supplement: Supplementary file 12 [file pcbi.1014752.s013.pdf]

Response to Reviewers

Dear Editor and Reviewers,

We thank the Editor and Reviewers for their constructive feedback. We have revised the manuscript accordingly and provide below a point-by-point response. Where possible, we indicate where changes were made in the revised manuscript using figure numbers and line numbers (based on the clean revised manuscript). All changes are also visible in the tracked-changes version of the manuscript.

Kind regards,

Brian Gardner on behalf of all the authors

Overview of key changes in the revised manuscript:

- Substantially revised the Introduction to sharpen the motivation, scope, and positioning of the work, and to reduce tangential material.
- Re-ran the single-neuron stimulus-specific information analyses and updated the corresponding text/figures to more correctly quantify (i) the *maximum information* conveyed by each final-layer neuron about convex vs. concave boundary elements at its *preferred side*, and (ii) the *side-resolved* information conveyed by each final-layer neuron for convex vs. concave boundary elements *across all object sides*.
- Re-ran PNG detections (SPADE) to incorporate the *upper* tolerance on delay-matched spike-timing differences (now explicitly accounted for) and updated the reported PNG/HFB analyses to reflect these detections.
- Added a new occlusion experiment to probe the dependence of neural selectivity on a progressively masked left-convex boundary element (reported in Fig. 13).
- Made the full codebase publicly available at the time of resubmission, at https://github.com/BCGardner/feature_binding, including the configuration files/scripts required to regenerate the reported experiment results. Documented notebooks reproduce all of the manuscript figures.
- Several of the main text figures have been combined to assist narrative flow and facilitate direct comparison of results.
- In response to the reviewer comments, nine new supplementary figures have been added that extend our analyses.

Reviewer 1

In their manuscript, *"Hierarchical feature binding in a spiking neural network model of the primate ventral visual pathway"*, the authors build on the previous works that suggest polysynchrony as a biologically plausible binding solution in vision, and advance the methodology for detailed empirical validation of this conceptual framework. Concretely, the significant contribution of this work is: i) introduction of a novel dataset with convex vs. concave boundary stimuli, ii) explicit and systematic detection of binding motifs (SPADE analysis), iii) quantitative evaluation of selectivity of detected motifs (F1-scores), and iv) explicit tests of robustness to jitter/noise.

The manuscript is clearly written, and the methodological description is sufficiently detailed to allow reproducibility. The introduction of ablation studies, along with careful reporting of implementation details, further supports the technical rigor of the work. Overall, the study presents a thoughtful and biologically grounded extension of earlier polychronization-based binding models, and the results are presented systematically and transparently.

While the study is carefully executed, several aspects could be strengthened to improve both the clarity of the claims and the impact of the results. Here is a list of my suggestions for each section.

1. Introduction

1.1. The introduction is somewhat lengthy and would benefit from streamlining, with a clearer focus on motivating the study, highlighting the novel contributions, and briefly pointing to the main findings. Background material could be moved into a separate related work section to better position this contribution in the literature.

We thank the reviewer for this feedback. We have substantially revised and streamlined the Introduction to improve focus and logical organisation. In doing so, we cut tangential background material, removed duplicated exposition, and restructured the narrative around (i) the binding problem and the specific gap addressed, (ii) the biological motivation and the meaning of "hierarchy" as used in this paper, and (iii) a concise description of the modelling context that directly leads to the binding-by-polychrony mechanism. We also added an explicit statement of our main advance beyond Eguchi and Isbister et al. 2018, namely moving from a proof-of-principle demonstration to a systematic quantitative test under a larger and more structured boundary-shape ensemble, with an explicit focus on three-neuron HFB circuits and their emergence across all layers. To better position the work, we consolidated prior modelling background into a dedicated "Related modelling work" subsection, and retained only the material required to motivate the study questions.

1.2. Lines 17–20: "Given the core role..." --> "...feature binding has long been thought by psychologists and neuroscientists to underpin consciousness, itself" requires justification or citation.

We removed this strong claim and replaced it with a more defensible motivation that is directly aligned with the present modelling question. The revised text (lines 15-21) now frames the key challenge as specifying how binding can be implemented in hierarchical neural circuits in a way that scales across spatial levels and remains compatible with realistic axonal delays and spiking dynamics, with appropriate citations to relevant binding frameworks (Treisman, 1998; Singer, 2001). We also briefly acknowledge complementary single-neuron binding accounts via

dendritic nonlinearities (Tran-Van-Minh et al., 2015; Tang et al., 2025), while clarifying that the focus of this paper is population-level and between-layer encoding of "part-of" relations.

1.3. Line 74: "The hierarchical character of visual perception mirrors the hierarchical architecture of the visual brain, which is comprised of a hierarchy of ascending areas." While this framing is intuitively appealing, it remains ambiguous given the multiple definitions of "hierarchy" in neuroscience (Hilgetag & Goulas, 2020; DOI: 10.1098/rstb.2019.0319). In practice, the hierarchy in the present model is best understood as a functional hierarchy: representations become progressively more abstract across layers, with binding operations occurring at higher levels (e.g., edges --> contours --> objects), rather than a structural hierarchy reflecting anatomical specialization. In Hilgetag & Goulas' taxonomy, this aligns most closely with a progression of scales (nested feature representations), and less with laminar projection sorting, cytoarchitectonic gradients, or strict topological ordering. Clarifying this would avoid overinterpretation and position the work more accurately.

We agree and have revised the Introduction to explicitly disambiguate our use of "hierarchy." We now include a short paragraph (lines 54-62) that (i) notes that "hierarchy" is used in several distinct ways in systems neuroscience, (ii) states that in this paper we use it primarily in a functional sense (progressively more abstract representations across stages, with smaller-scale features forming constituent parts for larger-scale features), and (iii) aligns this usage with the "progression of scales" taxonomy described by Hilgetag & Goulas (2020). We also adjusted the opening of the ventral-pathway subsection (lines 82-90) to avoid implying an anatomical or laminar-projection definition, and instead introduce the ventral pathway as a set of interconnected areas commonly described as supporting increasing levels of representational abstraction.

2. Materials and Methods

2.1. Table 1 shows that network parameters are homogeneous across layers. It would be useful to clarify how this homogeneity influences the emergence of PSGs and binding neurons. Are the hierarchical motifs simply a consequence of depth and STDP dynamics, or would introducing biologically inspired heterogeneity (e.g., varying neuron numbers, connection densities, receptive field sizes) change the nature of binding? Some speculation would strengthen the link to cortical diversity.

We agree that clarifying the role of parameter homogeneity versus cortical heterogeneity would strengthen interpretation. We have added a Discussion paragraph in Future Work (lines 1574-1590) addressing this point. We clarify that although neuron and synapse parameters are largely homogeneous across layers for computational tractability (Table 1), hierarchical organisation is still introduced by the layer-dependent increase in fan-in (receptive field size), and the emergence of PNGs and binding neurons follows from local STDP acting on heterogeneous axonal delays together with competitive E/I dynamics. We further speculate that introducing biologically inspired heterogeneity (e.g., layer-specific neuron numbers, connection densities, or time constants) would be expected to modulate where and at what spatial scales PNGs/binding motifs emerge, rather than eliminate the underlying delay-matched binding mechanism, and may enrich the diversity of polychronous circuits.

2.2. Although the work is positioned as an advance, the core conceptual framework (polychronization via STDP with conduction delays) is largely inherited from Eguchi (2018) and Isbister (2018). The authors do mention rate-coded assemblies and VisNet-style conjunction

units in their discussion, but they do not provide direct benchmarks or performance comparisons. Including such evaluations would demonstrate more convincingly the extent to which polychronous binding provides explanatory power beyond these existing models.

We recognise that the underlying mechanism for polychronization (STDP acting on heterogeneous conduction delays) follows the framework introduced by Eguchi and Isbister et al. 2018. Our advance is in how that mechanism is tested and evaluated: we train hierarchical SNNs on ecologically realistic shape sets in which many objects are built from a shared set of boundary contour elements (convex/concave per side), so that competitive learning is pressured to form neurons selective for those feature primitives and their hierarchy. We then go beyond existence claims by providing a quantitative assessment of both single-neuron feature selectivity and polychronous binding structure: we use SPADE with explicit post-processing constraints to systematically detect three-neuron HFB circuits, and we evaluate what these motifs represent (including precision/recall/F1) and how their prevalence depends on training and architecture. This scope is also stated explicitly in the Introduction (lines 33-40) and Discussion as a key way the present work extends prior polychronization studies.

On direct benchmarks against VisNet-style rate-coded models (and other ANN baselines), we agree these would be informative, but we consider them out of scope for this paper given our current focus. As noted in the Discussion/Future Work and the newly added “Limitations and scope” (lines 1413-1423), we did not attempt translation/invariance learning here and present stimuli at a fixed retinal position. We view incorporating transformations and invariance learning as the appropriate next step for meaningful head-to-head benchmarking against VisNet and modern ANNs.

2.3. While the ablations with jitter/noise are useful, the paper would benefit from exploration of scalability in two senses:

- *Stimuli: more complex inputs (occluded contours or multi-object scenes).*
- *Model: increased number of layers or higher neuron densities to test whether polychronous binding motifs persist as the architecture scales. Even a discussion or preliminary simulations would strengthen the generality of the claims.*

We understand that exploring scalability is important, both in terms of stimulus complexity and architectural scale. To address the reviewer's first point (stimuli), we added a new occlusion experiment that probes the extent to which learned feature-selective representations persist under gradual loss of object contour information. Specifically, we applied a "sliding curtain" mask to the Gabor filter outputs corresponding to the left-side convex boundary element and progressively increased occlusion from 0% to 100% during inference. We then recomputed stimulus-specific information (SSI) for all final-layer excitatory neurons.

The new results (Fig. 13, “Feature selectivity is resilient to partial visual occlusion”, lines 902-924) show a graded reduction in SSI and in the number of informative neurons (SSI > 2/3 bits), with a subset of neurons remaining selective up to intermediate occlusion levels (25–50%) and selectivity collapsing only once the affected contour region is largely removed (75–100%). This supports the interpretation that the learned feature representations are robust to small amounts of input degradation.

For architectural scaling (depth/density), we did not add new simulations in this revision, but we expanded the Discussion (“Future work”, lines 1575-1591) to outline how the same local mechanisms (heterogeneous delays, STDP, and competitive dynamics) are expected to persist

under increased depth/density, while noting that systematic scaling sweeps are constrained by the rapidly increasing computational cost of PNG detection. We identify this as an important direction for future work, together with more efficient spike-pattern discovery methods.

2.4. The detection of PNGs is restricted to short temporal spans and specific 3-neuron motifs. A sensitivity analysis varying the detection window and motif size would reassure readers that results are robust to analysis parameters.

As part of our response, we have refined our PNG detection algorithm to strictly account for the upper timing tolerance (3 ms) of axonal conduction delays between low-level and binding neurons (which was previously missing). This ensures that all reported PNGs (see revised Fig. 14) are strictly compliant with the temporal constraints of the model. These re-run PNG detections, including their significance testing, have not changed the qualitative results.

In response to the temporal spans, we now report an explicit sensitivity analysis of the PNG post-processing criteria in Supplementary Fig. S7 (see the new subsection "Sensitivity of PNG detection to temporal span and timing tolerance", lines 975-983). Specifically, we (i) quantify the distribution of detected triplet temporal spans, and (ii) show how the final PNG count varies as a function of the maximum span threshold and the delay-matching tolerance (delta t). These results demonstrate that the qualitative conclusions are not driven by a single, finely tuned choice of detection parameters. Together with the corrected enforcement of the 3 ms delay-matching tolerance, this strengthens the methodological basis for the revised PNG analyses.

Finally, we have addressed the request to vary motif size by adding Supplementary Fig. S8 (lines 1059-1065), which quantifies how high-level feature neurons are reused across multiple detected triplet PNGs, indicating that binding relationships are many-to-many rather than one-to-one. A systematic extension to higher-order (larger) motifs is beyond the scope of the present work, as SPADE-based detection and statistical validation become computationally prohibitive for more complex circuits (this limitation has been discussed on lines 1297-1306). Accordingly, we frame this study as a proof-of-concept demonstration of HFB using the simplest canonical three-neuron motif.

2.5. The manuscript notes that implementation details are available, but the provided code is hosted on the University of Surrey GitLab, which is restricted to account holders and not publicly accessible. This prevents reviewers (and eventually readers) from verifying reproducibility.

The code has since been made publicly available on my personal GitHub page at https://github.com/BCGardner/feature_binding, along with documentation and notebooks for plotting the figures within the manuscript. The workflows necessary to re-generate the experiment artifacts (e.g. spike recordings, PNG detections, etc.) are also included, and example use cases described.

3. Results

3.1. Analyses emphasize convex-selective neurons, while concave boundaries and multiple concavity combinations are underreported, though present in the dataset. Including parallel analyses for concave features would provide a more balanced view and test whether the model develops true category selectivity rather than convexity bias.

We agree that reporting only convex-selective results can give an incomplete picture. In the revised manuscript we now expand the Methods text ("Single cell information analysis", lines

575-583) describing our analysis in more detail; for the convex-focused analyses in the main text, we report convex selectivity by retaining, for each neuron, the maximum stimulus-specific information across object sides only when the neuron fires more strongly for convex than concave at its preferred side (otherwise its convex-selective information is set to zero).

We have also corrected the implementation of the rank-ordered single-neuron information analysis used in the Results subsection ("Single neuron information analysis demonstrates increased feature selectivity"). Specifically, information is now pooled per neuron by taking the maximum across sides (rather than averaging across sides), consistent with the aim of quantifying each neuron's strongest feature selectivity independent of side location. This change affects the absolute counts/proportions reported in the corresponding summary plots (and increases them relative to the previous implementation), but does not alter the qualitative conclusion that STDP training increases the prevalence and strength of selective output responses across datasets and architectures.

To address the reviewer's request directly, we additionally include a parallel analysis for concave-selective neurons (line 814; Supplementary Fig. S1), and concave-selective feature maps (line 846; Supplementary Fig. S2). We have also included a new Fig. for the PNG F1-scored performance regarding concavity (line 1008; Fig. S6). Comparison of the convex- and concave-selective analyses indicates a bias towards convex-selective responses under the same criteria. Furthermore, and in response to a related reviewer comment, we also provide *side-resolved* analyses for both convex and concave selectivity (line 830; Figs. S3, S4), enabling direct comparison across sides and demonstrating that the observed convexity bias persists under the same selection criteria. This convexity bias is consistent with neurophysiological reports of a bias towards convex contour features in macaque area V4 (Pasupathy & Connor, 1999), see revised lines 1231-1232.

3.2. Example neurons (Figs. 6–7) appear more retinotopic than feature-category selective. A useful addition would be to quantify spatial invariance versus category selectivity, e.g. by translating convex/concave boundaries across input positions and measuring tuning curve stability (half-width at half-maximum). This would clarify whether binding mechanisms are feature-invariant or location-dependent.

In the present study, all shapes are presented at a fixed, centred retinal location during both training and inference. As a result, selectivity to a particular object side (e.g., left/right/top) necessarily maps onto a consistent retinotopic region, and the traceback/sensitivity maps in Figs. 6–7 (now Figs. 5-6) are therefore expected to appear spatially localised. We now clarify this explicitly in the Results text for Fig. 5 (lines 754-761): the firing-rate comparisons in panel A quantify boundary-conformation selectivity (convex vs. concave) within this fixed retinal frame by averaging responses over all shapes sharing the specified side/conformation, irrespective of the remaining sides.

We agree that a direct quantification of translation invariance (e.g., translating boundary elements across retinal positions and measuring tuning stability) would be valuable. However, this requires training and testing with translated stimuli and an invariance-learning mechanism, and is beyond the scope of the current work. We outline this in the Discussion ("Limitations and scope", and "Future work"), noting that biologically plausible mechanisms such as continuous transformation learning and trace learning provide a route for learning translation-invariant feature and binding responses.

3.3. Line 1081 links early PNG activation to selective responses. Could the authors clarify which mechanisms produce heterogeneous onset times? Is variability driven mainly by random conduction delays and STDP-shaped pathways, or do lateral competition and feedback play a role? Ablation experiments (e.g., fixed delays, no competition) would clarify whether timing diversity is emergent or design-dependent.

The reviewer raises an interesting question as to how different neural network mechanisms each affect the PNG onset time distribution.

The PNG onset analysis that we present is specifically intended to identify the time at which the most feature-selective PNGs occur in response to a presented input. We link these most selective PNGs to the early spike in activity. Our study is not directly concerned with tuning the network architecture and parameters to vary the onset time distribution, as these elements have already been carefully tuned to produce a biologically plausible simulation that maximises the emergence of feature-selective PNGs. As well as extending the scope, these additional experiments pose some challenging practical hurdles: fixed conduction delays would ablate all PNGs; eliminating lateral inhibition leads to biologically unrealistic behaviour and explosive activity, meaning that all other parameters would need re-adjusting, which would not be comparing like-for-like.

3.4. The absence of narrowband oscillations in population spectra is interpreted as evidence that the network is "optimized for polychronous activity." This inference is too strong: lack of oscillations should be described more cautiously as "not rhythm-mediated."

This change has been made on line 1135, and a relevant citation added.

4. Discussion

4.1. The authors mention adversarial attacks in CNNs as motivation, but do not clarify whether their SNN model is expected to be more or less susceptible. Given that polychronous motifs rely on precise spike timing and appear robust to jitter/noise, one could hypothesize reduced vulnerability compared to rate-based CNNs. At the same time, strong retinotopy and homogeneous layers might leave the model open to targeted contour perturbations. A short discussion of expectations, or how adversarial robustness could be tested, would strengthen the broader relevance of the work.

We thank the reviewer for raising this point. We acknowledge that this work does not demonstrate SNN or PNG resilience to adversarial examples, and rigorous comparison with CNNs in this regard is beyond the scope as it could be a separate paper in its own right. Therefore, we have rephrased the second paragraph in sub-section "Implications for neuroscience and AI" (lines 1440 onwards), to soften the claims, cast the idea as a hypothesis to be explored in future work, and support the basis of the idea by referencing our figures demonstrating robustness to noise.

4.2. Feedback is described as promoting attractor states that "minimize energy." Unless a formal energy function or convergence metric is provided, this language should be moderated.

We acknowledge that this language is not supported by quantified analysis, so we removed the statements that mention attractor basins and minimisation of energy.

4.3. Links to holographic principles or prefrontal working memory are interesting but speculative. These could be moved to a "future directions" section with clearer caveats.

We thank the reviewer for their interest in the discussion of holographic principles and working memory. We note that these topics were already framed as future work, and we have now strengthened this discussion by adding two citations that address the holographic principle more directly. We have also removed the more speculative paragraph that discussed specific brain regions and working memory mechanisms beyond the scope of our simulations.

In summary, the manuscript presents a technically careful and biologically motivated extension of earlier polychronization-based binding models, with useful innovations in dataset design and motif detection. However, several substantive issues remain regarding the balance of convex vs. concave analyses, the extent of spatial invariance, the robustness of results to analysis choices, and the strength of some claims relative to prior work. Addressing these points would significantly improve the clarity and impact of the paper.

Reviewer 2

This study constructed a more biologically realistic spiking neural network model to simulate the hierarchical representation of object shape features in the primate ventral visual pathway. The results show that, under unsupervised competitive learning and STDP, the network can self-organize to form neurons selectively responsive to local boundary contour elements, and that polychronization emerges in higher layers, giving rise to hierarchical feature binding (HFB) circuits that encode visual feature relationships across different spatial scales. However, some issues still remain to be further addressed.

1. The introduction is somewhat verbose and could be made more concise. It would benefit from a clearer logical organization that highlights the progression of existing research. In particular, the introduction lacks sufficient biological explanation of feature binding, which is central to the study. Providing more detail on the biological basis and experimental evidence for feature binding would strengthen the motivation and contextual grounding of the work.

We thank the reviewer for these suggested changes. The Introduction has been extensively rewritten to reduce length, improve progression, and strengthen biological motivation for feature binding. We cut tangential material and reorganised the section so that it moves from (i) the binding problem and the specific open mechanistic gap, to (ii) a compact biological grounding for hierarchical representations and binding, and then to (iii) the modelling progression from Eguchi et al. (2015) to binding by polychronization (Eguchi et al., 2018; Isbister et al., 2018), culminating in the study questions. To address the biological explanation directly, we added a concise paragraph outlining classic theoretical motivation for binding (the “superposition catastrophe”; Rosenblatt, 1962; Von der Malsburg, 1999), a brief summary of influential binding proposals (feature integration theory: Treisman & Gelade, 1980; synchrony-based binding: Kreiter & Singer, 1996) and a key limitation for strict synchrony under heterogeneous axonal delays (e.g., Diesmann et al., 1999). We then added direct neurophysiological motivation for relational and temporal codes, including border-ownership signals as an explicit example of a binding relation in early visual cortex (Zhou et al., 2000) and evidence for reliable spatiotemporal firing patterns beyond rate codes (Abeles et al., 1993; Prut et al., 1998), which motivates timing-based schemes such as polychronization (Izhikevich, 2006). This revised structure is intended to give a clearer biological rationale for the binding mechanism studied here while keeping the Introduction concise.

2. While the study reports phenomena such as the bimodal distribution of STDP, it does not provide robustness analyses with respect to key model parameters (e.g., learning rate, delay distribution range, or the strength of competitive inhibition). Including such analyses to demonstrate that the observed results remain stable across parameter variations would substantially strengthen the validity and persuasiveness of the conclusions.

We agree that demonstrating robustness to key parameter choices strengthens the conclusions. We have therefore added a new robustness analysis just before the Discussion (“Robustness to key model parameters”, lines 1142-1148, Supplementary Fig. S9) in which we sweep (i) the strength of inhibitory competition (I->E), (ii) the maximum excitatory conduction delay, and (iii) the STDP learning rate. For each parameter setting we quantify (A) the rank-ordered stimulus-specific information carried by all L4 excitatory neurons (convexity at the preferred side), (B) the corresponding number (and percentage) of informative output neurons exceeding 2/3 bits, and (C) the number of detected three-neuron HFB-structured PNGs

spanning the final two layers. Results are averaged across three independent trials, with s.e.m. shown for the summary counts. Across all sweeps, the ranked information curves and both summary measures vary smoothly around the default regime, indicating that the emergence of feature selectivity and the detection of HFB-structured PNGs do not depend on finely tuned parameter settings.

3. Although the construction methods of the N3P2 and N4P2 datasets are explained, the manuscript could benefit from the inclusion of additional illustrative examples or statistical summaries (e.g., proportions of different shapes, measures of feature diversity). Such additions would help readers better appreciate why these datasets can be regarded as "ecologically realistic."

In response, we have added a statistical summary of the N3P2 and N4P2 datasets in the "Training on visual stimuli and inference" section (lines 515-527). Specifically, we now report (i) the exact balance of convex vs. concave boundary elements at each object side (50% each), (ii) equal representation of all four joint configurations for any pair of sides, and (iii) the distribution of the number of convex sides per shape (N3P2: 1, 3, 3, 1; N4P2: 1, 4, 6, 4, 1). We also clarify how these statistics support our use of the term "ecologically realistic" for the modelling objective: a small shared alphabet of boundary parts is reused across many distinct shapes, so each part is seen across multiple contexts, satisfying the condition underlying the statistical decoupling argument and promoting the emergence of feature-based representations under competitive learning.

4. Although the manuscript cites neurophysiological studies [8–10], it would be more helpful to include a direct "experimental results vs. simulation results" comparison in the figures. The Introduction emphasizes the importance of comparing with biological data (refs. [8–10]), and the Results section also notes consistency with real biological findings. However, adding an intuitive comparison figure or providing specific quantitative data would better highlight the biological plausibility of the model.

We thank the reviewer for this suggestion.

While a direct quantitative comparison is challenging because the model is an abstraction of general cortical principles rather than a reconstruction of a specific animal study, we believe the qualitative and functional alignment is significant, as described in the Introduction.

Our simulation results demonstrate that when incorporating four fundamental biological constraints: (i) spiking dynamics; (ii) STDP; (iii) heterogeneous axonal transmission delays; and (iv) a mix of bottom-up, top-down, and lateral connectivity, the network consistently evolves HFB circuits. These circuits provide a mechanistic explanation for the emergence of specialised cells observed in vivo.

One type of neuron found in the monkey visual brain that may be an example of such a binding neuron is a border ownership cell found in cortical area V2. We have referenced work describing border ownership cells in lines 72-80 as part of the revised introduction. We have also toned down our language in the introduction (lines 31-32) in regard to drawing comparisons with experimental studies.

5. The conclusions and results would benefit from a more explicit discussion in relation to biological phenomena. In particular, it would be valuable to highlight the biological implications or to compare with studies on feature binding in real neurons. For example, recent work

(https://doi.org/10.1016/j.neunet.2025.107555; https://doi.org/10.3389/fncel.2015.00067) has shown that single-neuron nonlinear properties can endow neurons with feature binding capabilities. Incorporating such comparisons and biological insights would not only contextualize the findings but also strengthen the relevance of the model to neuroscience.

We have added a discussion (see Discussion, "PNGs form circuits which encode hierarchical feature binding relationships", lines 1254-1266) that more explicitly situates our proposed hierarchical feature binding mechanism in relation to biological accounts of binding at the single-neuron level. We now cite this recent work showing that nonlinear dendritic integration can support feature binding within individual neurons, depending on the mode of dendritic summation and the distribution of synaptic inputs (e.g., clustered vs. scattered). We then clarify how our account is complementary: rather than focusing on within-neuron combined-feature selectivity, our model addresses hierarchical, circuit-level binding expressed through reproducible polychronous timing relationships between feature-selective neurons across stages, thereby capturing "part-of" relations between lower- and higher-level features.

6. The binding neuron circuit hypothesized in the manuscript (comprising three neurons) appears somewhat idealized. It may be necessary to consider whether larger-scale circuits or the involvement of inhibitory neurons are required. At present, this aspect has not been discussed.

We thank the reviewer for this point. We have now added text in the Discussion (lines 1297-1316) noting that the three-neuron HFB circuit is intentionally treated as a minimal, idealised motif selected for computational tractability. We also discuss how the same binding principle is expected to extend to larger-scale binding ensembles, for example by binding a high-level feature to multiple lower-level features via multiple binding neurons in a nested architecture. Finally, we clarify that although HFB circuits are defined here by excitatory connectivity, they operate within the model's wider lateral inhibitory circuitry, which enforces sparseness and competition and provides a stabilising context that supports the emergence and selectivity of these motifs.

7. Although the manuscript explains STDP and the network structure, some training details (e.g., initial weight distribution, implementation of inhibition) are described only briefly, which limits reproducibility. In addition, while examples of neuronal selectivity and polychronization are shown, systematic quantitative analyses (e.g., proportions of selective neurons or PNGs consistent with HFB structures) are lacking. Including such statistics or summary figures would greatly strengthen the study

The paragraph describing the training details (i.e., implementation of lateral inhibition) has been expanded on 454-460. The initial weight distributions are described in lines 538-541. In addition, we have made the codebase publicly available, including the exact configuration files used for the experiments, so that all training details can be reproduced directly.

We have strengthened the systematic quantification of single-neuron selectivity in the Results. Specifically, we (i) corrected and clarified the rank-ordered stimulus-specific information analysis (Fig. 9A; see our response to reviewer 1 point 3.1), and (ii) expanded the threshold-based summary of selective neurons (Fig. 9B) by reporting both absolute counts and the corresponding percentages of L4 excitatory neurons (n = 4096) exceeding the information threshold, with error bars showing SEM across three independent trials. Figs. 11 and 13 also include these population statistics. These additions provide an explicit estimate of the

proportion of selective output neurons across datasets and architectures. We have also added supplementary summary statistics for concave selectivity and for side-resolved information curves (Supplementary Figs. S1, 3, 4).

In addition, we include a stage-wise breakdown of the PNG detection pipeline (Detected -> Eligible -> Significant) that reports retention fractions after enforcing delay-matched HFB eligibility criteria and surrogate-based significance testing (Supplementary Fig. S5). Finally, we add new quantitative analysis showing how often high-level feature neurons participate in multiple PNGs across layers (Supplementary Fig. S8).

8. Although the manuscript emphasizes the biological plausibility of the model, its limitations (e.g., reliance on 2D shapes, omission of V1, absence of dynamic visual inputs) are not sufficiently discussed. Addressing these in the Discussion would provide a more balanced and complete perspective

We have addressed this point by revising the Discussion to clarify the study's limitations and scope (see new subsection "Limitations and scope", line 1413). In particular, we now state explicitly that our experiments prioritise experimental control using a restricted, static set of 2D shapes at a fixed retinal location, and therefore do not test invariance to common transformations (e.g., translation, rotation, scale) or more naturalistic, temporally structured inputs. We also clarify that V1 is not modelled explicitly, but approximated using a fixed Gabor filter bank driving Poisson input spike trains, and we note the V1 microcircuit dynamics and complex-cell-like invariances that are therefore omitted.

9. Some of the figures, particularly the first few, could be combined or reorganized. This would help reduce redundancy and make it easier for readers to follow the results and comparisons more clearly.

We thank the reviewer for this suggestion. In the revised manuscript we have reduced redundancy and improved the flow by combining and reorganising figures that present closely related results. Specifically, we merged the original Figs. 1-2, 10-11, 15-16, and 21-22 into composite figures with updated captions, allowing direct side-by-side comparison within a single view and reducing repeated figure references. We applied the same principle to the newly added Supplementary figures, combining related analyses where possible to support clearer comparisons and a smoother narrative.

10. There is a repeated phrase ("feature feature neurons"); the manuscript should be carefully checked for similar duplication errors throughout.

We corrected the duplicated phrase ("feature feature neurons") and performed an additional pass through the manuscript to remove similar duplication errors.

11. The term "Poisson-distributed" is inconsistently written as "Poisson distributed" in some places; please unify the terminology.

We have standardised the terminology throughout the manuscript, using the hyphenated form "Poisson-distributed" consistently.

12. Some sentences are overly long (particularly in the Implications for neuroscience and AI section); breaking them into shorter sentences would improve readability.

We have shortened and split the overly long sentences in the "Implications for neuroscience and AI" section to improve readability.

Reviewer 3

The authors simulated a multi-layer spiking neural network (SNN) model of the macaque ventral visual stream, trained using unsupervised competitive learning based on STDP, to study the formation of polychronous neuronal groups (PNGs) that represent hierarchical binding of visual features.

By applying the SPADE method to the simulated spike train data, the authors detected PNGs and further explored their dynamic and information-representational properties.

They concluded that the formation of these PNGs through unsupervised STDP learning supports the feasibility of polychrony as a potential mechanism for hierarchical feature binding in biological networks.

While I highly acknowledge that the SNN model the authors have developed and the spatio-temporal spiking dynamics exhibited by the network are intriguing and useful for testing various hypotheses about temporal aspects of spike-based information processing, I also find that many of the presented results, especially those regarding hierarchical feature binding, are still at a preliminary level.

Below, I describe my concerns about the study and suggest further analyses for more stringent argumentation.

Major points

The study is centered on the authors' hypothesis of binding by polychrony, which proposes that, for each combination of features, a binding neuron forms a PNG with the neurons representing those features.

I have fundamental doubts about the biological feasibility of this mechanism, as it would likely result in a combinatorial explosion in the number of binding neurons required to represent all possible feature combinations.

This difficulty is mitigated in the case of the present study, i.e., feature binding only between two adjacent hierarchy levels, where only pairwise binding is discussed.

Nevertheless, the mechanism still postulates a binding neuron on the upper hierarchy level corresponding to each and every feature on the lower level, resulting in a whole "copy" of the lower-level feature representation.

In fact, the authors discuss the possibility of such representation with the term "holographic principle" in the "Future work" section of the Discussion.

However, the authors did not explicitly mention that the holographic representation is a direct consequence of the binding by polychrony hypothesis as explained above, which means that, if binding by polychrony is at work in the present model as the authors insist, the holographic representation should already be instantiated there.

In the current manuscript, the authors only demonstrated the existence of PNGs representing hierarchical binding, without addressing what features they represent and how complete the set of those representations is.

I think that elucidating these points in the present model is crucial for the manuscript, as they concern the fundamental weak point of the hypothesis as I mentioned above, and once they are elucidated, this would have strong functional implications for visual information processing in artificial and biological networks.

We thank the reviewer for their considered feedback on our proposed hierarchical feature binding mechanism. The reviewer raises important concerns about the potential for combinatorial growth in the number of binding relationships, and the completeness of the representations captured by the PNG/HFB analysis. We have made efforts to address these concerns in the manuscript, and we provide an explanation here.

The theory of hierarchical feature binding could appear to require infeasibly large numbers of binding neurons that encode the hierarchical binding relationships between every possible pair of lower- and higher-level features. This might exceed the representational capacity of a neural network were it not for polychronization supporting a combinatorially large number of distinct spatiotemporal patterns (Izhikevich, 2006); this capacity, based on reuse of neuronal and synaptic elements, extends from PNGs to the feature-binding HFBs presented in this work. The manuscript provides an illustrative example of a single high-level neuron participating in multiple HFBs (now Figs. 17-18) – suggesting that there is reuse/sharing occurring within the network. In our revised submission we now quantify the participation of high-level neurons across multiple PNGs in a histogram (Supplementary Fig. S8). It shows a long tail of high-level neurons participating in multiple PNGs, with mean values in the 2–5 range. We feel that this supporting evidence mitigates the combinatorial explosion. We also note that, in general, a network need not bind every possible pair of low- and high-level features; we expect that plasticity driven by unsupervised competitive learning would select and reinforce the more salient and functionally significant feature binding circuits.

The reviewer raises a fair point that while the holographic principle is discussed in Future Work, it is not measured or rigorously analysed within our model. We do not claim full holographic completeness here; we present evidence of reuse consistent with a holographic-like implication of the hypothesis, but a full completeness analysis would require substantially broader circuit-level descriptions than the three-neuron HFB motifs analysed here. In the parts of Future Work discussing the holographic principle, we have added two citations of previous publications that tackle this concept more directly in feature-binding spiking networks. We have also removed the more speculative paragraph that extends the holographic principle into higher brain regions and working memory, which goes beyond our simulations.

Regarding the completeness of features represented, the two stimulus sets are defined entirely by their constituent elements, i.e., concave and convex boundary contour elements at different orientations. These are described in subsection “Training on visual stimuli and inference” and depicted in Figure 4. We have now added a paragraph in the resubmission (lines 515-527) to explicitly describe how combinations of these features comprise the distinct shapes. This additional text also responds to reviewer 2 point 3 to explain the extent of feature diversity supporting a level of ecological realism in the stimuli. Subsequent analysis of PNG selectivity for all convex elements is presented in Figure 14B (averaged across orientations and datasets), with selectivity for all concave elements in a new Supplementary Figure S6. Taken together, these cover the complete set of boundary-element features that the HFBs represent/bind in this stimulus regime.

Minor points

Line 495 on Page 15: "output layer" is not defined.

All previous statements of “output layer” have been changed to “final layer”. Additionally, the description of “output neurons” has been clarified on line 813.

Line 576 on Page 17: The quotation marks around the symbol s are not necessary.

The quotation marks have been removed.

Equation 14 on Page 17: Define P(r) and P(r|s), and explain how they are estimated from the data.

We thank the reviewer for drawing attention to this. We have added an explicit definition of how P(r|s) and P(r) are estimated (Methods, “Single cell information analysis”, lines 566-568): P(r|s) from the empirical firing-rate response distribution for class s over repeated presentations, and P(r) as the marginal across classes.

Line 746 on Page 21: What does "a select neuron" mean? Is it "an arbitrarily selected neuron"?

We have clarified at first mention (Results, “Formation of neurons that respond to boundary shape features”) that the neuron shown in Fig. 5A is an example neuron chosen because it exhibits strong selectivity for left-side convexity (i.e., a higher firing-rate response for left-convex than left-concave shapes). This makes clear that the panel is intended as an illustrative example of the learned selectivity observed in the trained network.

Figure 6 on Page 21: The meaning of "Untrained" and "Trained" in A needs to be explained in the caption.

We have clarified in the (revised label) Fig. 5 caption that “Untrained” refers to responses measured at the network’s initialisation, and “Trained” refers to responses measured after STDP training.

Figure 8 on Page 22: Values on the y-axis are not shown.

These values are now displayed for each of the subplots.

Figure 10 on Page 23: Curves for individual sides should be shown, instead of the mean of those.

We thank the reviewer for this suggestion, and we agree that reporting side-resolved information curves provides useful additional detail. We have addressed this in two parts. First, in the revised manuscript, Fig. 9 is now explicitly defined as a location-independent summary, where each neuron is assigned its preferred side by the maximum stimulus-specific information across sides (as described in the Methods, new lines 575-583), and neurons are rank-ordered by this maximum value. Second, we now include the detailed *side-resolved* rank-order curves in the Supplementary material (Supplementary Figs. S3-4) that is referred to from lines 830-834. This provides the performance at each object side while still retaining the pooled figure in the main text as a compact cross-architecture/dataset comparison.

Line 811 on Page 23: What is the rationale behind the threshold of 2/3 bits?

We have added a rationale in the main text where the threshold is first introduced (lines 816-821). Because the selectivity task is binary (convex vs. concave at a given object side), the maximum stimulus-specific information is 1 bit. We therefore set the “informative neuron”

threshold to 2/3 bits (≥67% of the theoretical maximum) as a conservative cutoff intended for summary visualisation purposes.

Line 812 on Page 23: "their preferred convex boundary" here can be misleading, since a neuron may prefer either convex or concave, not only convex.

We thank the reviewer for raising this point, and accordingly have clarified the selection rule to avoid implying that neurons cannot prefer concavity. In the Methods ("Single cell information analysis", lines 575-583) we now define each neuron's preferred side as the one providing its maximum stimulus-specific information, and we report convex-selective information only when the neuron's mean firing rate is higher for convex than concave at that side (otherwise the convex-selective value is set to zero). This makes explicit that "preferred convex boundary" is how we report the convex-selective subset, and that the same method applies to concave selectivity by swapping stimulus labels (new results examining concave selectivity are reported in the Supplementary, Figs. S1-4 and Fig S6).

Line 822 on Page 23: "output neuron" is not defined.

We have defined "output neurons" at first use in the Results ("Single neuron information analysis demonstrates increased feature selectivity", lines 813-814): throughout this section, "output neurons" refers to the excitatory neurons in the final layer (L4).

Figure 13 on Page 25: Explain what the inset panels in A represent in the caption.

We have revised the caption of (in revised manuscript) Fig. 11(A) to explicitly explain the inset panels. The caption now states that the inset images show an example N4P2 test shape with the corresponding level of Gaussian noise applied for each σ condition, providing a visual reference for the input degradation associated with each curve.

Lines 923-4 on Page 27: The conclusion here is derived from only one sample per architecture. More samples and a statistical test would be necessary to draw this conclusion.

We have clarified the sample size in the main text and the Fig. 14 caption; the data were collected from three independent simulations (n=3) per architecture, not a single run. To address statistical testing, we performed a two-way ANOVA on layers L2-4 (excluding the stochastic input layer) which confirmed a highly significant effect of architecture for both datasets (N3P2: $F(1,12) = 242.2$; N4P2: $F(1,12) = 512.1$; both $p < 0.001$), as well as a significant interaction with layer depth. We have updated the manuscript to explicitly state the sample size and include these statistics (lines 962-969).

Lines 924-8 on Page 27: This part is only speculation, although it is written with definitive expressions.

We acknowledge these statements are not supported by measurements and so we have removed them. This also addressed reviewer 1 point 4.2.

Line 933 on Page 27: What the "it" in "a positive identification of it" refers to is not clear.

This has been clarified on line 988: "…a positive identification of that element."

Figure 6 on Page 27: The results in support of what is written in the last sentence of the caption are not shown.

We assume that this refers to Figure 16 in the original submission. The statement regarding untrained networks having F1 scores of “effectively zero” follows from these networks having produced a negligible number of PNGs, rendering F1 analysis impractical. To support this claim visually and avoid confusion, we have combined the PNG count figure and the representational performance figure into a single composite figure (Fig. 14 in the revised submission).

We have revised the caption to explicitly link these two panels. Specifically, we removed the sentence "...without which the F1 scores are effectively zero" to avoid implying that results were hidden. Instead, the caption now states: "As shown by the negligible counts in (A), STDP training is necessary for PNG emergence," which clarifies why performance metrics are not plotted for the untrained condition.

Lines 962-4 on Page 28: This part is written as if it reports a novel observation in the analysis result, but it is actually the requirement for the HFB circuits to be detected with the analysis method used here.

We have removed the two last sentences that suggested novel observations.

Figure 20 on Page 29: The figure would be more illustrative if the two network diagrams overlapped with each other at neuron H (i.e., flip A horizontally, and combine A and B such that neuron H in the two diagrams are on top of each other).

We thank the reviewer for this suggestion. This figure has since been replotted (now Fig. 18), such that these two network diagrams overlap with each other at neuron H (the common high-level feature neuron).

Figure 21 on Page 30: In B, the median should be plotted rather than the mean, to be consistent with Figure 22.

We chose the mean in (original submission) Fig 21B because dispersion is quantified as a standard deviation; mean and SD form a matched summary pair. We therefore revised (originally) Fig. 22 to report mean onset as well, ensuring consistent reporting across figures. We have also combined these two figures together (now Fig. 19) to make it easier for the reader to follow these results and to draw comparisons.

Line 1041 on Page 30: Why is the influence in this particular direction focused here? I think the influence in the other direction is just as feasible. In any case, it would be appropriate here to examine only the degree of correlation between the two, without assuming any particular direction of influence.

We agree that the relationship should be treated as correlational rather than implying a direction of influence. We revised the text (lines 1102-1108) to describe this as an association between PNG onset time and selectivity (F1), and updated the figure to report effect size and uncertainty (slope with 95% CI) together with goodness-of-fit statistics, without causal language. The revised analysis shows a weak but reliable negative association (slope = -4.3 ms per 0.1 F1, 95% CI [-5.9, -2.8]; R^2=0.083; p = 6x10^-8; n=340). We now explicitly state in the text that this analysis does not assume a direction of influence.

Figure 22 on Page 31: Why are there so few data points compared to Figure 21B? The authors should show here a simple scatter plot of F1 score vs. median onset of all detected PNGs.

This has now been addressed by plotting the unaggregated scatter: each point corresponds to a PNG-side pairing (onset time averaged over first-onset occurrences for that PNG when responding to one of the convex sides). The earlier version of Fig 22 displayed binned/aggregated summaries across F1 intervals, which reduced the number of plotted points; the revised Fig. 19C shows all datapoints directly. As described above, we now consistently report mean onset rather than median.
