# Supplementary material for: Hierarchical feature binding in a spiking neural network model of the primate ventral visual pathway
Source: PLoS Comput Biol. 2026 Sep 16;22(9):e1014752. doi: 10.1371/journal.pcbi.1014752 (PMC13581216; doi:10.1371/journal.pcbi.1014752)
Supplement: Supplementary file 13 [file pcbi.1014752.s014.pdf]

Response to Reviewers

Dear Editor and Reviewers,

We thank the Editor and Reviewers for their continued consideration of our manuscript and for the further feedback on the first revision. We are glad that the previous revision was regarded as a substantial improvement, and we have concentrated this second revision on the key remaining concern, namely a fuller analysis and discussion of neuronal reuse, the capacity of the binding mechanism, and the completeness of the feature representation. We provide a point-by-point response below. Where possible, we indicate where changes were made in the revised manuscript using figure and line numbers (based on the clean revised manuscript), and all changes are also visible in the tracked-changes version.

Kind regards,

Brian Gardner on behalf of all the authors

Overview of key changes in the revised manuscript:

- Extended the Results on polychronization with a new analysis of neuronal reuse across all three circuit roles (low-level, high-level and binding neurons), the switching of neurons between these roles, and the resulting binding-neuron ambiguity together with its resolution at the level of the time-locked triplet. This is presented in a renamed and expanded subsection (lines 1043+), a new main-text figure (Fig. 19), and the expanded S8 Fig.
- Added a new Results subsection (lines 1115+) quantifying how completely the binding circuits represent the feature repertoire, by comparing the recruited low-level neurons against the informative low-level population with their reuse accounted for, together with the convex bias of this representation (new S9 Fig).
- Expanded the Discussion with a new subsection on the functional implications of neuronal reuse for the representational capacity and plausibility of binding-by-polychrony (lines 1423+).
- Expanded the Discussion treatment of the representational bias towards convex boundary elements, setting out its architectural origin (lines 1302-1317), and revised the related Results text for consistency.
- Adjusted several claims in light of the new analyses, softening the closing statement of the abstract and adding a passage to Limitations and scope (lines 1547-1556) noting that we do not model an explicit downstream read-out of the binding and that the representations correspond to an intermediate, V4-like stage of the ventral hierarchy.
- In total, one new main-text figure and one new supplementary figure (S9 Fig) have been added and one supplementary figure expanded (S8 Fig); the former S9 Fig (sensitivity to key network parameters) is correspondingly renumbered to S10 Fig.
- Extended the publicly available codebase with new code and documented notebooks that reproduce the additional analyses and figures.

Reviewer 1

The revised manuscript represents a substantial improvement over the original submission. The authors have addressed many of the earlier concerns, particularly with respect to clarity, methodological transparency, and additional analyses. My initial assessment of the work was positive, and I expected that the main issues could be resolved within revision.

However, the revision also clarifies the scope of the approach and brings into sharper focus several limitations that remain only partially addressed. These include the absence of benchmarking against alternative models, the limited assessment of generalization (e.g., invariance), and open questions regarding the scalability and completeness of the proposed binding mechanism. In several cases, these issues are acknowledged in the discussion but not directly tested.

Given that these remaining points would likely require substantial additional work rather than incremental revision, I leave it to the editor to determine whether the current level of evidence is sufficient for publication in PLOS Computational Biology.

We thank the reviewer for their positive overall assessment of our contribution, and for identifying the points that remain. We address the most substantive of these, scalability and completeness, and note that this concern is shared with Reviewer 3 - so the changes described below relate to both reviews.

On scalability, the central question is whether the binding mechanism requires a dedicated neuron for every feature relationship, which would not scale. Our new analysis of neuronal reuse shows that it does not: because a binding relationship is carried by the time-locked firing pattern of a circuit rather than by any single neuron, individual neurons are reused across many circuits, and the network represents far more binding relationships than it has neurons. We relate this to the representational capacity of a polychronous code in a new Discussion subsection, “Reuse of binding circuits reflects the capacity of a polychronous code” (lines 1423+), with the supporting analyses in the expanded Results subsection (lines 1043+; Fig. 19, expanded S8 Fig). On completeness, we now quantify how fully the binding circuits represent the feature repertoire, in a new Results subsection (lines 1115+) and the new S9 Fig; we find that a considerable portion of the informative low-level features are represented and that coverage is complete at moderate selectivity (neurons recruited into PNGs with an F1 score > 0.7). We provide a fuller account of both analyses in our responses to Reviewer 3’s first two points.

On the remaining two points, benchmarking against alternative models such as VisNet, and the assessment of invariance to transformations, we share the reviewer’s view that both would require substantial additional work. As in our previous revision, we therefore regard these as separate studies in their own right rather than additions to the present one, each requiring model training and evaluation with transformed stimuli and an invariance-learning mechanism. We identify them as the natural next steps, as set out in “Limitations and scope” and in “Future work”.

Reviewer 2

Thank you for the revisions. I have no further concerns

We are glad the reviewer has no remaining concerns, and we are grateful for their constructive engagement in the previous round of review.

Reviewer 3

While I acknowledge that all the minor concernes raised in my previous report are all properly addressed, I consider that the revised manuscript still lacks sufficient additional analyses and discussions regarding my major concern.

We thank the reviewer for their continued and careful assessment of our work. We are glad that our previous revision resolved the minor points raised, and we have focused this revision on the major concern, which we address point by point below.

One of the major points in my report was that "the mechanism still postulates a binding neuron on the upper hierarchy level corresponding to each and every feature on the lower level, resulting in a whole "copy" of the lower-level feature representation". In response to this comment, the authors added a new supplementary figure (S8 Fig) showing the degree of "reuse" of high-level feature neurons in the PNGs. However, such a reuse does not contribute to reducing the required number of binding neurons; as many number of binding neurons as the number of PNGs are still required, resulting in a whole "copy" of the low-level features participating in the HFBs represented by the PNGs.

Furthermore, if the reuse is so common, there can be cases of reuse of low-level features, which *increases* the required number of binding neurons given a number of low-level feature neurons. To make the matter more complicated, there should also be cases of reuse of binding neurons, and cases where one neuron is a high-level feature neuron in one PNG and a binding neuron in another PNG.

Such reuses must be actually happenning in the network, because Fig. 14A shows that the number of PNGs in the network trained with the N4P2 dataset exceeds 4096, i.e., the total number of excitatory neurons in one layer. Without the reuse of binding neurons, such a high number cannot be achieved, since, as mentioned above, the reuse of only high-level feature neurons still requires the same number of binding neurons as the number of PNGs, which exceeds the total number of excitatory neurons in a layer.

Based on these considerations, I conclude that the reuse analysis presented in the current manuscript is incomplete; it only provides a biased view on the reuse of functional roles in the HFB circuit. The authors should analize the reuse of low-level feature neurons and binding neurons as well, and further elucidate the functional implications of the obtained results. In particular, the reuse of binding neurons seems to raise a fundamental doubt about the plausibility of the binding-by-polychrony, because this allows firing of a binding neuron to represent binding of different combinations of low- and high-level features. I consider this point to deserve a thorough analysis and discussion.

We appreciate the reviewer's reasoned consideration regarding this major point, and we realise that our previous analysis, which quantified only the reuse of high-level feature neurons, gave an incomplete picture of reuse in the circuit. In this revision, we have addressed this by analysing reuse across all three circuit roles (low-, high-, and binding-neurons), including the switching of neurons between roles, and we have set out the functional implications. These results are reported in a renamed and substantially expanded Results subsection, "Reuse of feature and binding neurons preserves selective polychronous patterns" (lines 1043+), along with a new main-text figure (Fig. 19) and the expanded S8 Fig, which now reports reuse across all three roles; their implications are discussed in a new Discussion subsection, "Reuse of

binding circuits reflects the capacity of a polychronous code” (lines 1423+). In assembling this subsection we relocated and merged two paragraphs from the previous Discussion, added in the first revision, on the minimal three-neuron motif and the regulatory role of lateral inhibition (now lines 1457-1472); their substance is kept, though condensed, with the account of larger-scale circuits updated to reflect the reuse described above.

The reviewer’s reasoning is supported by these analyses. Since the number of PNGs in the N4P2 network exceeds the 4096 excitatory neurons of a layer (Fig. 19A), the binding neurons must be, and indeed are, reused: with each layer supporting between 6300 to 7300 PNGs, each binding neuron participates in several circuits, with a reuse factor of about 4 to 5. The low-level and high-level neurons are reused in the same way, and neurons frequently switch roles between circuits: within a layer, about two-thirds to three-quarters of the high-level neurons also act as binding neurons in other circuits, and about 84% of the high-level and binding neurons in layers 2 and 3 additionally serve as the low-level neuron of a circuit one layer above (expanded S8 Fig). We demonstrate that the binding role must therefore be contextual rather than an intrinsic property of a neuron.

This reuse does make the individual binding neuron ambiguous: a reused binding neuron fires across circuits that encode different feature combinations, and at deeper layers it is often individually uninformative about any single feature (Fig. 19B). This ambiguity does not, however, degrade what the circuits represent. A circuit’s selectivity for its preferred feature is essentially invariant to the number of distinct features its binding neuron participates in (Fig. 19C), and strongly selective circuits persist even where the binding neuron does not itself specify the bound feature, accounting for about 40% of the most selective circuits at the deepest layer (Fig. 19D). As such, the bound feature must therefore be carried by the time-locked co-firing of the three neurons rather than by the binding neuron alone, so a binding neuron that participates in different combinations is the behaviour expected of a coincidence detector (i.e., it activates when it receives delay-matched spikes from its low- and high-level partners) - rather than a shortcoming of the scheme.

In this way, we regard reuse as not being a hindrance to the mechanism, but rather the means by which it achieves its representational capacity. Because a binding relationship resides in the coordinated firing pattern, a single binding neuron can serve many relationships, so the distinct binding-neuron population is far smaller than the set of relationships it represents, and no full copy of the lower-level representation is required at the upper level; the same reasoning addresses the concern that reusing low-level features would increase the number of binding neurons required. The network thereby represents far more binding relationships than it has neurons, which we relate to the representational capacity of a polychronous code in the new Discussion subsection (lines 1423+).

Another point I found missed to be addressed in the authors' response is about my comment: "the authors only demonstrated the existence of PNGs representing hierarchical binding, without addressing what features they represent and how complete the set of those representations is." In response to this, the authors referred to Fig. 14B, which shows a rank-ordered F1 scores of PNGs. In the main text, this figure is described as representing that "between 10 and 40 PNGs in each task achieved high F1-score (> 0.9)." One cannot judge if this number ("between 10 to 40 PNGs") implies the completeness of feature representation by PNGs.

One simple way of quantifying the completeness would be to compare this number to the total number of informative neurons in the lower layer. If the low-level feature neurons of PNGs comprises a considerable portion of the informative neurons in the lower layer, this indicates a certain degree of completeness (though the reuse of low-level feature neurons need to be taken into account here.) Otherwise, the authors should examine whether PNGs are biased to particular low-level features, or all low-level features are uniformly sparsely represented by PNGs.

We recognise that the number of high-F1 PNGs alone does not establish completeness, and so we have replaced it with the comparison the reviewer suggests. In a new Results subsection, "Binding circuits cover the feature repertoire at moderate selectivity" (lines 1115+), supported by the new S9 Fig, we compare the low-level neurons recruited into binding circuits against the informative low-level population, collapsing their reuse by counting distinct neurons and grading recruitment by circuit selectivity. A considerable portion of the informative low-level neurons enter a binding circuit, about 60% for N3P2 and 83% for N4P2, and coverage of the feature repertoire is complete at moderate selectivity - with every feature being represented by at least one circuit at $F1 > 0.7$ (six of six for N3P2, eight of eight for N4P2).

At our strictest $F1 > 0.9$ criterion the representation becomes sparse, with only about 4% of informative low-level neurons in a strongly selective circuit, but it is not uniformly sparse: both this subset and the informative pool are biased towards convex features (a convex-to-concave ratio of about 4.0 for N3P2 and 2.2 for N4P2), so the sparsity is feature-biased rather than uniform - the alternative the reviewer raises. We address the architectural origin of this convex bias in the Discussion (lines 1302-1317) and in our response to the reviewer's third point below.

Finally, regarding the newly added results about the representational bias towards concave elements, the authors discussed this finding only in one line in the Discussion, but there should be more points to be discussed regarding this. For example, S3 Fig B and S4 Fig B clearly show that this bias is evident already in the untrained networks (no concave-selective neurons at all in the untrained networks), meaning that this bias largely stems from the network architecture rather than the training procedure. The authors should discuss what feature of the network architecture causes such a strong bias.

We thank the reviewer for this point, and we agree that the convexity bias warranted fuller treatment than the single sentence we gave previously. We also accept the reviewer's central observation: because the bias is already evident in the untrained networks, where convex-selective responses dominate (Figs 9B, S1B, S3 B and S4 B), it derives from the network architecture rather than from the training procedure, with competitive learning via STDP then working on this initial asymmetry. We have expanded the Discussion with a new passage that sets out the mechanistic cause (new lines 1302-1317) along with its relation to existing experimental studies. Additionally, we have reworded the Results text that had previously described the untrained preference in terms of chance (revised lines 822-825, 833-836) so that it is consistent with this account.
