# Supplementary material for: Hierarchical feature binding in a spiking neural network model of the primate ventral visual pathway
Source: PLoS Comput Biol. 2026 Sep 16;22(9):e1014752. doi: 10.1371/journal.pcbi.1014752 (PMC13581216; doi:10.1371/journal.pcbi.1014752)
Supplement: Peer review history

# Peer review: Decision Letter 0

**DOI:** 10.1371/journal.pcbi.1014752.r001

**Authors:** Abigail Morrison, Daniele Marinazzo

10 Oct 2025

Hierarchical feature binding in a spiking neural network model of the primate ventral visual pathway

PLOS Computational Biology

Dear Dr. Gardner,

Thank you for submitting your manuscript to PLOS Computational Biology. After careful consideration, we feel that it has merit but does not fully meet PLOS Computational Biology's publication criteria as it currently stands. Therefore, we invite you to submit a revised version of the manuscript that addresses the points raised during the review process.

Please submit your revised manuscript within 60 days Dec 10 2025 11:59PM. If you will need more time than this to complete your revisions, please reply to this message or contact the journal office at ploscompbiol@plos.org. When you're ready to submit your revision, log on to https://www.editorialmanager.com/pcompbiol/ and select the 'Submissions Needing Revision' folder to locate your manuscript file.

Please include the following items when submitting your revised manuscript:

* A rebuttal letter that responds to each point raised by the editor and reviewer(s). You should upload this letter as a separate file labeled 'Response to Reviewers'. This file does not need to include responses to formatting updates and technical items listed in the 'Journal Requirements' section below.

* A marked-up copy of your manuscript that highlights changes made to the original version. You should upload this as a separate file labeled 'Revised Manuscript with Track Changes'.

* An unmarked version of your revised paper without tracked changes. You should upload this as a separate file labeled 'Manuscript'.

If you would like to make changes to your financial disclosure, competing interests statement, or data availability statement, please make these updates within the submission form at the time of resubmission. Guidelines for resubmitting your figure files are available below the reviewer comments at the end of this letter

We look forward to receiving your revised manuscript.

Kind regards,

Abigail Morrison

Academic Editor

PLOS Computational Biology

Daniele Marinazzo

Section Editor

PLOS Computational Biology

**Journal Requirements:**

1) We ask that a manuscript source file is provided at Revision. Please upload your manuscript file as a .doc, .docx, .rtf or .tex. If you are providing a .tex file, please upload it under the item type u2018LaTeX Source Fileu2019 and leave your .pdf version as the item type u2018Manuscriptu2019.

2) Some material included in your submission may be copyrighted. According to PLOSu2019s copyright policy, authors who use figures or other material (e.g., graphics, clipart, maps) from another author or copyright holder must demonstrate or obtain permission to publish this material under the Creative Commons Attribution 4.0 International (CC BY 4.0) License used by PLOS journals. Please closely review the details of PLOSu2019s copyright requirements here: PLOS Licenses and Copyright. If you need to request permissions from a copyright holder, you may use PLOS's Copyright Content Permission form.

Please respond directly to this email and provide any known details concerning your material's license terms and permissions required for reuse, even if you have not yet obtained copyright permissions or are unsure of your material's copyright compatibility. Once you have responded and addressed all other outstanding technical requirements, you may resubmit your manuscript within Editorial Manager.

Potential Copyright Issues:

- Figure 2. Please confirm whether you drew the images / clip-art within the figure panels by hand. If you did not draw the images, please provide (a) a link to the source of the images or icons and their license / terms of use; or (b) written permission from the copyright holder to publish the images or icons under our CC BY 4.0 license. Alternatively, you may replace the images with open source alternatives. See these open source resources you may use to replace images / clip-art:

- https://commons.wikimedia.org

- https://openclipart.org/.

**Reviewers' comments:**

Reviewer's Responses to Questions

**Comments to the Authors:**

**Please note here if the review is uploaded as an attachment.**

Reviewer #1: The review report is uploaded as an attachment.

Reviewer #2: This study constructed a more biologically realistic spiking neural network model to simulate the hierarchical representation of object shape features in the primate ventral visual pathway. The results show that, under unsupervised competitive learning and STDP, the network can self-organize to form neurons selectively responsive to local boundary contour elements, and that polychronization emerges in higher layers, giving rise to hierarchical feature binding (HFB) circuits that encode visual feature relationships across different spatial scales. However, some issues still remain to be further addressed.

1. The introduction is somewhat verbose and could be made more concise. It would benefit from a clearer logical organization that highlights the progression of existing research. In particular, the introduction lacks sufficient biological explanation of feature binding, which is central to the study. Providing more detail on the biological basis and experimental evidence for feature binding would strengthen the motivation and contextual grounding of the work.

2. While the study reports phenomena such as the bimodal distribution of STDP, it does not provide robustness analyses with respect to key model parameters (e.g., learning rate, delay distribution range, or the strength of competitive inhibition). Including such analyses to demonstrate that the observed results remain stable across parameter variations would substantially strengthen the validity and persuasiveness of the conclusions.

3. Although the construction methods of the N3P2 and N4P2 datasets are explained, the manuscript could benefit from the inclusion of additional illustrative examples or statistical summaries (e.g., proportions of different shapes, measures of feature diversity). Such additions would help readers better appreciate why these datasets can be regarded as “ecologically realistic.”

4. Although the manuscript cites neurophysiological studies [8–10], it would be more helpful to include a direct “experimental results vs. simulation results” comparison in the figures. The Introduction emphasizes the importance of comparing with biological data (refs. [8–10]), and the Results section also notes consistency with real biological findings. However, adding an intuitive comparison figure or providing specific quantitative data would better highlight the biological plausibility of the model.

5. The conclusions and results would benefit from a more explicit discussion in relation to biological phenomena. In particular, it would be valuable to highlight the biological implications or to compare with studies on feature binding in real neurons. For example, recent work (https://doi.org/10.1016/j.neunet.2025.107555; https://doi.org/10.3389/fncel.2015.00067) has shown that single-neuron nonlinear properties can endow neurons with feature binding capabilities. Incorporating such comparisons and biological insights would not only contextualize the findings but also strengthen the relevance of the model to neuroscience.

6. The binding neuron circuit hypothesized in the manuscript (comprising three neurons) appears somewhat idealized. It may be necessary to consider whether larger-scale circuits or the involvement of inhibitory neurons are required. At present, this aspect has not been discussed.

7. Although the manuscript explains STDP and the network structure, some training details (e.g., initial weight distribution, implementation of inhibition) are described only briefly, which limits reproducibility. In addition, while examples of neuronal selectivity and polychronization are shown, systematic quantitative analyses (e.g., proportions of selective neurons or PNGs consistent with HFB structures) are lacking. Including such statistics or summary figures would greatly strengthen the study

8. Although the manuscript emphasizes the biological plausibility of the model, its limitations (e.g., reliance on 2D shapes, omission of V1, absence of dynamic visual inputs) are not sufficiently discussed. Addressing these in the Discussion would provide a more balanced and complete perspective

9. Some of the figures, particularly the first few, could be combined or reorganized. This would help reduce redundancy and make it easier for readers to follow the results and comparisons more clearly.

10. There is a repeated phrase (“feature feature neurons”); the manuscript should be carefully checked for similar duplication errors throughout.

11. The term “Poisson-distributed” is inconsistently written as “Poisson distributed” in some places; please unify the terminology.

12. Some sentences are overly long (particularly in the Implications for neuroscience and AI section); breaking them into shorter sentences would improve readability.

Reviewer #3: The authors simulated a multi-layer spiking neural network (SNN) model of the macaque ventral visual stream, trained using unsupervised competitive learning based on STDP, to study the formation of polychronous neuronal groups (PNGs) that represent hierarchical binding of visual features.

By applying the SPADE method to the simulated spike train data, the authors detected PNGs and further explored their dynamic and information-representational properties.

They concluded that the formation of these PNGs through unsupervised STDP learning supports the feasibility of polychrony as a potential mechanism for hierarchical feature binding in biological networks.

While I highly acknowledge that the SNN model the authors have developed and the spatio-temporal spiking dynamics exhibited by the network are intriguing and useful for testing various hypotheses about temporal aspects of spike-based information processing, I also find that many of the presented results, especially those regarding hierarchical feature binding, are still at a preliminary level.

Below, I describe my concerns about the study and suggest further analyses for more stringent argumentation.

Major points

The study is centered on the authors' hypothesis of binding by polychrony, which proposes that, for each combination of features, a binding neuron forms a PNG with the neurons representing those features.

I have fundamental doubts about the biological feasibility of this mechanism, as it would likely result in a combinatorial explosion in the number of binding neurons required to represent all possible feature combinations.

This difficulty is mitigated in the case of the present study, i.e., feature binding only between two adjacent hierarchy levels, where only pairwise binding is discussed.

Nevertheless, the mechanism still postulates a binding neuron on the upper hierarchy level corresponding to each and every feature on the lower level, resulting in a whole "copy" of the lower-level feature representation.

In fact, the authors discuss the possibility of such representation with the term "holographic principle" in the "Future work" section of the Discussion.

However, the authors did not explicitly mention that the holographic representation is a direct consequence of the binding by polychrony hypothesis as explained above, which means that, if binding by polychrony is at work in the present model as the authors insist, the holographic representation should already be instantiated there.

In the current manuscript, the authors only demonstrated the existence of PNGs representing hierarchical binding, without addressing what features they represent and how complete the set of those representations is.

I think that elucidating these points in the present model is crucial for the manuscript, as they concern the fundamental weak point of the hypothesis as I mentioned above, and once they are elucidated, this would have strong functional implications for visual information processing in artificial and biological networks.

Minor points

Line 495 on Page 15: "output layer" is not defined.

Line 576 on Page 17: The quotation marks around the symbol s are not necessary.

Equation 14 on Page 17: Define P(r) and P(r|s), and explain how they are estimated from the data.

Line 746 on Page 21: What does "a select neuron" mean? Is it "an arbitrarily selected neuron"?

Figure 6 on Page 21: The meaning of "Untrained" and "Trained" in A needs to be explained in the caption.

Figure 8 on Page 22: Values on the y-axis are not shown.

Figure 10 on Page 23: Curves for individual sides should be shown, instead of the mean of those.

Line 811 on Page 23: What is the rationale behind the threshold of 2/3 bits?

Line 812 on Page 23: "their preferred convex boundary" here can be misleading, since a neuron may prefer either convex or concave, not only convex.

Line 822 on Page 23: "output neuron" is not defined.

Figure 13 on Page 25: Explain what the inset panels in A represent in the caption.

Lines 923-4 on Page 27: The conclusion here is derived from only one sample per architecture. More samples and a statistical test would be necessary to draw this conclusion.

Lines 924-8 on Page 27: This part is only speculation, although it is written with definitive expressions.

Line 933 on Page 27: What the "it" in "a positive identification of it" refers to is not clear.

Figure 6 on Page 27: The results in support of what is written in the last sentence of the caption are not shown.

Lines 962-4 on Page 28: This part is written as if it reports a novel observation in the analysis result, but it is actually the requirement for the HFB circuits to be detected with the analysis method used here.

Figure 20 on Page 29: The figure would be more illustrative if the two network diagrams overlapped with each other at neuron H (i.e., flip A horizontally, and combine A and B such that neuron H in the two diagrams are on top of each other).

Figure 21 on Page 30: In B, the median should be plotted rather than the mean, to be consistent with Figure 22.

Line 1041 on Page 30: Why is the influence in this particular direction focused here? I think the influence in the other direction is just as feasible. In any case, it would be appropriate here to examine only the degree of correlation between the two, without assuming any particular direction of influence.

Figure 22 on Page 31: Why are there so few data points compared to Figure 21B? The authors should show here a simple scatter plot of F1 score vs. median onset of all detected PNGs.

**********

**Have the authors made all data and (if applicable) computational code underlying the findings in their manuscript fully available?**

The PLOS Data policy requires authors to make all data and code underlying the findings described in their manuscript fully available without restriction, with rare exception (please refer to the Data Availability Statement in the manuscript PDF file). The data and code should be provided as part of the manuscript or its supporting information, or deposited to a public repository. For example, in addition to summary statistics, the data points behind means, medians and variance measures should be available. If there are restrictions on publicly sharing data or code —e.g. participant privacy or use of data from a third party—those must be specified.

Reviewer #1: **No:** The manuscript notes that implementation details are available, but the provided code is hosted on the University of Surrey GitLab, which is restricted to account holders and not publicly accessible. This prevents reviewers (and eventually readers) from verifying reproducibility.

Reviewer #2: Yes

Reviewer #3: **No:** The online repository indicated in the data availability statement (https://gitlab.surrey.ac.uk/bg0013/feature_binding) does not seem to be accessible by external users.

**********

PLOS authors have the option to publish the peer review history of their article (what does this mean?). If published, this will include your full peer review and any attached files.

If you choose “no”, your identity will remain anonymous but your review may still be made public.

**Do you want your identity to be public for this peer review?**  For information about this choice, including consent withdrawal, please see our Privacy Policy.

Reviewer #1: No

Reviewer #2: No

Reviewer #3: No

[NOTE: If reviewer comments were submitted as an attachment file, they will be attached to this email and accessible via the submission site. Please log into your account, locate the manuscript record, and check for the action link "View Attachments". If this link does not appear, there are no attachment files.]

**Figure resubmission:**

**Reproducibility:**

?>

Attachment Submitted filename: 20250916_Review_report.pdf

# Author comment: Author response to Decision Letter 1

**DOI:** 10.1371/journal.pcbi.1014752.r002

16 Feb 2026

Attachment Submitted filename: Response_to_Reviewers.pdf

# Peer review: Decision Letter 1

**DOI:** 10.1371/journal.pcbi.1014752.r003

**Authors:** Abigail Morrison, Daniele Marinazzo

24 Apr 2026

PCOMPBIOL-D-25-01408R1

Hierarchical feature binding in a spiking neural network model of the primate ventral visual pathway

PLOS Computational Biology

Dear Dr. Gardner,

Thank you for submitting your manuscript to PLOS Computational Biology. After careful consideration, we feel that it has merit but does not fully meet PLOS Computational Biology's publication criteria as it currently stands. Therefore, we invite you to submit a revised version of the manuscript that addresses the points raised during the review process.

In particular, reviewers 1 and 3 both note that significant additional work would need to be carried out to substantiate the conclusions of the paper, and that the current set of revisions do not fully address their major concerns.

Please submit your revised manuscript by Jun 24 2026 11:59PM. If you will need more time than this to complete your revisions, please reply to this message or contact the journal office at ploscompbiol@plos.org. When you're ready to submit your revision, log on to https://www.editorialmanager.com/pcompbiol/ and select the 'Submissions Needing Revision' folder to locate your manuscript file.

Please include the following items when submitting your revised manuscript:

* A letter that responds to each point raised by the editor and reviewer(s). You should upload this letter as a separate file labeled 'Response to Reviewers'. This file does not need to include responses to formatting updates and technical items listed in the 'Journal Requirements' section below.

* A marked-up copy of your manuscript that highlights changes made to the original version. You should upload this as a separate file labeled 'Revised Manuscript with Track Changes'.

* An unmarked version of your revised paper without tracked changes. You should upload this as a separate file labeled 'Manuscript'.

If you would like to make changes to your financial disclosure, competing interests statement, or data availability statement, please make these updates within the submission form at the time of resubmission. Guidelines for resubmitting your figure files are available below the reviewer comments at the end of this letter

We look forward to receiving your revised manuscript.

Kind regards,

Abigail Morrison

Academic Editor

PLOS Computational Biology

Daniele Marinazzo

Section Editor

PLOS Computational Biology

**Reviewers' comments:**

Reviewer's Responses to Questions

**Comments to the Authors:**

**Please note here if the review is uploaded as an attachment.**

Reviewer #1: The revised manuscript represents a substantial improvement over the original submission. The authors have addressed many of the earlier concerns, particularly with respect to clarity, methodological transparency, and additional analyses. My initial assessment of the work was positive, and I expected that the main issues could be resolved within revision.

However, the revision also clarifies the scope of the approach and brings into sharper focus several limitations that remain only partially addressed. These include the absence of benchmarking against alternative models, the limited assessment of generalization (e.g., invariance), and open questions regarding the scalability and completeness of the proposed binding mechanism. In several cases, these issues are acknowledged in the discussion but not directly tested.

Given that these remaining points would likely require substantial additional work rather than incremental revision, I leave it to the editor to determine whether the current level of evidence is sufficient for publication in PLOS Computational Biology.

Reviewer #2: Thank you for the revisions. I have no further concerns

Reviewer #3: While I acknowledge that all the minor concernes raised in my previous report are all properly addressed, I consider that the revised manuscript still lacks sufficient additional analyses and discussions regarding my major concern.

One of the major points in my report was that "the mechanism still postulates a binding neuron on the upper hierarchy level corresponding to each and every feature on the lower level, resulting in a whole "copy" of the lower-level feature representation".

In response to this comment, the authors added a new supplementary figure (S8 Fig) showing the degree of "reuse" of high-level feature neurons in the PNGs.

However, such a reuse does not contribute to reducing the required number of binding neurons; as many number of binding neurons as the number of PNGs are still required, resulting in a whole "copy" of the low-level features participating in the HFBs represented by the PNGs.

Furthermore, if the reuse is so common, there can be cases of reuse of low-level features, which *increases* the required number of binding neurons given a number of low-level feature neurons.

To make the matter more complicated, there should also be cases of reuse of binding neurons, and cases where one neuron is a high-level feature neuron in one PNG and a binding neuron in another PNG.

Such reuses must be actually happenning in the network, because Fig. 14A shows that the number of PNGs in the network trained with the N4P2 dataset exceeds 4096, i.e., the total number of excitatory neurons in one layer.

Without the reuse of binding neurons, such a high number cannot be achieved, since, as mentioned above, the reuse of only high-level feature neurons still requires the same number of binding neurons as the number of PNGs, which exceeds the total number of excitatory neurons in a layer.

Based on these considerations, I conclude that the reuse analysis presented in the current manuscript is incomplete; it only provides a biased view on the reuse of functional roles in the HFB circuit.

The authors should analize the reuse of low-level feature neurons and binding neurons as well, and further elucidate the functional implications of the obtained results.

In particular, the reuse of binding neurons seems to raise a fundamental doubt about the plausibility of the binding-by-polychrony, because this allows firing of a binding neuron to represent binding of different combinations of low- and high-level features.

I consider this point to deserve a thorough analysis and discussion.

Another point I found missed to be addressed in the authors' response is about my comment: "the authors only demonstrated the existence of PNGs representing hierarchical binding, without addressing what features they represent and how complete the set of those representations is."

In response to this, the authors referred to Fig. 14B, which shows a rank-ordered F1 scores of PNGs.

In the main text, this figure is described as representing that "between 10 and 40 PNGs in each task achieved high F1-score (> 0.9)."

One cannot judge if this number ("between 10 to 40 PNGs") implies the completeness of feature representation by PNGs.

One simple way of quantifying the completeness would be to compare this number to the total number of informative neurons in the lower layer.

If the low-level feature neurons of PNGs comprises a considerable portion of the informative neurons in the lower layer, this indicates a certain degree of completeness (though the reuse of low-level feature neurons need to be taken into account here.)

Otherwise, the authors should examine whether PNGs are biased to particular low-level features, or all low-level features are uniformly sparsely represented by PNGs.

Finally, regarding the newly added results about the representational bias towards concave elements, the authors discussed this finding only in one line in the Discussion, but there should be more points to be discussed regarding this.

For example, S3 Fig B and S4 Fig B clearly show that this bias is evident already in the untrained networks (no concave-selective neurons at all in the untrained networks), meaning that this bias largely stems from the network architecture rather than the training procedure.

The authors should discuss what feature of the network architecture causes such a strong bias.

**********

**Have the authors made all data and (if applicable) computational code underlying the findings in their manuscript fully available?**

The PLOS Data policy requires authors to make all data and code underlying the findings described in their manuscript fully available without restriction, with rare exception (please refer to the Data Availability Statement in the manuscript PDF file). The data and code should be provided as part of the manuscript or its supporting information, or deposited to a public repository. For example, in addition to summary statistics, the data points behind means, medians and variance measures should be available. If there are restrictions on publicly sharing data or code —e.g. participant privacy or use of data from a third party—those must be specified.

Reviewer #1: Yes

Reviewer #2: Yes

Reviewer #3: None

**********

PLOS authors have the option to publish the peer review history of their article (what does this mean?). If published, this will include your full peer review and any attached files.

If you choose “no”, your identity will remain anonymous but your review may still be made public.

**Do you want your identity to be public for this peer review?**  For information about this choice, including consent withdrawal, please see our Privacy Policy.

Reviewer #1: No

Reviewer #2: **Yes:** Jian Liu

Reviewer #3: No

[NOTE: If reviewer comments were submitted as an attachment file, they will be attached to this email and accessible via the submission site. Please log into your account, locate the manuscript record, and check for the action link "View Attachments". If this link does not appear, there are no attachment files.]

**Figure resubmission:**

**Reproducibility:**

?>

# Author comment: Author response to Decision Letter 2

**DOI:** 10.1371/journal.pcbi.1014752.r004

24 Jun 2026

Attachment Submitted filename: Response_to_Reviewers_auresp_2.pdf

# Peer review: Decision Letter 2

**DOI:** 10.1371/journal.pcbi.1014752.r005

**Authors:** Abigail Morrison, Daniele Marinazzo

23 Aug 2026

Dear Dr Gardner,

We are pleased to inform you that your manuscript 'Hierarchical feature binding in a spiking neural network model of the primate ventral visual pathway' has been provisionally accepted for publication in PLOS Computational Biology.

Before your manuscript can be formally accepted you will need to complete some formatting changes, which you will receive in a follow up email. A member of our team will be in touch with a set of requests.

Please note that your manuscript will not be scheduled for publication until you have made the required changes, so a swift response is appreciated.

IMPORTANT: The editorial review process is now complete. PLOS will only permit corrections to spelling, formatting or significant scientific errors from this point onwards. Requests for major changes, or any which affect the scientific understanding of your work, will cause delays to the publication date of your manuscript.

Should you, your institution's press office or the journal office choose to press release your paper, you will automatically be opted out of early publication. We ask that you notify us now if you or your institution is planning to press release the article. All press must be co-ordinated with PLOS.

Thank you again for supporting Open Access publishing; we are looking forward to publishing your work in PLOS Computational Biology.

Best regards,

Abigail Morrison

Academic Editor

PLOS Computational Biology

Daniele Marinazzo

Section Editor

PLOS Computational Biology

***********************************************************

Reviewer's Responses to Questions

**Comments to the Authors:**

**Please note here if the review is uploaded as an attachment.**

Reviewer #1: I thank the authors for their careful revision and constructive responses throughout the review process. The manuscript has improved substantially since the original submission. In particular, the new analyses of neuronal reuse, representational completeness, and the functional implications of the proposed binding mechanism (Fig. 19 and the accompanying supplementary analyses) significantly strengthen the manuscript and address my remaining concerns regarding the scalability and completeness of the proposed mechanism.

The remaining limitations, such as benchmarking against alternative models and assessing invariance, are now appropriately acknowledged and discussed, and I agree that addressing them would require substantial work beyond the scope of the present study.

I therefore have no further major concerns and support publication of the manuscript.

I have only one minor suggestion. In the discussion of the new analyses associated with Fig. 19, I encourage the authors to slightly soften a few interpretative statements. In several places, expressions such as "the bound feature is recovered at the level of the circuit" or "this follows from the binding neuron's function as a coincidence detector" could be replaced by more cautious formulations such as "the results support the interpretation that..." or "are consistent with...". This would better align the conclusions with the evidence presented while preserving the overall message of the manuscript.

Reviewer #3: "The revised manuscript properly addressed all the points I raised in my previous reports.

I have no remaining concerns."

**********

**Have the authors made all data and (if applicable) computational code underlying the findings in their manuscript fully available?**

The PLOS Data policy requires authors to make all data and code underlying the findings described in their manuscript fully available without restriction, with rare exception (please refer to the Data Availability Statement in the manuscript PDF file). The data and code should be provided as part of the manuscript or its supporting information, or deposited to a public repository. For example, in addition to summary statistics, the data points behind means, medians and variance measures should be available. If there are restrictions on publicly sharing data or code —e.g. participant privacy or use of data from a third party—those must be specified.

Reviewer #1: Yes

Reviewer #3: Yes

**********

PLOS authors have the option to publish the peer review history of their article (what does this mean?). If published, this will include your full peer review and any attached files.

If you choose “no”, your identity will remain anonymous but your review may still be made public.

**Do you want your identity to be public for this peer review?**  For information about this choice, including consent withdrawal, please see our Privacy Policy.

Reviewer #1: No

Reviewer #3: No

# Editor report: Acceptance letter

**DOI:** 10.1371/journal.pcbi.1014752.r006

**Authors:** Abigail Morrison, Daniele Marinazzo

PCOMPBIOL-D-25-01408R2

Hierarchical feature binding in a spiking neural network model of the primate ventral visual pathway

Dear Dr Gardner,

I am pleased to inform you that your manuscript has been formally accepted for publication in PLOS Computational Biology. Your manuscript is now with our production department and you will be notified of the publication date in due course.

The corresponding author will soon be receiving a typeset proof for review, to ensure errors have not been introduced during production. Please review the PDF proof of your manuscript carefully, as this is the last chance to correct any errors. Please note that major changes, or those which affect the scientific understanding of the work, will likely cause delays to the publication date of your manuscript.

Soon after your final files are uploaded, unless you have opted out, the early version of your manuscript will be published online. The date of the early version will be your article's publication date. The final article will be published to the same URL, and all versions of the paper will be accessible to readers.

For Research, Software, and Methods articles, you will receive an invoice from PLOS for your publication fee after your manuscript has reached the completed accept phase. If you receive an email requesting payment before acceptance or for any other service, this may be a phishing scheme. Learn how to identify phishing emails and protect your accounts at https://explore.plos.org/phishing.

Thank you again for supporting PLOS Computational Biology and open-access publishing. We are looking forward to publishing your work!

With kind regards,

Sharmila Kamatchi

PLOS Computational Biology | Carlyle House, Carlyle Road, Cambridge CB4 3DN | United Kingdom ploscompbiol@plos.org | Phone +44 (0) 1223-442824 | ploscompbiol.org | @PLOSCompBiol
